# Discovery of novel paeonol-based derivatives against skin inflammation *in vitro* and *in vivo*

**DOI:** 10.1080/14756366.2022.2043852

**Published:** 2022-02-28

**Authors:** Jing Wu, Ren De Zhu, Guo Min Cao, Jun Cheng Du, Xin Liu, Liang Zhuo Diao, Zhao Yan Zhang, Yang Sheng Hu, Xin Hua Liu, Jing Bo Shi

**Affiliations:** a School of Pharmacy, Anhui Medical University, Hefei, P. R. China; b Inflammation and Immune Mediated Diseases Laboratory of Anhui Province, Hefei, P. R. China; c Department of Clinical Medicine, Second Clinical Medical College, Anhui Medical University, Hefei, P. R. China; d Department of Medicine, The Fourth Affiliated Hospital of Anhui Medical University, Hefei, P. R. China

**Keywords:** T-LAK-cell-originated protein kinase, paeonol derivatives, design, synthesis, anti-inflammatory

## Abstract

T-LAK-cell-originated protein kinase (TOPK), a novel member of the mitogen-activated protein kinase family, is considered an effective therapeutic target for skin inflammation. In this study, a series (**A − D**) of paeonol derivatives was designed and synthesised using a fragment growing approach, and their anti-inflammatory activities against lipopolysaccharide (LPS)-induced nitric oxide production in RAW264.7 cells were tested. Among them, compound **B12** yielded the best results (IC_50_ = 2.14 μM) with low toxicity (IC_50_ > 50 µM). Preliminary mechanistic studies indicated that this compound could inhibit the TOPK-p38/JNK signalling pathway and phosphorylate downstream related proteins. A murine psoriasis-like skin inflammation model was used to determine its therapeutic effect.

## 1. Introduction

T-LAK cell-originated protein kinase (TOPK)^1^^,^^2^, a newly identified member of the MEK3/6-related MAPKK family, is expressed in a wide range of proliferating cells and tissues^2–8^ for the proliferation, progression, and metastasis in many cancers, including leukaemia and myeloma^9^^,^^10^. The p38 pathway, a downstream pathway of TOPK, is a key regulator of proinflammatory cytokine biosynthesis, including TNF-α, IL-1β, and COX-2, at the transcriptional and translational levels^11–13^. In recent years, many studies have shown that TOPK plays an important role in regulating p38 with anti-inflammatory effects^14–18^. Therefore, targeting the TOPK pathway is a promising strategy for to treat inflammatory diseases.

**Figure 1.**
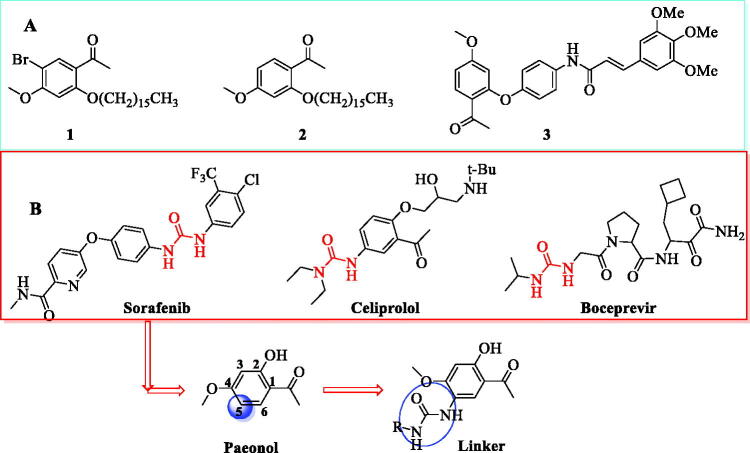
The process of design and analysis. (**A**) Some of paeonol derivatives; (**B**) Urea linker as fragment of modification in this study.

Paeonol isolated from traditional Chinese herbal medicines and used as a topical drug in China^15^^,^^16^ exhibits a wide range of biological effects, including anti-inflammatory, immune regulatory, anti-tumour, and anti-oxidative effects^18–20^. Studies have indicated that paeonol could inhibit the expression of TOPK with anti-inflammatory activity at 100 µM^16^ and a microthermophoresis (MST) assay confirmed the affinity of paeonol for TOPK. Phosphorylation levels of p38, JNKs, MSK1 and histone H2AX were suppressed by paeonol by inhibiting TOPK activity in a time-and dose-dependent manner *in vitro* and *in vivo*^17^. Although paeonol has multiple sites for structural modification, modification of the hydroxyl group is a general means. Among them, compounds **1** and **2**, reported by Huang, showed strong anti-inflammatory activity and were obtained by introducing bromine and a long alkyl chain^21^. Compound **3** showed suitable anti-inflammatory activity and was modified at its hydroxyl^22^ (Figure 1(A)). In general, the derivatives of paeonol by modification of the 2-OH or 1-acetyl groups have shown anti-inflammatory activities. However, their SARs and specific target characteristics for anti-inflammation remain unclear. Additionally, modification of the substituents at the 5-position of paeonol and evaluating their anti-inflammatory properties have rarely been reported, posing a challenging project due to their unknown anti-inflammatory activities.

Herein, we present the design and synthesis of paeonol derivatives by the introduction of a urea linker at the 5-position of paeonol so as to discover potent compounds that regulate the TOPK pathway. The urea moiety is inherent in a various clinically approved drugs such as sorafenib, celiprolol and boceprevir (Figure 1(B)) because of their strong ability to form multiple stable hydrogen bonds with protein or receptor targets^23^. Thus, a urea linker, which could modulate drug potency and selectivity and improving drug properties, was introduced at the *ortho*-position of the methoxy group. Meanwhile, to balance the activity and toxicity and explore the structure-activity relationship, the linker was substituted with an array of amines, including alkyl amines, heterocyclic/cyclic amines, and aniline (Figure 1(B)).

## 2. Experimental section

### 2.1. Materials and methods

All commercial reagents and solvents were purchased from commercial suppliers and used without further purification. Reactions were monitored by thin-layer chromatography (TLC) and visualised under UV light at 254 and 365 nm. The chromatograms were conducted on silica gel (3 0 0 ∼ 400 mesh) using combiflash chromatography systems (Teledyne ISCO R_f_ 200). ^1^H and ^13 ^C NMR spectral data were recorded with a Bruker or Agilent, 400, 600 MHz spectrometer in CDCl_3_ or DMSO-d_6_ using tetramethylsilane (TMS) as internal standard (IS). High-resolution mass spectrometry (HRMS) was recorded on an Agilent Technologies LC-TOF instrument.

### 2.2. Chemistry

#### 2.2.1. Synthesis of 1–(5-amino-2-hydroxy-4-methoxyphenyl) ethan-1-one (2)

*1–(2-hydroxy-4-methoxy-5-nitrophenyl) ethan-1-one* **(1)**. To a solution of paeonol (10 g, 0.06 mol) in acetic acid (40 ml), nitric acid (16 ml) was dropwise added under ice/water cooling and it was stirred for 3 h. The solution was poured into ice water and yellow precipitate was produced. The crude was filtered, re-crystallised with ethanol to give compound **1** (7.2 g, 57% yield) as yellow crystal.

*1–(5-amino-2-hydroxy-4-methoxyphenyl) ethan-1-one* **(2)**. Compound **1** (2 g, 10 mmol) was dissolved in methanol (40 ml) and 10% Pd/C (4% mmol) was added. The suspension was acidified to pH 3 ∼ 4 by addition of 1 M HCl solution. The mixture was vigorously stirred under hydrogen gas at atmospheric pressure about 4 h. Upon completion of the reaction (as determined by TLC), the catalyst was removed by filtration and the filtrate was concentrated under reduced pressure. The residue was recrystallised with ethanol to give compound **2** (1.7 g, 95% yield) as light yellow crystal. ^1^H NMR (300 MHz, DMSO-d_6_*)* δ 12.48 (s, 1H, OH), 9.95 (s, 3H, NH_3_^+^), 8.00 (s, 1H, ArH), 6.77 (s, 1H, ArH), 3.94 (s, 3H, OCH_3_), 2.55 (s, 3H, CH_3_).

#### 2.2.2. General procedure for synthesis of compounds A1 − A14, B1 − B13, C1 − C17 and D1 − D4

Compound **2** (100 mg, 0.552 mmol, 1.0 eq) and *N, N*-diisopropylethylamine (273 μL, 1.656 mmol, 3.0 eq) were dissolved in DCM under ice-water bath. A solution of triphosgene (67 mg, 0.226 mmol, 0.41 eq) in DCM was added dropwise and the mixture was vigorously stirred at room temperature for 1 h. Further, the corresponding amines (1.2 eq) was added at room temperature. Upon completion of the reaction (as indicated by TLC in 3 ∼ 5 h), the solvent was removed by rotary evaporation. The residue was purified by flash chromatography on silica (0 − 50% ethyl acetate/petroleum ether) to give compounds **A1 **−** 14**, **B1 **−** 13**, **C1 **−** 7**, **D1**−**D4**, respectively.

*1–(5-acetyl-4-hydroxy-2-methoxyphenyl)-3-methylurea* **(A1)**. White solid, 52.98% yield, m. p.: 211–213 °C. ^1^H NMR (400 MHz, DMSO-d_6_) δ 12.46 (s, 1H), 8.52–8.47 (m, 1H), 7.82 (s, 1H), 6.62–6.53 (m, 2H), 3.90 (s, 3H), 2.64 (d, *J* = 4.6 Hz, 3H), 2.51 (s, 3H). ^13 ^C NMR (101 MHz, DMSO-d_6_) δ 203.54, 159.18, 156.51, 155.69, 122.30, 120.39, 112.51, 99.63, 56.75, 27.04, 26.47. HRMS (ESI): *m/z* [M + Na]^+^ calcd for C_11_H_14_N_2_O_4_: 261.0846; found: 261.0846.

*3–(5-acetyl-4-hydroxy-2-methoxyphenyl)-1,1-diethylurea* **(A2)**. White solid, 63.84% yield, m. p.: 132–134 °C. ^1^H NMR (400 MHz, DMSO-d_6_) δ 12.56 (s, 1H), 7.98 (s, 1H), 7.32 (s, 1H), 6.56 (s, 1H), 3.86 (s, 3H), 3.30 (q, *J* = 7.1 Hz, 4H), 2.53 (s, 3H), 1.10 (t, *J* = 7.1 Hz, 6H). ^13 ^C NMR (101 MHz, DMSO-d_6_) δ 203.45, 161.01, 159.12, 155.20, 126.45, 121.45, 112.63, 99.78, 56.82, 41.09 (2 C), 27.13, 14.27 (2 C). HRMS (ESI): *m/z* [M + H]^+^ calcd for C_14_H_20_N_2_O_4_: 281.1496; found: 281.1498.

*3–(5-acetyl-4-hydroxy-2-methoxyphenyl)-1,1-diisopropylurea* **(A3)**. White solid, 40.94% yield, m. p.: 148–149 °C. ^1^H NMR (400 MHz, DMSO-d_6_) δ 12.51 (s, 1H), 8.21 (s, 1H), 7.02 (s, 1H), 6.58 (s, 1H), 3.99–3.90 (m, 2H), 3.89 (s, 3H), 2.53 (s, 3H), 1.23 (d, *J* = 6.8 Hz, 12H). ^13 ^C NMR (101 MHz, DMSO-d_6_) δ 203.54, 160.19, 157.36, 154.53, 123.47, 121.90, 112.62, 99.75, 57.04, 45.23 (2 C), 27.20, 21.56 (4 C). HRMS (ESI): *m/z* [M + Na]^+^ calcd for C_16_H_24_N_2_O_4_: 331.1628; found: 331.1631.

*1–(5-acetyl-4-hydroxy-2-methoxyphenyl)-3-methoxyurea* **(A4)**. White solid, 66.68% yield, m. p.: 176–177 °C. ^1^H NMR (400 MHz, DMSO-d_6_) δ 12.52 (s, 1H), 9.67 (s, 1H), 8.26 (s, 1H), 7.99 (s, 1H), 6.61 (s, 1H), 3.90 (s, 3H), 3.64 (s, 3H), 2.53 (s, 3H). ^13 ^C NMR (101 MHz, DMSO-d_6_) δ 203.37, 160.67, 157.36, 157.24, 123.24, 119.97, 112.68, 99.99, 64.31, 56.97, 27.17. HRMS (ESI): *m/z* [M + Na]^+^ calcd for C_11_H_14_N_2_O_5_: 277.0795; found: 277.0797.

*1–(5-acetyl-4-hydroxy-2-methoxyphenyl)-3–(2,2,2-trifluoroethyl) urea* **(A5)**. White solid, 33.41% yield, m. p.: 232–234 °C. ^1^H NMR (400 MHz, DMSO-d_6_) δ 12.47 (s, 1H), 8.47 (d, *J* = 4.7 Hz, 1H), 8.12 (s, 1H), 7.31 (t, *J* = 6.4 Hz, 1H), 6.58 (s, 1H), 3.99–3.89 (m, 5H), 2.51 (s, 3H). ^13 ^C NMR (101 MHz, DMSO-d_6_) δ 203.50, 159.60, 155.81, 155.40, 129.54, 125.57 (d, *J* = 279.0 Hz), 120.77, 112.55, 99.79, 56.83, 40.46, 27.09. HRMS (ESI): *m/z* [M + Na]^+^ calcd for C_12_H_13_F_3_N_2_O_4_: 329.0720; found: 329.0718.

*1–(5-acetyl-4-hydroxy-2-methoxyphenyl)-3-ethylurea* **(A6)**. White solid, 33.41% yield, m. p.: 211–212 °C. ^1^H NMR (600 MHz, DMSO-d_6_) δ 12.44 (s, 1H), 8.51 (s, 1H), 7.76 (s, 1H), 6.69 (t, *J* = 4.7 Hz, 1H), 6.54 (s, 1H), 3.89 (s, 3H), 3.12–3.06 (m, 2H), 2.50 (s, 3H), 1.04 (t, *J* = 7.2 Hz, 3H). ^13 ^C NMR (151 MHz, DMSO-d_6_) δ 203.45, 159.09, 155.76, 155.58, 122.35, 120.22, 112.54, 99.58, 56.71, 34.32, 26.97, 15.83. HRMS (ESI): *m/z* [M + Na]^+^ calcd for C_12_H_16_N_2_O_4_: 275.1002; found: 275.1005.

*3–(5-acetyl-4-hydroxy-2-methoxyphenyl)-1,1-dimethylurea* **(A7)**. White solid, 70.43% yield, m. p.: 176–177 °C. ^1^H NMR (600 MHz, DMSO-d_6_) δ 12.55 (s, 1H), 7.96 (s, 1H), 7.46 (s, 1H), 6.56 (s, 1H), 3.85 (s, 3H), 2.90 (s, 6H), 2.52 (s, 3H). ^13 ^C NMR (151 MHz, DMSO-d_6_) δ 203.31, 161.03, 159.17, 156.50, 126.39, 121.45, 112.66, 99.80, 56.72, 56.70, 36.46, 27.03. HRMS (ESI): *m/z* [M + H]^+^ calcd for C_12_H_16_N_2_O_4_: 253.1183; found: 253.1185.

*1–(5-acetyl-4-hydroxy-2-methoxyphenyl)-3-(tert-butyl) urea* **(A8)**. White solid, 22.30% yield, m. p.: 241–242 °C. ^1^H NMR (600 MHz, DMSO-d_6_) δ 12.45 (s, 1H), 8.52 (s, 1H), 7.72 (s, 1H), 6.66 (s, 1H), 6.53 (s, 1H), 3.89 (s, 3H), 2.52 (s, 3H), 1.28 (s, 9H). ^13 ^C NMR (151 MHz, DMSO-d_6_) δ 203.51, 158.83, 155.30, 155.01, 122.60, 119.57, 112.51, 99.52, 56.70, 49.79, 29.53 (3 C), 27.09. HRMS (ESI): *m/z* [M + H]^+^ calcd for C_14_H_20_N_2_O_4_: 281.1496; found: 281.1495.

*1–(5-acetyl-4-hydroxy-2-methoxyphenyl)-3-cyclopropylurea* **(A9)**. White solid, 69.04% yield, m. p.: 191–192 °C. ^1^H NMR (600 MHz, DMSO-d_6_) δ 12.45 (s, 1H), 8.52 (s, 1H), 7.69 (s, 1H), 6.91 (s, 1H), 6.55 (s, 1H), 3.89 (s, 3H), 2.51 (m, 4H), 0.62 (m, 2H), 0.37 (m, 2H). ^13 ^C NMR (151 MHz, DMSO-d_6_) δ 203.46, 159.18, 156.51, 155.55, 122.11, 120.21, 112.54, 99.63, 56.78, 27.00, 22.74, 6.71 (2 C). HRMS (ESI): *m/z* [M + H]^+^ calcd for C_13_H_16_N_2_O_4_: 265.1183; found: 265.1183.

*N-(5-acetyl-4-hydroxy-2-methoxyphenyl)-4-methylpiperazine-1-carboxamide* (**A10**). White solid, 63.62% yield, m. p.: 141–142 °C. ^1^H NMR (600 MHz, DMSO-d_6_) δ 12.56 (s, 1H), 7.87 (s, 1H), 7.73 (s, 1H), 6.55 (s, 1H), 3.84 (s, 3H), 3.38 (d, *J* = 10.4 Hz, 4H), 2.52 (s, 3H), 2.29 (s, 4H), 2.19 (s, 3H). ^13 ^C NMR (151 MHz, DMSO-d_6_) δ 203.31, 161.35, 159.80, 156.03, 127.52, 121.10, 112.71, 99.88, 56.66, 54.86 (2 C), 46.18, 44.01 (2 C), 27.09. HRMS (ESI): *m/z* [M + H]^+^ calcd for C_15_H_21_N_3_O_4_: 308.1605; found: 308.1606.

*N-(5-acetyl-4-hydroxy-2-methoxyphenyl) morpholine-4-carboxamide* **(A11)**. White solid, 48.57% yield, m. p.: 220–221 °C. ^1^H NMR (600 MHz, DMSO-d_6_) δ 12.56 (s, 1H), 7.89 (s, 1H), 7.78 (s, 1H), 6.56 (s, 1H), 3.84 (s, 3H), 3.60 (s, 4H), 3.38 (d, *J* = 4.1 Hz, 4H), 2.53 (s, 3H). ^13 ^C NMR (151 MHz, DMSO-d_6_) δ 203.29, 161.42, 159.84, 156.25, 127.64, 120.93, 112.73, 99.92, 66.39 (2 C), 56.67, 44.53 (2 C), 27.10. HRMS (ESI): *m/z* [M + H]^+^ calcd for C_14_H_18_N_2_O_5_: 295.1288; found: 295.1290.

*N-(5-acetyl-4-hydroxy-2-methoxyphenyl) pyrrolidine-1-carboxamide* **(A12)**. White solid, 73.70% yield, m. p.: 118–120 °C. ^1^H NMR (400 MHz, DMSO-d_6_) δ 12.55 (s, 1H), 8.07 (s, 1H), 7.23 (s, 1H), 6.57 (s, 1H), 3.87 (s, 3H), 3.34 (t, *J* = 4.9 Hz, 4H), 2.52 (d, *J* = 2.8 Hz, 3H), 1.85 (t, *J* = 6.5 Hz, 4H). ^13 ^C NMR (101 MHz, DMSO-d_6_) δ 203.44, 160.86, 158.75, 154.60, 125.80, 121.31, 112.61, 99.79, 56.77, 45.91 (2 C), 27.10 (2 C), 25.53. HRMS (ESI): *m/z* [M + H]^+^ calcd for C_14_H_18_N_2_O_4_: 279.1339; found: 279.1339.

*N-(5-acetyl-4-hydroxy-2-methoxyphenyl)-4-ethylpiperazine-1-carboxamide* **(A13)**. White solid, 50.52% yield, m. p.: 125–126 °C. ^1^H NMR (400 MHz, DMSO-d_6_) δ 12.58 (s, 1H), 7.88 (s, 1H), 7.74 (s, 1H), 6.56 (s, 1H), 3.85 (s, 3H), 3.43–3.39 (m, 4H), 2.53 (s, 3H), 2.34 (dd, *J* = 12.3, 5.5 Hz, 6H), 1.02 (t, *J* = 7.2 Hz, 3H). ^13 ^C NMR (101 MHz, DMSO-d_6_) δ 203.41, 161.47, 159.99, 156.06, 127.84, 121.07, 112.68, 99.90, 56.68, 52.69, 52.09, 44.09, 27.16, 12.39. HRMS (ESI): *m/z* [M + Na]^+^ calcd for C_16_H_23_N_3_O_4_: 344.1581; found: 344.1584.

*N-(5-acetyl-4-hydroxy-2-methoxyphenyl) thiazolidine-3-carboxamide* **(A14)**. White solid, 66.47% yield, m. p.: 172–174 °C. ^1^H NMR (400 MHz, DMSO-d_6_) δ 12.58 (s, 1H), 7.87 (d, *J* = 4.6 Hz, 1H), 7.84 (s, 1H), 6.58 (s, 1H), 4.50 (s, 2H), 3.85 (s, 3H), 3.68 (t, *J* = 6.3 Hz, 2H), 3.04 (t, *J* = 6.3 Hz, 2H), 2.53 (s, 3H). ^13 ^C NMR (101 MHz, DMSO-d_6_) δ 203.38, 161.75, 160.14, 154.96, 128.30, 120.51, 112.72, 100.01, 56.71, 49.10, 48.82, 30.77, 27.17. HRMS (ESI): *m/z* [M + Na]^+^ calcd for C_13_H_16_N_2_O_4_S: 319.0723; found: 319.0727.

*1–(5-acetyl-4-hydroxy-2-methoxyphenyl)-3-phenylurea* **(B1)**. White solid, 25.22% yield, m. p.: 184–185 °C. ^1^H NMR (600 MHz, DMSO-d_6_) δ 12.49 (s, 1H), 9.19 (s, 1H), 8.56 (s, 1H), 8.14 (s, 1H), 7.45 (d, *J* = 7.7 Hz, 2H), 7.27 (t, *J* = 7.9 Hz, 2H), 6.96 (t, *J* = 7.3 Hz, 1H), 6.61 (s, 1H), 3.95 (s, 3H), 2.55 (s, 3H). ^13 ^C NMR (151 MHz, DMSO-d_6_) δ 203.44, 159.60, 155.87, 153.11, 140.20, 129.23(2 C), 122.18, 121.45, 120.84, 118.43 (2 C), 112.67, 99.84, 56.89, 27.12. HRMS (ESI): *m/z* [M + H]^+^ calcd for C_16_H_16_N_2_O_4_: 301.1183; found: 301.1184.

*1–(5-acetyl-4-hydroxy-2-methoxyphenyl)-3-(m-tolyl) urea* **(B2)**. White solid, 32.34% yield, m. p.: 209–210 °C. ^1^H NMR (600 MHz, DMSO-d_6_) δ 12.49 (s, 1H), 9.12 (s, 1H), 8.56 (s, 1H), 8.12 (s, 1H), 7.32 (s, 1H), 7.20 (d, *J* = 8.1 Hz, 1H), 7.15 (t, *J* = 7.7 Hz, 1H), 6.78 (d, *J* = 7.4 Hz, 1H), 6.61 (s, 1H), 3.95 (s, 3H), 2.55 (s, 3H), 2.28 (s, 3H). ^13 ^C NMR (151 MHz, DMSO-d_6_) δ 203.46, 159.56, 155.82, 153.07, 140.14, 138.42, 129.07, 122.93, 121.50, 120.72, 118.91, 115.61, 112.65, 99.82, 56.88, 27.12, 21.67. HRMS (ESI): *m/z* [M + H]^+^ calcd for C_17_H_18_N_2_O_4_: 315.1339; found: 315.1338.

*1–(5-acetyl-4-hydroxy-2-methoxyphenyl)-3-(o-tolyl) urea* **(B3)**. White solid, 15.10% yield, m. p.: 238–239 °C. ^1^H NMR (600 MHz, DMSO-d_6_) δ 12.48 (s, 1H), 8.56 (s, 1H), 8.55 (s, 1H), 8.41 (s, 1H), 7.81 (d, *J* = 8.0 Hz, 1H), 7.16 (d, *J* = 7.3 Hz, 1H), 7.13 (t, *J* = 7.6 Hz, 1H), 6.94 (t, *J* = 7.4 Hz, 1H), 6.61 (s, 1H), 3.96 (s, 3H), 2.53 (s, 3H), 2.25 (s, 3H). ^13 ^C NMR (151 MHz, DMSO-d_6_) δ 203.45, 159.53, 156.00, 153.45, 137.82, 130.59, 128.27, 126.47, 123.17, 122.05, 121.62, 121.11, 112.65, 99.82, 56.87, 27.07, 18.50. HRMS (ESI): *m/z* [M + H]^+^ calcd for C_17_H_18_N_2_O_4_: 315.1339; found: 315.1339.

*1–(5-acetyl-4-hydroxy-2-methoxyphenyl)-3-(p-tolyl) urea* **(B4)**. White solid, 52.57% yield, m. p.: 218–219 °C. ^1^H NMR (600 MHz, DMSO-d_6_) δ 12.49 (s, 1H), 9.09 (s, 1H), 8.55 (s, 1H), 8.09 (s, 1H), 7.34 (d, *J* = 8.3 Hz, 2H), 7.08 (d, *J* = 7.9 Hz, 2H), 6.60 (s, 1H), 3.94 (s, 3H), 2.54 (s, 3H), 2.24 (s, 3H). ^13 ^C NMR (151 MHz, DMSO-d_6_) δ 203.46, 159.53, 155.81, 153.14, 137.63, 130.98, 129.62 (2 C), 121.55, 120.71, 118.50 (2 C), 112.65, 99.80, 56.88, 27.09, 20.76. HRMS (ESI): *m/z* [M + H]^+^ calcd for C_17_H_18_N_2_O_4_: 315.1339; found: 315.1339.

*1–(5-acetyl-4-hydroxy-2-methoxyphenyl)-3–(3-fluorophenyl) urea* **(B5)**. White solid, 27.72% yield, m. p.: 217–218 °C. ^1^H NMR (600 MHz, DMSO-d_6_) δ 12.49 (s, 1H), 9.41 (s, 1H), 8.53 (s, 1H), 8.19 (s, 1H), 7.51 (d, *J* = 12.0 Hz, 1H), 7.30 (dd, *J* = 15.3, 7.9 Hz, 1H), 7.08 (d, *J* = 7.7 Hz, 1H), 6.77 (td, *J* = 8.5, 2.4 Hz, 1H), 6.62 (s, 1H), 3.95 (s, 3H), 2.55 (s, 3H). ^13 ^C NMR (151 MHz, DMSO-d_6_) δ 203.43, 163.69, 162.09, 159.78, 155.98, 152.94, 142.06 (d, *J* = 11.4 Hz), 130.78 (d, *J* = 9.9 Hz),121.10, 114.11, 112.69, 108.49 (d, *J* = 21.1 Hz), 105.07 (d, *J* = 26.5 Hz), 99.91, 56.92, 27.15. HRMS (ESI): *m/z* [M + H]^+^ calcd for C_16_H_15_FN_2_O_4_: 319.1089; found: 319.3030.

*1–(5-acetyl-2-methoxyphenyl)-3–(4-fluorophenyl) urea* **(B6)**. White solid, 16.45% yield, m. p.: 257–259 °C. ^1^H NMR (400 MHz, DMSO-d_6_) δ 12.50 (s, 1H), 9.23 (s, 1H), 8.54 (s, 1H), 8.12 (s, 1H), 7.49–7.43 (m, 2H), 7.15–7.08 (m, 2H), 6.61 (s, 1H), 3.94 (s, 3H), 2.54 (s, 3H). ^13 ^C NMR (101 MHz, DMSO-d_6_) δ 203.53, 159.64, 157.73 (d, *J* = 237.9 Hz), 155.85, 153.17, 136.54 (d, *J* = 2.4 Hz), 121.39, 120.79, 120.07 (d, *J* = 7.7 Hz, 2 C), 115.80 (d, *J* = 22.2 Hz, 2 C), 112.63, 99.86, 56.93, 27.18. HRMS (ESI): *m/z* [M + Na]^+^ calcd for C_16_H_15_FN_2_O_3_: 341.0908; found: 341.0904.

*1–(5-acetyl-2-methoxyphenyl)-3–(2-fluorophenyl) urea* **(B7)**. White solid, 17.36% yield, m. p.: 186–187 °C. ^1^H NMR (400 MHz, DMSO-d_6_) δ 12.50 (s, 1H), 9.13 (d, *J* = 1.6 Hz, 1H), 8.69 (s, 1H), 8.55 (s, 1H), 8.18 (td, *J* = 8.3, 1.4 Hz, 1H), 7.22 (ddd, *J* = 11.6, 8.2, 1.2 Hz, 1H), 7.12 (t, *J* = 7.7 Hz, 1H), 7.03–6.96 (m, 1H), 6.61 (s, 1H), 3.94 (s, 3H), 2.54 (d, *J* = 5.0 Hz, 3H). ^13 ^C NMR (101 MHz, DMSO-d_6_) δ 203.52, 159.76, 156.03, 152.97, 152.41(d, *J* = 242.4 Hz), 128.07 (d, *J* = 10.4 Hz), 124.93 (d, *J* = 3.7 Hz), 122.77 (d, *J* = 7.8 Hz), 121.17 (d, *J* = 17.3 Hz), 121.09 (2 C), 115.43 (d, *J* = 19.0 Hz), 112.61, 99.88, 56.90, 27.19. HRMS (ESI): *m/z* [M + Na]^+^ calcd for C_16_H_15_FN_2_O_3_: 341.0908; found: 341.0912.

*1–(5-acetyl-4-hydroxy-2-methoxyphenyl)-3–(3-methoxyphenyl) urea* **(B8)**. White solid, 32.20% yield, m. p.: 176–177 °C. ^1^H NMR (600 MHz, DMSO-d_6_) δ 12.49 (s, 1H), 9.21 (s, 1H), 8.54 (s, 1H), 8.12 (s, 1H), 7.19–7.15 (m, 2H), 6.94 (dd, *J* = 8.1, 0.7 Hz, 1H), 6.60 (s, 1H), 6.54 (dd, *J* = 8.2, 2.3 Hz, 1H), 3.94 (s, 3H), 3.73 (s, 3H), 2.55 (s, 3H). ^13 ^C NMR (151 MHz, DMSO-d_6_) δ 203.43, 160.18, 159.64, 155.89, 153.02, 141.43, 130.00, 121.37, 120.88, 112.67, 110.77, 107.61, 104.23, 99.83, 56.87, 55.39, 27.13. HRMS (ESI): *m/z* [M + Na]^+^ calcd for C_17_H_18_N_2_O_5_: 353.1108; found: 353.1105.

*1–(5-acetyl-4-hydroxy-2-methoxyphenyl)-3–(2-methoxyphenyl) urea* **(B9)**. White solid, 35.16% yield, m. p.: 228–229 °C. ^1^H NMR (600 MHz, DMSO-d_6_) δ 12.49 (s, 1H), 8.79 (d, *J* = 12.4 Hz, 2H), 8.53 (s, 1H), 8.11 (dd, *J* = 8.0, 1.6 Hz, 1H), 7.00 (dd, *J* = 8.1, 1.3 Hz, 1H), 6.94 (td, *J* = 7.8, 1.6 Hz, 1H), 6.88 (td, *J* = 7.8, 1.3 Hz, 1H), 6.59 (s, 1H), 3.94 (s, 3H), 3.86 (s, 3H), 2.54 (s, 3H). HRMS (ESI): *m/z* [M + H]^+^ calcd for C_17_H_18_N_2_O_5_: 331.1288; found: 331.1291.

*1–(5-acetyl-2-methoxyphenyl)-3–(4-methoxyphenyl) urea* **(B10)**. White solid, 27.75% yield, m. p.: 227–228 °C. ^1^H NMR (400 MHz, DMSO-d_6_) δ 12.49 (s, 1H), 9.02 (s, 1H), 8.55 (s, 1H), 8.05 (s, 1H), 7.38–7.31 (m, 2H), 6.90–6.82 (m, 2H), 6.60 (s, 1H), 3.94 (s, 3H), 3.71 (s, 3H), 2.54 (s, 3H). ^13 ^C NMR (101 MHz, DMSO-d_6_) δ 203.55, 159.49, 155.74, 154.83, 153.27, 133.26, 121.66, 120.52, 120.13 (2 C), 114.48 (2 C), 112.60, 99.80, 56.89, 55.61, 27.16. HRMS (ESI): *m/z* [M + Na]^+^ calcd for C_17_H_18_N_2_O_4_: 353.1108; found: 353.1108.

*1–(5-acetyl-4-hydroxy-2-methoxyphenyl)-3–(4-(trifluoromethyl) phenyl) urea* **(B11)**. White solid, 52.52% yield, m. p.: 242–243 °C. ^1^H NMR (600 MHz, DMSO-d_6_) δ 12.50 (s, 1H), 9.59 (s, 1H), 8.54 (s, 1H), 8.24 (s, 1H), 7.66 (d, *J* = 8.7 Hz, 2H), 7.62 (d, *J* = 8.8 Hz, 2H), 6.62 (s, 1H), 3.95 (s, 3H), 2.55 (s, 3H). ^13 ^C NMR (151 MHz, DMSO-d_6_) δ 203.39, 159.88, 156.00, 152.82, 143.90, 126.51 (d, *J* = 3.7 Hz,2C), 124.99 (d, *J* = 270.8 Hz), 122.18 (q, *J* = 31.8 Hz), 121.17, 120.98, 118.05 (2 C), 112.68, 99.90, 56.93, 27.11. HRMS (ESI): *m/z* [M + H]^+^ calcd for C_17_H_15_F_3_N_2_O_4_: 369.1057; found: 369.1054.

*1–(5-acetyl-4-hydroxy-2-methoxyphenyl)-3–(3-(trifluoromethyl) phenyl) urea* **(B12)**. White solid, 54.64% yield, m. p.: 228–229 °C. ^1^H NMR (600 MHz, DMSO-d_6_) δ 12.50 (s, 1H), 9.54 (s, 1H), 8.52 (s, 1H), 8.19 (s, 1H), 8.03 (s, 1H), 7.54–7.48 (m, 2H), 7.32–7.28 (m, 1H), 6.62 (s, 1H), 3.95 (s, 3H), 2.56 (s, 3H). ^13 ^C NMR (151 MHz, DMSO-d_6_) δ 203.43, 159.89, 156.05, 152.98 (d, *J* = 11.2 Hz), 141.05, 130.38, 130.06 (d, *J* = 31.3 Hz), 124.64 (d, *J* = 272.4 Hz), 121.93, 121.12 (d, *J* = 39.7 Hz), 118.42 (d, *J* = 3.8 Hz), 114.22 (d, *J* = 4.0 Hz), 112.70, 99.91, 79.59, 56.91, 27.18. HRMS (ESI): *m/z* [M + H]^+^ calcd for C_17_H_15_F_3_N_2_O_4_: 369.1057; found: 369.1054.

*1–(5-acetyl-4-hydroxy-2-methoxyphenyl)-3–(4-(trifluoromethoxy) phenyl) urea* **(B13)**. White solid, 55.81% yield, m. p.: 209–210 °C. ^1^H NMR (600 MHz, DMSO-d_6_) δ 12.49 (s, 1H), 9.39 (s, 1H), 8.54 (s, 1H), 8.16 (s, 1H), 7.57–7.53 (m, 2H), 7.27 (d, *J* = 8.6 Hz, 2H), 6.61 (s, 1H), 3.94 (s, 3H), 2.54 (s, 3H). ^13 ^C NMR (151 MHz, DMSO-d_6_) δ 203.39, 159.75, 155.92, 153.01, 143.00, 139.46, 122.11, 121.20, 120.99, 120.64 (q, *J* = 255.1 Hz), 119.56, 119.46, 112.66, 99.85, 79.59, 56.87, 27.07. HRMS (ESI): *m/z* [M + H]^+^ calcd for C_17_H_15_F_3_N_2_O_5_: 385.1006; found: 385.1003.

*1–(5-acetyl-4-hydroxy-2-methoxyphenyl)-3–(2-chlorobenzyl) urea* **(C1)**. White solid, 15.24% yield, m. p.: 221–222 °C. ^1^H NMR (600 MHz, DMSO-d_6_) δ 12.45 (s, 1H), 8.52 (s, 1H), 8.06 (s, 1H), 7.45 (dd, *J* = 7.8, 1.2 Hz, 1H), 7.41 (dd, *J* = 7.6, 1.5 Hz, 1H), 7.35 (td, *J* = 7.4, 1.2 Hz, 1H), 7.30 (td, *J* = 7.7, 1.7 Hz, 1H), 7.25 (t, *J* = 5.9 Hz, 1H), 6.56 (s, 1H), 4.37 (d, *J* = 5.9 Hz, 2H), 3.91 (s, 3H), 2.50 (s, 3H). ^13 ^C NMR (151 MHz, DMSO-d_6_) δ 203.44, 159.28, 155.78, 137.67, 132.56, 129.56, 129.47, 129.06, 127.67, 122.12, 120.36, 112.55, 99.68, 79.60, 56.76, 41.18, 27.02. HRMS (ESI): *m/z* [M + H]^+^ calcd for C_17_H_17_ClN_2_O_4_: 349.0950; found: 349.0947.

*1–(5-acetyl-4-hydroxy-2-methoxyphenyl)-3-benzylurea* **(C2)**. White solid, 58.28% yield, m. p.: 194–195 °C. ^1^H NMR (400 MHz, DMSO-d_6_) δ 12.48 (s, 1H), 8.54 (s, 1H), 7.97 (s, 1H), 7.38–7.24 (m, 6H), 6.57 (s, 1H), 4.30 (d, *J* = 5.8 Hz, 2H), 3.91 (s, 3H), 2.51 (d, *J* = 2.6 Hz, 3H). ^13 ^C NMR (101 MHz, DMSO-d_6_) δ 203.57, 159.23, 155.89, 155.59, 140.69, 128.82 (2 C), 127.60 (2 C), 127.24, 122.25, 120.19, 112.52, 99.67, 56.78, 43.19, 27.09. HRMS (ESI): *m/z* [M + Na]^+^ calcd for C_17_H_18_N_2_O_4_: 337.1159; found: 337.1158.

*1–(5-acetyl-2-methoxyphenyl)-3–(4-methylbenzyl) urea* **(C3)**. White solid, 49.78% yield, m. p.: 146–147 °C. ^1^H NMR (400 MHz, DMSO-d_6_) δ 12.47 (s, 1H), 8.54 (s, 1H), 7.94 (s, 1H), 7.21–7.12 (m, 5H), 6.56 (s, 1H), 4.25 (d, *J* = 5.7 Hz, 2H), 3.90 (s, 3H), 2.51 (s, 3H), 2.28 (s, 3H). ^13 ^C NMR (101 MHz, DMSO-d_6_) δ 203.56, 159.21, 155.86, 155.57, 137.60, 136.27, 129.35 (2 C), 127.60 (2 C), 122.28, 120.14, 112.52, 99.65, 54.18, 42.94, 26.49, 21.55. HRMS (ESI): *m/z* [M + Na]^+^ calcd for C_18_H_20_N_2_O_3_: 351.1315; found: 351.1318.

*1–(5-acetyl-2-methoxyphenyl)-3–(4-fluorobenzyl) urea* **(C4)**. White solid, 36.58% yield, m. p.: 241–242 °C. ^1^H NMR (400 MHz, DMSO-d_6_) δ 12.47 (s, 1H), 8.53 (s, 1H), 7.96 (s, 1H), 7.33 (dd, *J* = 8.5, 5.7 Hz, 2H), 7.22 (t, *J* = 5.9 Hz, 1H), 7.20–7.13 (m, 2H), 6.57 (s, 1H), 4.28 (d, *J* = 5.8 Hz, 2H), 3.90 (s, 3H), 2.51 (s, 3H). ^13 ^C NMR (101 MHz, DMSO-d_6_) δ 203.54, 161.61 (d, *J* = 242.0 Hz), 159.27, 155.88, 155.63, 136.95 (d, *J* = 3.0 Hz), 129.53 (d, *J* = 8.2 Hz, 2 C), 122.18, 120.28, 115.51 (d, *J* = 21.3 Hz, 2 C), 112.53, 99.67, 56.77, 42.45, 27.08. HRMS (ESI): *m/z* [M + Na]^+^ calcd for C_17_H_17_FN_2_O_3_: 355.1065; found: 355.1065.

*1–(5-acetyl-4-hydroxy-2-methoxyphenyl)-3–(4-methoxybenzyl) urea* **(C5)**. White solid, 32.83% yield, m. p.: 233–234 °C. ^1^H NMR (400 MHz, DMSO-d_6_) δ 12.47 (s, 1H), 8.54 (s, 1H), 7.92 (s, 1H), 7.24–7.20 (m, 2H), 7.13 (t, *J* = 5.8 Hz, 1H), 6.92–6.88 (m, 2H), 6.56 (s, 1H), 4.22 (d, *J* = 5.7 Hz, 2H), 3.90 (s, 3H), 3.73 (d, *J* = 2.8 Hz, 3H), 2.52 (s, 3H). ^13 ^C NMR (101 MHz, DMSO-d_6_) δ 203.57, 159.18, 158.67, 155.82, 155.56, 132.54, 128.97 (2 C), 122.28, 120.14, 114.20 (2 C), 112.52, 99.65, 56.78, 55.52, 42.66, 27.09. HRMS (ESI): *m/z* [M + Na]^+^ calcd for C_18_H_20_N_2_O_5_: 367.1264; found: 367.1260.

*1–(5-acetyl-4-hydroxy-2-methoxyphenyl)-3–(2-methoxybenzyl) urea* **(C6)**. White solid, 42.72% yield, m. p.: 237–239 °C. ^1^H NMR (400 MHz, DMSO-d_6_) δ 12.47 (s, 1H), 8.53 (s, 1H), 8.03 (s, 1H), 7.24 (td, *J* = 7.4, 1.3 Hz, 2H), 7.07 (t, *J* = 5.8 Hz, 1H), 7.00 (d, *J* = 7.6 Hz, 1H), 6.92 (td, *J* = 7.4, 0.9 Hz, 1H), 6.56 (s, 1H), 4.25 (d, *J* = 5.8 Hz, 2H), 3.90 (s, 3H), 3.83 (s, 3H), 2.51 (s, 3H). ^13 ^C NMR (101 MHz, DMSO-d_6_) δ 203.57, 159.15, 157.25, 155.85, 155.58, 128.63, 128.51, 128.02, 122.33, 120.63, 120.14, 112.50, 110.92, 99.64, 56.76, 55.78, 38.51, 27.07. HRMS (ESI): *m/z* [M + Na]^+^ calcd for C_18_H_20_N_2_O_5_: 367.1264; found: 367.1261.

*1–(5-acetyl-4-hydroxy-2-methoxyphenyl)-3-(furan-2-ylmethyl) urea* **(C7)**. White solid, 50.67% yield, m. p.: 231–232 °C. ^1^H NMR (400 MHz, DMSO-d_6_) δ 12.47 (s, 1H), 8.52 (s, 1H), 7.95 (s, 1H), 7.60 (d, *J* = 1.2 Hz, 1H), 7.15 (t, *J* = 5.6 Hz, 1H), 6.57 (s, 1H), 6.41 (dd, *J* = 3.1, 1.9 Hz, 1H), 6.27 (d, *J* = 3.1 Hz, 1H), 4.29 (d, *J* = 5.6 Hz, 2H), 3.90 (s, 3H), 2.52 (s, 3H). ^13 ^C NMR (101 MHz, DMSO-d_6_) δ 203.55, 159.27, 155.60, 155.58, 153.45, 142.61, 122.10, 120.25, 112.51, 110.92, 107.01, 99.67, 56.78, 36.50, 27.08. HRMS (ESI): *m/z* [M + Na]^+^ calcd for C_15_H_16_N_2_O_5_: 327.0951; found: 327.0955.

*1–(5-acetyl-4-hydroxy-2-methoxyphenyl)-3-phenethylurea* **(D1)**. White solid, 43.98% yield, m. p.: 164–165 °C. ^1^H NMR (400 MHz, DMSO-d_6_) δ 12.48 (s, 1H), 8.54 (s, 1H), 7.90 (s, 1H), 7.34–7.18 (m, 6H), 6.79 (t, *J* = 5.6 Hz, 1H), 6.54 (s, 1H), 3.89 (s, 3H), 3.35 (dd, *J* = 12.9, 6.9 Hz, 2H), 2.74 (t, *J* = 7.1 Hz, 2H), 2.52 (s, 3H). ^13 ^C NMR (101 MHz, DMSO-d_6_) δ 203.57, 159.13, 155.84, 155.56, 140.05, 129.17 (2 C), 128.83 (2 C), 126.54, 122.34, 120.14, 112.51, 99.63, 56.75, 41.04, 36.37, 27.08. HRMS (ESI): *m/z* [M + Na]^+^ calcd for C_18_H_20_N_2_O_4_: 351.1315; found: 351.1318.

*1–(5-acetyl-4-hydroxy-2-methoxyphenyl)-3–(4-fluorophenethyl) urea* **(D2)**. White solid, 44.41% yield, m. p.: 224–225 °C. ^1^H NMR (400 MHz, DMSO-d_6_) δ 12.46 (s, 1H), 8.53 (s, 1H), 7.87 (s, 1H), 7.30–7.25 (m, 2H), 7.17–7.10 (m, 2H), 6.76 (t, *J* = 5.6 Hz, 1H), 6.55 (s, 1H), 3.89 (s, 3H), 3.32 (dd, *J* = 12.9, 6.9 Hz, 2H), 2.73 (t, *J* = 7.1 Hz, 2H), 2.52 (s, 4H). ^13 ^C NMR (101 MHz, DMSO-d_6_) δ 203.56, 161.31 (d, *J* = 241.5 Hz), 159.14, 155.82, 155.56, 136.17 (d, *J* = 3.1 Hz), 130.93 (d, *J* = 7.8 Hz, 2 C), 122.30, 120.14, 115.47 (d, *J* = 21.0 Hz, 2 C), 112.51, 99.63, 56.75, 41.04, 35.45, 27.07. HRMS (ESI): *m/z* [M + Na]^+^ calcd for C_18_H_19_FN_2_O_4_: 369.1221; found: 369.1225.

*1–(5-acetyl-4-hydroxy-2-methoxyphenyl)-3–(4-methoxyphenethyl) urea* **(D3)**. White solid, 57.99% yield, m. p.: 180–181 °C. ^1^H NMR (400 MHz, DMSO-d_6_) δ 12.47 (s, 1H), 8.53 (s, 1H), 7.88 (s, 1H), 7.23–7.10 (m, 2H), 6.93–6.81 (m, 2H), 6.74 (t, *J* = 5.6 Hz, 1H), 6.55 (s, 1H), 3.89 (s, 3H), 3.72 (d, *J* = 3.7 Hz, 3H), 3.29 (dd, *J* = 12.9, 6.9 Hz, 2H), 2.67 (dd, *J* = 9.2, 5.0 Hz, 2H), 2.52 (d, *J* = 1.8 Hz, 3H). ^13 ^C NMR (101 MHz, DMSO-d_6_) δ 203.56, 159.12, 158.13, 155.83, 155.55, 131.85, 130.10 (2 C), 122.36, 120.10, 114.23 (2 C), 112.51, 99.62, 56.74, 55.42, 41.27, 35.46, 27.07. HRMS (ESI): *m/z* [M + Na]^+^ calcd for C_19_H_22_N_2_O_5_: 381.1421; found: 384.1424.

*1–(5-acetyl-4-hydroxy-2-methoxyphenyl)-3–(2-fluorophenethyl)urea* **(D4)**. White solid, 27.50% yield, m. p.: 204–205 °C. ^1^H NMR (600 MHz, DMSO-d_6_) δ 12.45 (s, 1H), 8.51 (s, 1H), 7.83 (s, 1H), 7.34–7.25 (m, 2H), 7.18–7.13 (m, 2H), 6.80 (t, *J* = 5.7 Hz, 1H), 6.54 (s, 1H), 3.89 (s, 3H), 3.36–3.33 (m, 2H), 2.78 (t, *J* = 7.1 Hz, 2H), 2.51 (s, 3H). ^13 ^C NMR (151 MHz, DMSO-d_6_) δ 203.46, 161.99, 160.38, 159.17, 155.72 (d, *J* = 29.4 Hz), 131.62 (d, *J* = 5.0 Hz), 128.64 (d, *J* = 8.1 Hz), 126.56 (d, *J* = 15.9 Hz), 124.79 (d, *J* = 3.4 Hz), 122.24, 120.32, 115.54 (d, *J* = 22.0 Hz), 112.54, 99.61, 56.71, 29.68, 27.00, 18.97. HRMS (ESI): *m/z* [M + Na]^+^ calcd for C_19_H_22_N_2_O_5_: 369.1221; found: 369.1221.

### 2.3. Biological assays

#### 2.3.1. Cell culture

Mouse peritoneal macrophages obtained from BeNa Culture Collection Company (Beijing, China). The human skin keratinocyte HaCaT cell line obtained from American Type Culture Collection (ATCC, Manassas, VA). HaCaT cells and RAW264.7 cells were cultured in DMEM (Hyclone, Logan, UT) supplemented with 10% foetal bovine serum (Biological Industries, Israel). These cells were cultured in a 37 °C, 5% CO_2_ incubator. The cells grew until they converged to 70 − 80% before treatment.

#### 2.3.2. Determination of NO

RAW264.7 cells (7 × 10^4^ cells/well) were seeded into 48-well plate and used for experiments after 24 h. RAW264.7 cells were pre-treated with compounds (10 µM) for 1 h, co-treated with LPS (0.5 mg/mL) for 24 h. After 24 h collect cell supernatant, NO production was measured using Griess Reagent assay (Beyotime). Griess reagent were mixed at a ratio of 1:1, and reacted at room temperature for 15 min. Measurement of absorbance at 450 nm with enzyme reader. At least three independent experiments were performed for the determination of IC_50_ values, which were shown as mean ± SD. All experimental data were analysed by using GraphPad Prism software, version 8.0 (GraphPad Inc.).

#### 2.3.3. Cell viability assay (MTT)

RAW264.7 cells (1 × 10^4^ cells/well) were seeded into 96-well plate and used for experiments after 24 h. Compounds (4 µM, 20 µM and 100 µM) treated cell for 24 h. Forty microliters of MTT solution (5 mg/mL, Sigma-Aldrich) was then added and incubated for an additional 4 h. After 4 h, cell culture supernatants were removed and DMSO (150 µL) was added into per well for dissolving the resulting crystals. Shaking about 1 0 ∼ 15 min and the absorbance at 492 nm was measured by a microplate reader (MQX200, Bio-Tek, Winooski, VT). At least three independent experiments were performed for the determination of IC_50_ values, which were shown as mean ± SD. All experimental data were analysed by using GraphPad Prism software, version 8.0 (GraphPad Inc.).

#### 2.3.4. Hyperproliferation assay

HaCaT cells were seeded in 96-well plates and cultured at 37 °C in a humidified 5% CO_2_ atmosphere. When the cell confluency reached 60–70%, HaCaT cells were then starved in serum-free DMEM for 12 h before stimulated by 100 ng/mL LPS for 24 h to mimic hyperproliferative psoriatic keratinocytes. The cell viability was measured by MTT method. Results are expressed as the percentage survival of cells in the cell treated group and the untreated control group (without the tested compound and LPS).

#### 2.3.5. Western blotting

RAW264.7 cells were seeded into 6-well plate with 2 × 10^6^ cells per well and maintained about 24 h, and then pre-treated with compound **B12** (2.5, 5, 10 µM) for 1 h, co-treated with LPS (0.5 µg/mL) for 0.5 h or 24 h. Western blotting assay was performed as the method described previously^24^. Briefly, the cells were lysed in 300 ml RIPA cell lysis buffer (Contains PMSF and phosphatase inhibitors, Beyotime China) and incubated on ice for 30 min. Collecting supernatant by centrifugation. The same amount of protein was separated by SDS-PAGE and transferred to PVDF membrane (GE Healthcare, Amersham, UK). The blotted membrane incubated with the primary antibodies and allowed to react for an additional 16 h at 4 °C. All antibodies obtain from Cell Signalling Technology, Boston, MA. The membranes incubated with a 1:5000 dilutions of HRP-conjugated secondary antibody (Beyotime Biotech, Nantong, China) for 1 h at room temperature. Signals were visualised using ECL system (Thermo Fisher Scientific, Waltham, MA).

HaCaT cells were seeded into 6-well plate with 2 × 10^6^ cells per well. After 24 h culture and 12 h starvation, they were stimulated with SUV irradiation. The cells were irritated with 40 KJ/m^2^ doses of SUV at 30 min. In order to study the effect of compound **B12**, the cells were starved in serum-free medium for 12 h, and then pre-treated with compound **B12** (2.5, 5, 10 μM) for 6 h before SUV irradiation. Follow the same steps as above to transfer the electrophoretic band to PVDF membrane. All steps were performed in the same way as above except that the types of primary and secondary antibodies were different.

#### 2.3.6. Animal study

Inbred 6–8-week-old female BALB/c mice were obtained from Animal Department of Anhui Medical University (China). All mice were allowed to acclimatise in the specific pathogen free (SPF) conditions for 1 week before any experiments were started. Mice were raised in a 12 h light/dark cycle with humidity (55 ± 5%) and temperature (22 ± 1 °C). All animal protocols were approved by the Ethics Committee in Animal Experimentation at Anhui Medical University (Hefei, China) following the guidelines for Care and Use of Laboratory Animals. To induce the experimental psoriatic skin inflammation, mice were randomly divided into 5 groups (*n* = 6): Normal, Vehicle (Imiquimod (IMQ), Sichuan Mingxin Pharmaceutical, Sichuan, China applied only), Dexamethasone treatment (IMQ with 20 mg/kg Dex), Compound **B12** L (IMQ with 20 mg/kg Compound **B12**), and Compound **B12** H (IMQ with 40 mg/kg Compound **B12**). The psoriatic skin inflammation was established by topically applying 62.5 mg of IMQ cream on the shaved 2 cm × 3 cm back skins. All rats were given intragastric administration once a day. During treatment for 7 days, to score the severity of inflammation of mouse skin, an objective scoring system was developed based on the clinical psoriasis area and severity index. Erythema, scaling, and thickening were scored independently from 0 to 4 as follows: 0, none; 1, slight; 2, moderate; 3, marked; 4, very marked and weight loss. The cumulative score (erythema plus scaling plus thickening) served to indicate the severity of inflammation (scale 0 ∼ 12). On the 8th day, the mice were taken eyeball blood and back skin. One-half of the samples were immediately fixed in 4% paraformaldehyde and for haematoxylin and eosin (H&E) staining. The other samples were put in a −80 °C freezer. Before used, they were placed at room temperature for 30 min. After that, they were added 1 × PBS proportionally, homogenised and centrifuged. The supernatant was collected and used for ELISA assay and Western blot assay. All animal studies were conducted according to the guidelines approved by the Laboratory Animal Centre of Anhui University. The histological image was obtained using 3DHISTECH’s Slide Converter (3DHISTECH, Hungary).

#### 2.3.7. Human liver microsomes stability assay in vitro

The livers were homogenised initially in a Tris (0.1 M)/KCl (0.1 M)/EDTA (1 mM)/butylated sodium hydroxytoluene (BHT) (0.02 mM) buffer. Add 1.5 µL control/test compound **B12** working solution to 238.5 µL liver microsome working solution, gently mix. Pre-incubate at 37 °C for 5 min. Start the reaction by adding 60 µL NADPH working solution. Mix by pipetting up and down. At each time point: 0, 5, 15, 30 and 60 min, remove 30 µL of reaction mixture to 300 µL of quenching solution. Vortex vigorously for ∼1 min and centrifuge at 4000 rpm at 4 °C for 15 min. Remove 100 µL of the supernatant and mixed with 100 µL distilled water for LC-MS/MS analysis.

#### 2.3.8. Statistical analysis

Data were expressed as the mean ± SEM. Statistical significance was assessed by one-way ANOVA and Turkish test, and differences between the two groups were examined by SPSS (version 14.0; SPNN Inc., Chicago, IL). *p* < 0.05 was considered to be statistically significant. All experiments dates were repeated at least three times.

## 3 Results and discussion

### 3.1. Chemistry

**Scheme 1.**
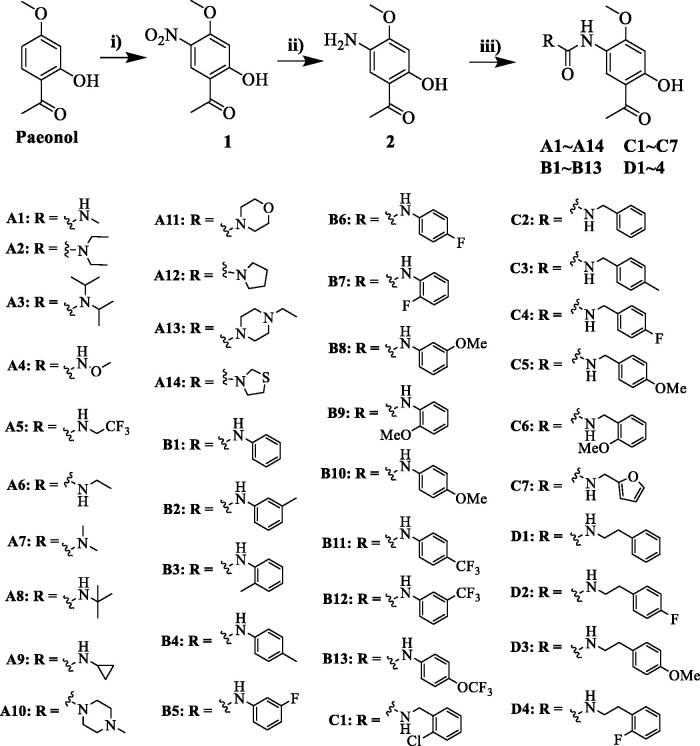
Synthesis of compounds **A1**−**A14**, **B1**−**B13**, **C1**−**C7** and **D1**−**D4**. Reagents and conditions: (i) HNO_3_, CH_3_COOH, rt to 0 °C, 3h; (ii) H_2_, Pd/C, methanol, rt, overnight; (iii) amines, triphosgene, *N, N*-diisopropylethylamine, DCM, rt, 3 h.

Compounds **A1**−**A14**, **B1**−**B13**, **C1**−**C7** and **D1**−**D4** were synthesised from paeonol as described in Scheme 1. First, nitration was achieved by treatment of paeonol with nitric acid in acetic acid, giving the nitro paeonol **1** at a 57% yield^25^. Subsequently, a selective reduction of **1** was performed by hydrogenation under 1 bar of hydrogen pressure using Pd/C to provide amino **2** at a 95% yield. Then 5-Amino-paeonol **2** was reacted with triphosgene in DCM to give crude 5-isocyanato-paeonol, directly reacted with various amines to give the desired compounds **A1 **−** 14**, **B1 **−** 13**, **C1 **−** 7**, and **D1 **−** 4**. Following the procedure, compounds **A1**−**A14** substituted with alkyl amines were obtained in moderate yields; compounds **B1**−**B13** substituted with aniline derivatives were produced in yields of 15 − 55%. The detailed synthesis procedures are described in the experimental section. The chemical structures of the compounds were confirmed by ^1^H NMR, ^13 ^C NMR and HRMS.

### 3.2. Inhibiting of NO release

**Figure 2.**
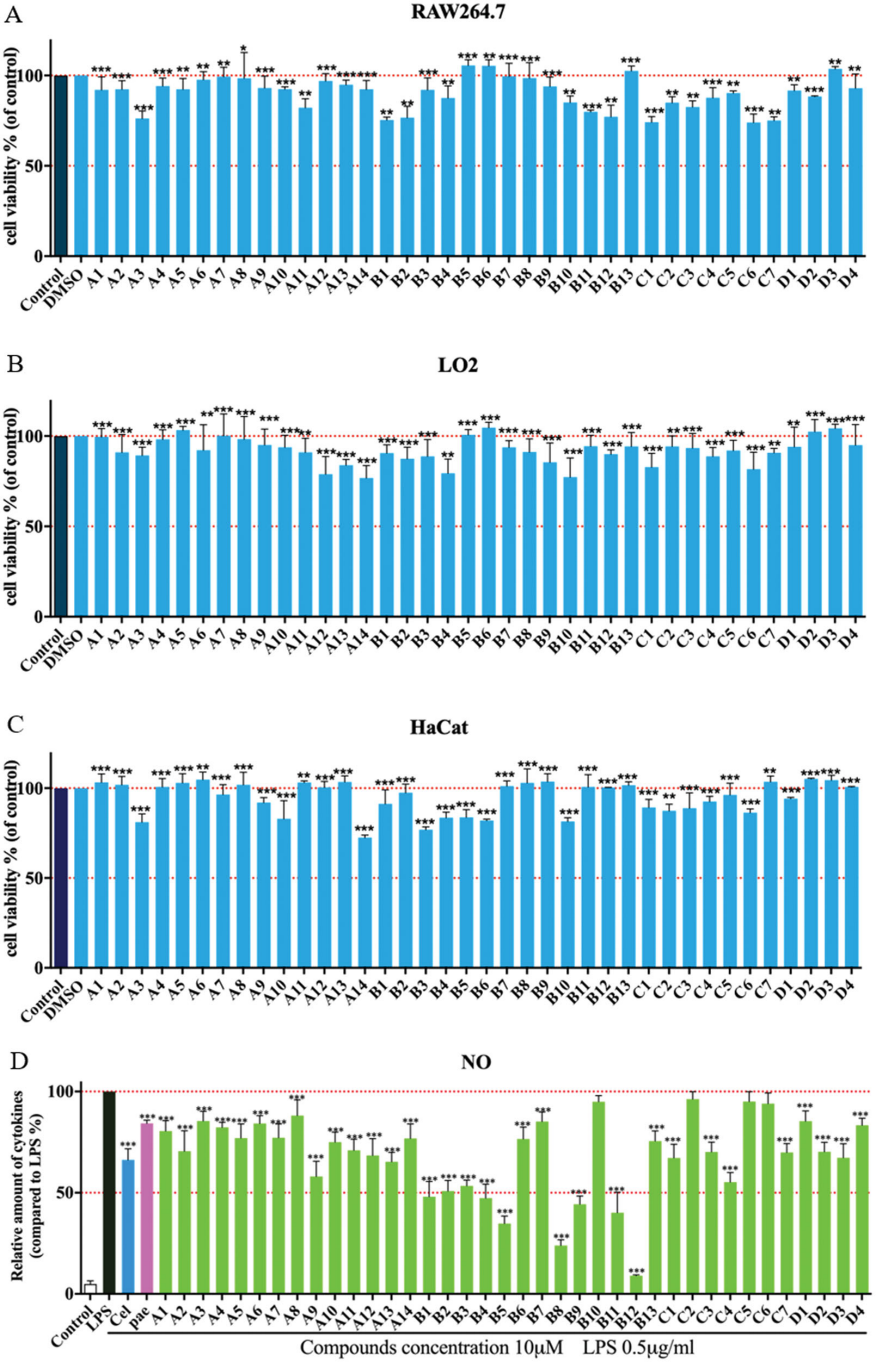
Cytotoxic evaluation of all compounds in RAW264.7 cells **(A)**, LO2 **(B)** and HaCaT cells **(C)**. Inhibition of NO production by in RAW264.7 cells **(D)**. Cell viability of all compounds were determined by MTT at 100 μM for 24 h; RAW264.7 cells were pre-treated with compounds (10 μM) for 1 h, incubated with LPS (0.5 μg/mL) for 24 h, NO production was measured using Griess Reagent assay. Paeonol (10 μM) and celecoxib (10 μM) are positive compound. The results are expressed as mean ± SD of three experiments. The concentration of DMSO is 1 0^−8 ^M. ***p < 0.001, **p < 0.01, *p < 0.05 vs LPS group.

Before determining the anti-inflammatory activity of the title compounds, cytotoxic activities were evaluated by an MTT assay. As shown in Figure 2(A–C), none of compounds showed significant intrinsic cytotoxicity against RAW264.7, LO2 or HaCat cells at 100 µM. Excessive NO production plays an important role in many inflammatory diseases. Subsequently, the anti-inflammatory activity of compounds was determined in LPS-stimulated RAW264.7 cells according to NO release^24^^,^^26^^,^^27^. As shown in Figure 2(D), most of compounds reduced LPS-induced NO secretion at 10 µM. And some compounds exhibited stronger inhibitory activity compared than celecoxib and paeonol. Compounds **A1**−**A8** showed weak potencies as well as paeonol, while compounds **A9**−**A14** showed a slight increase in potency. Based on this structural optimisation, it was found that alkyl or heterocyclic/cyclic amine-modified paeonol showed unsatisfactory results.

To improve anti-inflammatory activity and clearly explore structure-activity relationships, phenylurea, benzylurea and phenethylurea were also introduced at the 5-positon of paeonol. Compounds **B1**, **C2**, and **D1**, substituted with aniline, benzylamine, and phenethylamine, respectively, were synthesised and dramatically different inhibitory activities were observed (IR = 51%, 7.86% and 15.26%, respectively). Aniline-substituted **B1** proved to be a privileged scaffold. Subsequently, a range of *N*-phenyl groups was introduced. *Ortho*-substituted *N*-phenyl compounds **B3**, **B7** and **B9** showed weak to moderate inhibitory activity. *Para*-substituted *N*-phenyl compounds **B4**, **B6** and **B10** showed moderate inhibitory activity. However, compounds **B5**, **B8** and **B12**, having substituents at the *meta*-position of N-phenyl, showed dramatically improved activities (IR = 62.98%, 74.67%, and 95.46%, respectively). Among them, compound **B12** gave the best results, with an IC_50_ against the NO production was 2.14 μM, which may be due to lipid solubility and strong electron-withdrawing properties of the trifluoromethyl moiety^28^. Therefore, compound **B12** was selected as the lead compound for further evaluation (Table 1).

**Table 1.** Inhibitory effects of compounds **A1**−**A14**, **B1**−**B13**, **C1**−**C7** and **D1**−**D4** against NO production and cytotoxicity against RAW264.7 and LO2 cells^*^a^*^

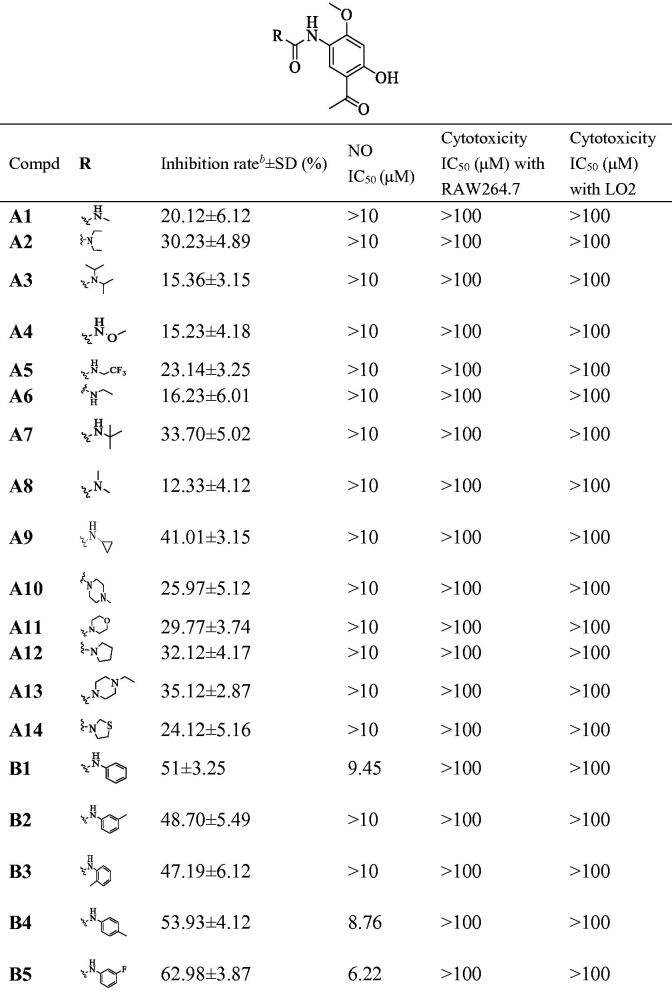	
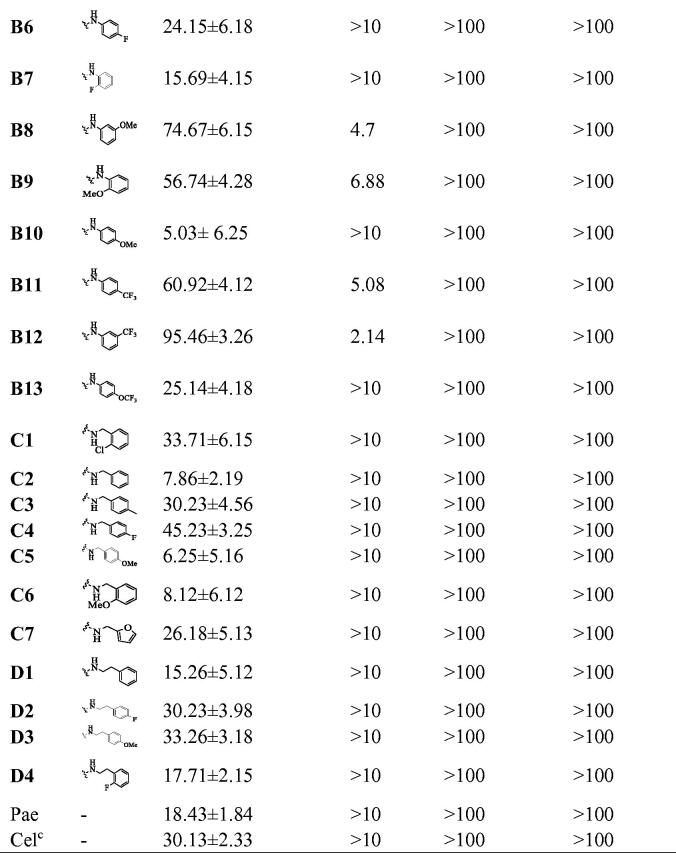	

^a^The cells were treated with compounds for 1 h and stimulated with LPS (0.5 μg/mL) for 24 h. The NO level in the culture medium were measured by nitrite and nitrate assay. All compounds showed no toxicities on RAW 264.7 cells at 20 μM (*p* > 0.05). All the data are displayed at least three independent experiments.

^b^The test concentration of compounds was 10 μM.

^c^Cel: Celecoxib as positive control.

### 3.3. Admet prediction

The absorption, distribution, metabolism, excretion, and toxicity (ADMET) of all compounds were predicted using the Discovery Studio 2018 (DS 2018) software^29^^,^^30^. The results are depicted in the 2 D graphs of ADMET PSA 2 D and ADMET AlogP98. Compound **B12** exhibited suitable solubility at 25 °C (solubility level of 2). All compounds were within the 95% and 99% confidence intervals of blood–brain barrier (BBB) penetration and had suitable HIA (absorption level of 0). Moreover, compound **B12** was likely to bind to plasma proteins (PPB# prediction was true). The results showed that compound **B12** was a non-cytochrome P450 2D6 inhibitor (CYP2D6# prediction was false). The drugability and safety of the compounds were initially evaluated using the ADMET predictions (Supplementary Information). Anti-inflammatory activity of compound **B12** was studied in the following steps.

### 3.4. Suppression LPS-induced iNOS and COX-2 activation

**Figure 3.**
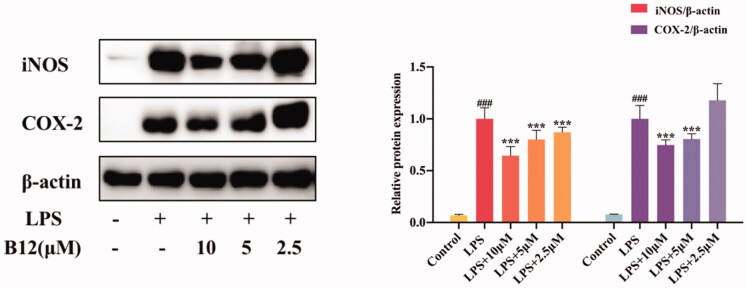
Inhibition of compound **B12** on expression of pro-inflammatory mediators in LPS-stimulated RAW264.7 cells. RAW264.7 cells were pre-treated with compound **B12** at 2.5, 5, 10 µM for 1 h, incubated with LPS (0.5 mg/mL) for 24 h. iNOS, COX-2 and β-actin were detected by Western blot. ^###^*p* < 0.001 compared with Control, ***p < 0.001 compared with LPS-stimulated cells; The blots are shown examples of three separate experiments.

Compound **B12** was selected as the title compound for the comprehensive consideration of potency, toxicity and drug-like properties. Notably, compound **B12** showed the highest potency activity against NO production (IC_50_ = 2.14 µM) and lower cytotoxicity (IC_50_ > 100 µM) in RAW264.7 cells. Therefore, inhibition of compound **B12** on the pro-inflammatory mediators iNOS and COX-2 was analysed by western blotting. As shown in Figure 3, the expression of iNOS and COX-2 was inhibited by compound **B12** in a dose-dependent manner. Thus, compound **B12** could prevent LPS-induced expression of inflammatory mediators.

### 3.5. Effect of compound B12 on LPS-induced HaCaT hyperproliferation

**Figure 4.**
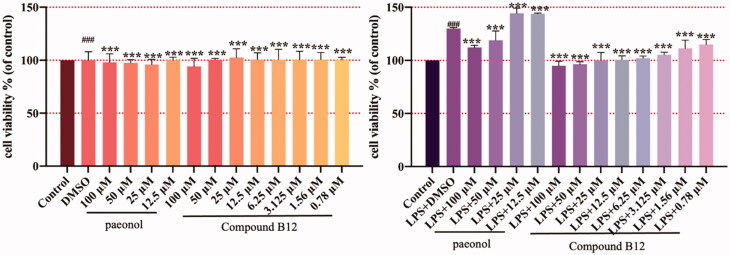
Compound **B12** inhibit hyperproliferation in LPS-stimulated HaCaT cells. HaCaT cells were pre-treated with compound **B12** at concentrations gradient for 6 h, incubated with LPS (100 g/mL) for 24 h. Cell viability were detected by MTT. ^###^*p* < 0.001 compared with Control, ***p < 0.001 compared with LPS-stimulated cells; The cell viability was shown examples of three separate experiments.

The changes in the cell function of keratinocytes are closely related to the occurrence and development of dermatitis. HaCaT cells are immortalised human keratinocytes with normal mutations, which is similar to primary human epidermal keratinocytes (KCs)^31^. The LPS-induced keratinocyte line (HaCaT cell) hyperproliferation model was used to simulate the hyperproliferation of dermatitis keratinocyte epidermis in this study. Therefore, title compounds against HaCaT cells hyperproliferation were tested, with results showing that compound **B12** inhibited excessive proliferation of LPS-induced HaCaT cells in a dose dependent manner, while paeonol did not have this effect (Figure 4).

### 3.6. Interaction of compound B12 and TOPK through CETSA

**Figure 5.**
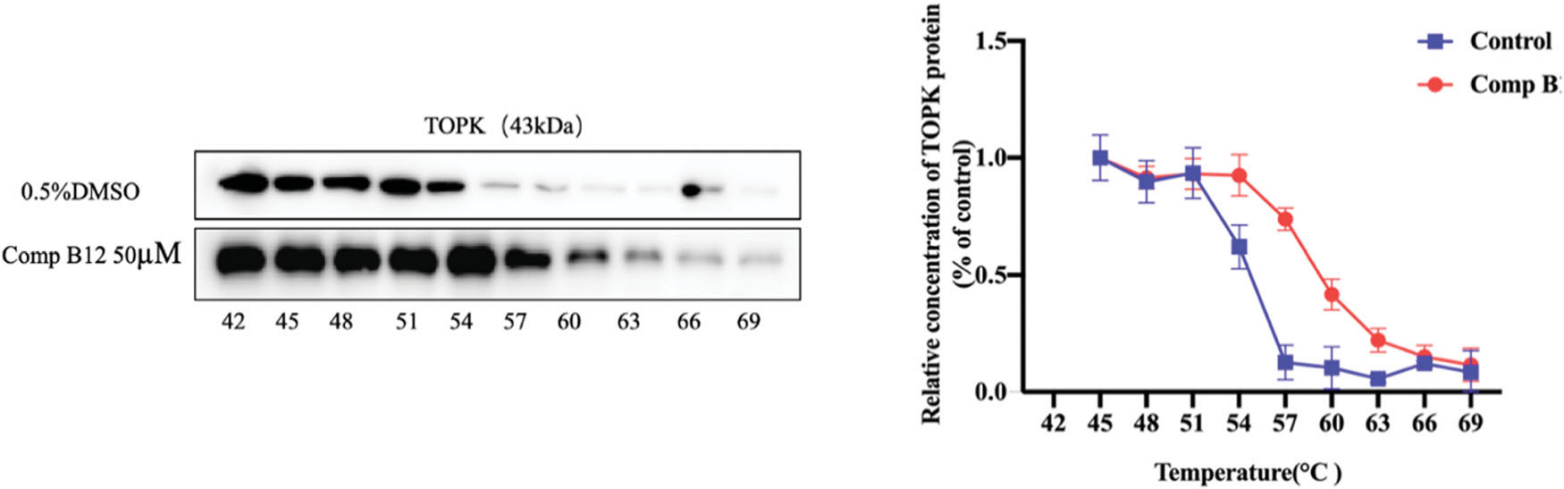
CETSA detect thermal stability of TOPK in presence of compound **B12**.

Based on the ligand-induced thermal stabilisation of target proteins, the cellular thermal shift assay **(**CETSA) assay is likely to validate drug binding, off-target effects or drug resistance in cell lines. To further detect the direct binding of compound **B12** with TOPK protein, CETSA was used in this study^32^. As shown in Figure 5, TOPK could be stabilised in presence of compound **B12,** especially at 57 °C, and noticeable results could be observed, which indicating that compound **B12** should be bound to TOPK to avoid its degradation.

### 3.7. Compound B12 suppressed LPS-induced TOPK/NF-κB/p38/JNK activation

**Figure 6.**
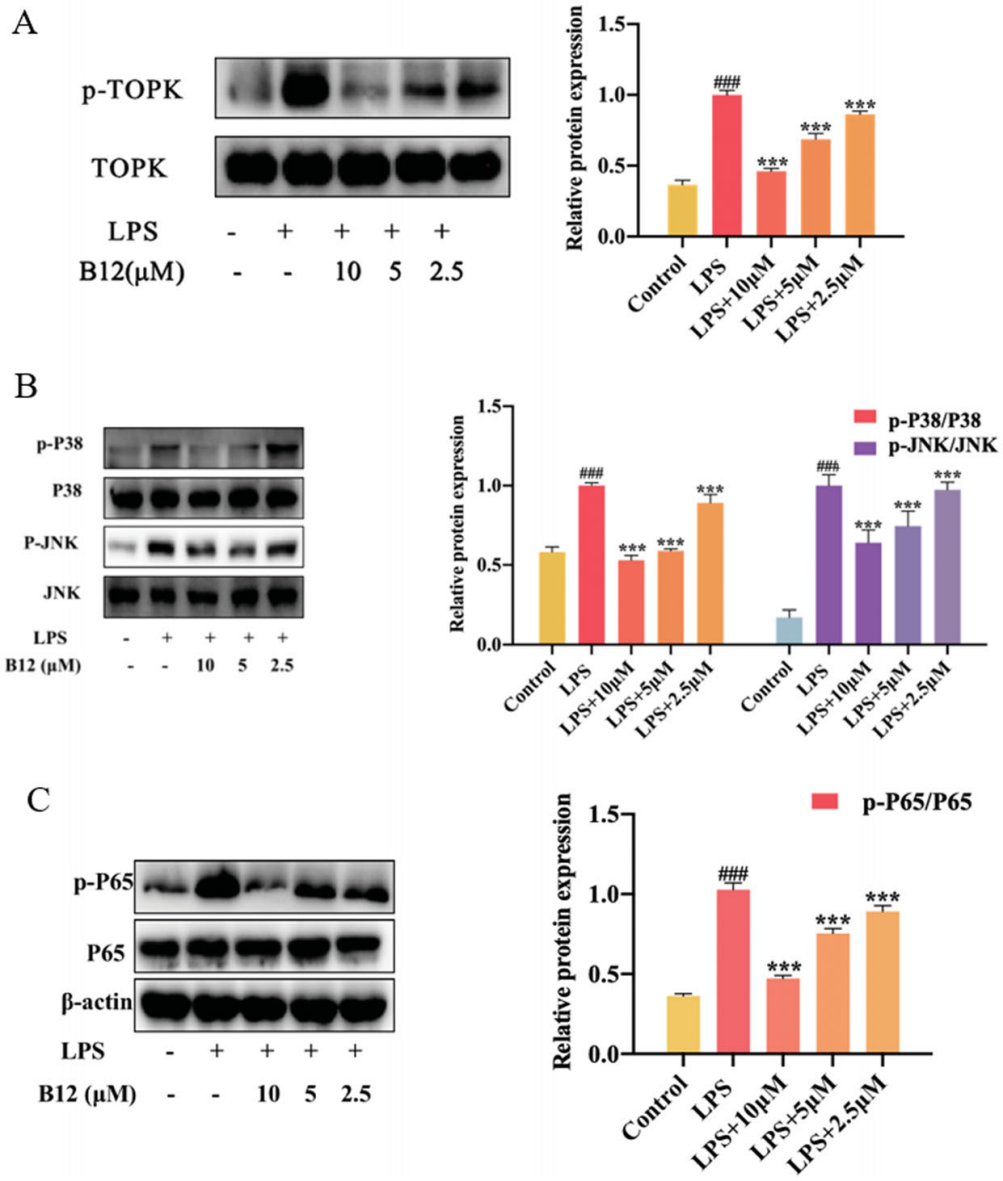
Compound **B12** suppressed LPS-induced MAPK and NF-κB activation. **(A)** Compound **B12** inhibited phosphorylation of TOPK. **(B)** Compound **B12** inhibited phosphorylation of JNΚ and p38. **(C)** Compound **B12** inhibited phosphorylation p65. Cells were treated with LPS (0.5 μg/mL) for 30 min. The results were showed as means ± SD (*n* = 3). ^###^*p* < 0.001 compared with LPS unstimulated cells; ***p < 0.001 compare with LPS-stimulated cells.

In RAW264.7 cell, TOPK is activated by LPS and activates the downstream protein pathways MAPK and NF-κ^33^^,^^34^. Some of inflammatory cytokines, such as LPS, IL-6 and TNF-α can activate IκB kinase (IKK), allowing the liberated NF-κB to translocate into the nucleus and bind to target promoters to turn on transcriptions. These results indicated that compound **B12** could affect TOPK phosphorylation (Figure 6(A)). Meanwhile, compound **B12** inhibited P38/JNK protein phosphorylation and NF-κB p65 translocated into the nucleus (Figure 6(B,C)). Therefore, we believe that compound **B12** may affect the phosphorylation of TOPK by targeting TOPK, and the conduction of the cell signal NF-κB/MAPK *in vitro*.

### 3.8. Down-regulated SUV-induced TOPK signalling pathway

**Figure 7.**
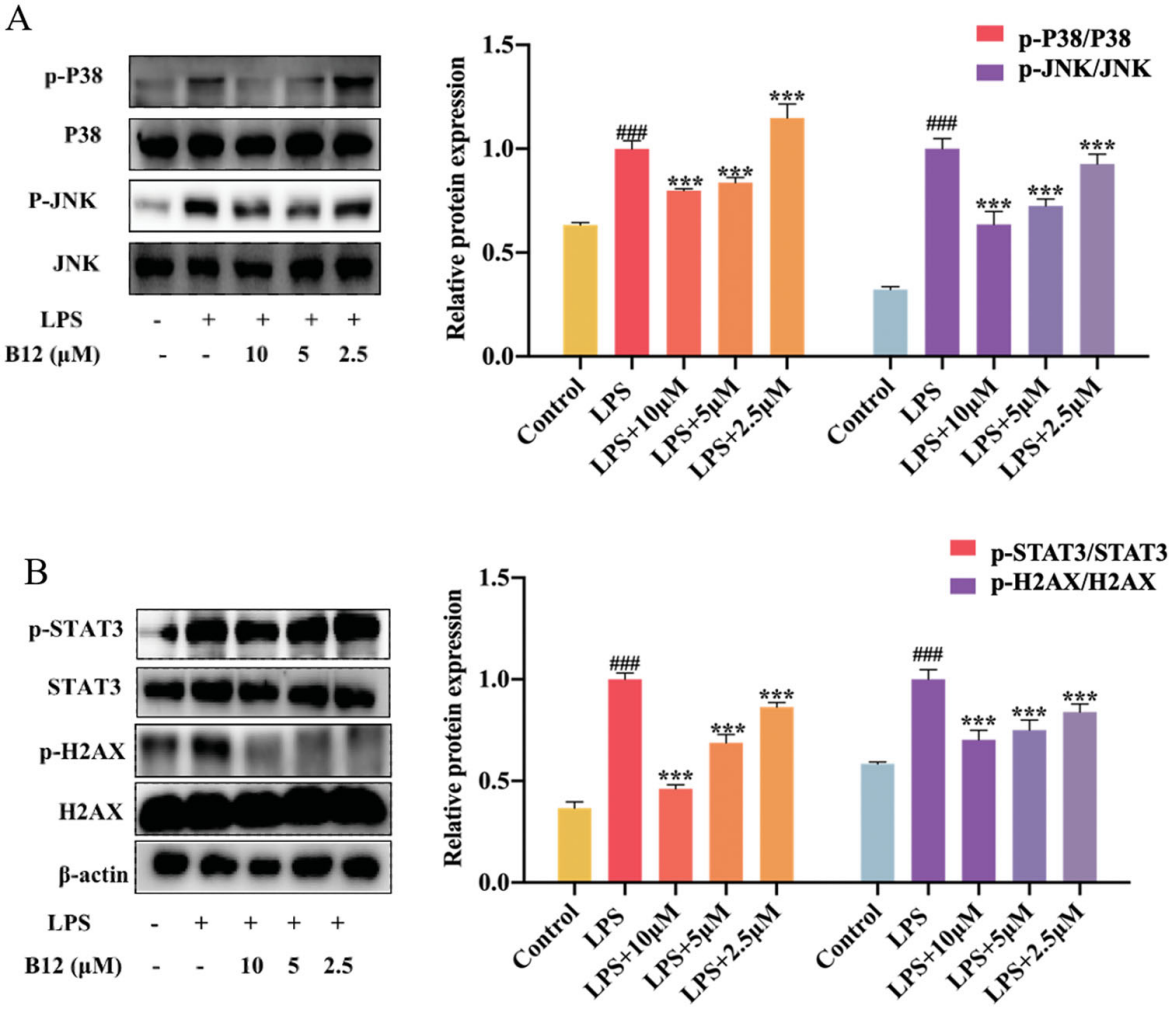
Compound **B12** dose-dependently suppressed SUV-induced MAPK and downstream proteins activation. **(A)** Compound **B12** inhibited phosphorylation of JNΚ and p38. **(B)** Compound **B12** inhibited phosphorylation of STAT3 and H2AX. Cells were treated with SUV (40 kJ/m^2^) for 30 min. The results were showed as means ± SD (*n* = 3) of at least three independent experiments. ^###^*p* < 0.001 compared with LPS unstimulated cells; ***p < 0.001 compare with LPS-stimulated cells.

Similarly, TOPK can be activated by the SUV. Histone H2AX is a substrate of TOPK and can be phosphorylated at Ser-139 (γ-H2AX) by TOPK^35^. TOPK is also an upstream kinase of p38. And STAT3 is a downstream kinase of p38. The levels of phosphorylated γ-H2AX and STAT3 gradually decreased after pre-treatment with compound **B12** from 2.5 μM to 10 μM for 6 h in HaCaT cells before 40 KJ/m^2^ SUV irradiation (Figure 7(A,B)). Results showed that compound **B12** could inhibit SUV-induced DNA damage through the regulation of TOPK.

### 3.9. Anti-inflammatory activity of compound B12 in vivo

**Figure 8.**
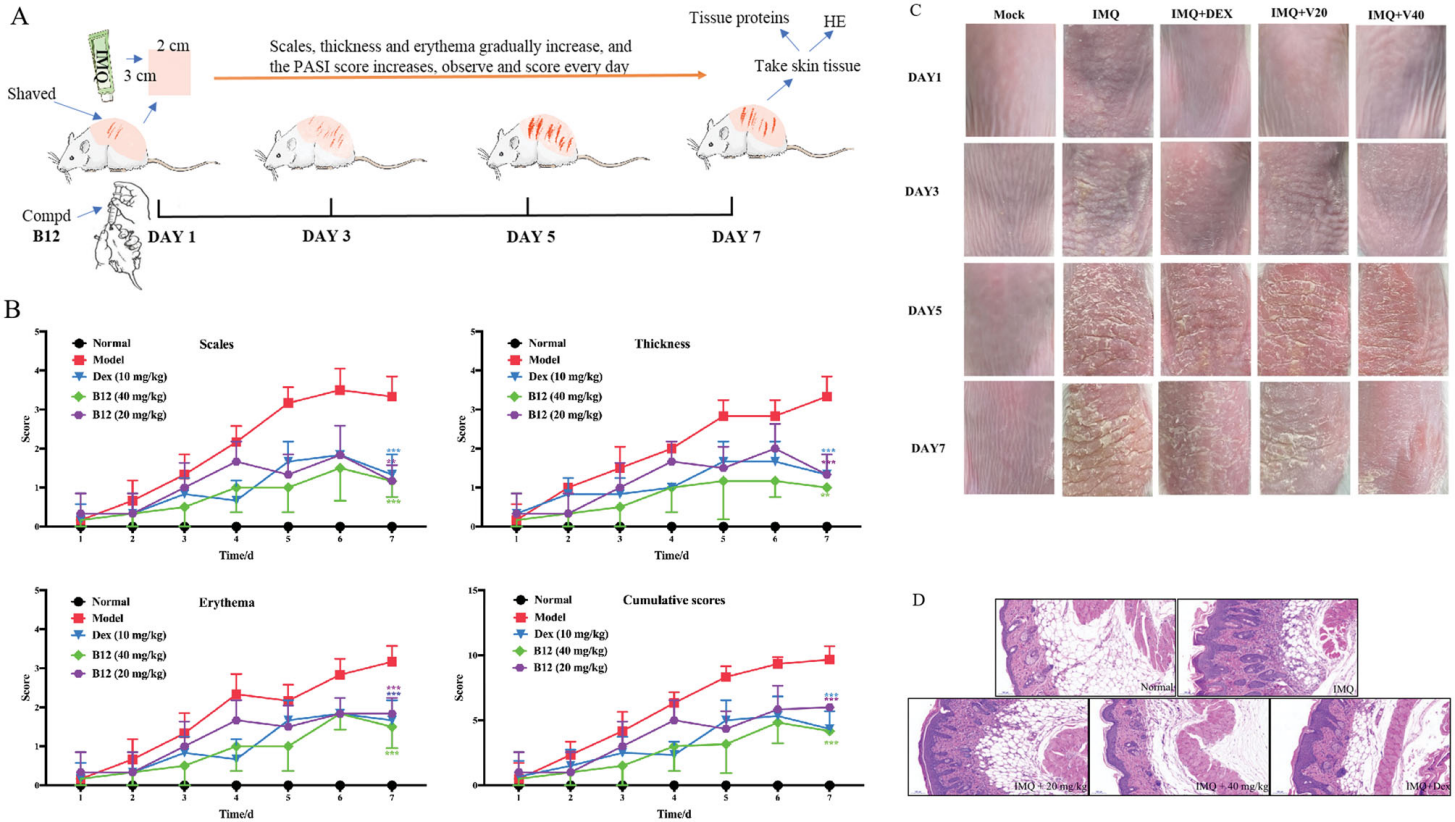
**(A)** Illustration of mouse psoriasis model induced by IMQ; **(B)** PASI and effect of compound **B12** on back skin of mice induced by IMQ (x ± s, *n* = 6); **(C)** Represent phenotypic pictures of back skins in each group; **(D)** H&E staining of skin tissues from mice in each group. Data were shown as mean ± SEM; *n* = 6 mice per group; *p < 0.05, **p < 0.01, and ***p < 0.001, compared with vehicle group.

The anti-inflammatory activity of compound **B12** was further evaluated *in vivo*. An IMQ-induced psoriasis mouse model was used to mimic the clinical manifestations of psoriasis patients^36^^,^^37^. As shown in Figure 8(A), severe skin lesions were occurred on the back skin, including scales, thickness and erythema during consecutive application of IMQ cream on BALB/c mice for 7 days in model the groups. Compound **B12** significantly attenuated experimental symptoms and PASI score in a dose-dependent manner during treatment, as well as in the dexamethasone-treated group (Figure 8(B,C)). HE analysis of skin tissue was performed to assess the levels of inflammation and tissue changes. Representative histological images of the tissue sections are shown in Figure 8(D). Mice induced by IMQ showed pathological psoriatic lesions, including loss of granular layer, epidermal hyperplasia, thickening of the acanthosis cell layer, parakeratosis, and inflammatory cell infiltration. IMQ-mice treated with compound **B12** (20 mg/kg) showed moderate pathological reduction, while IMQ-mice treated with compound **B12** (40 mg/kg) showed high pathological reduction as well as the positive drug dexamethasone.

**Figure 9.**
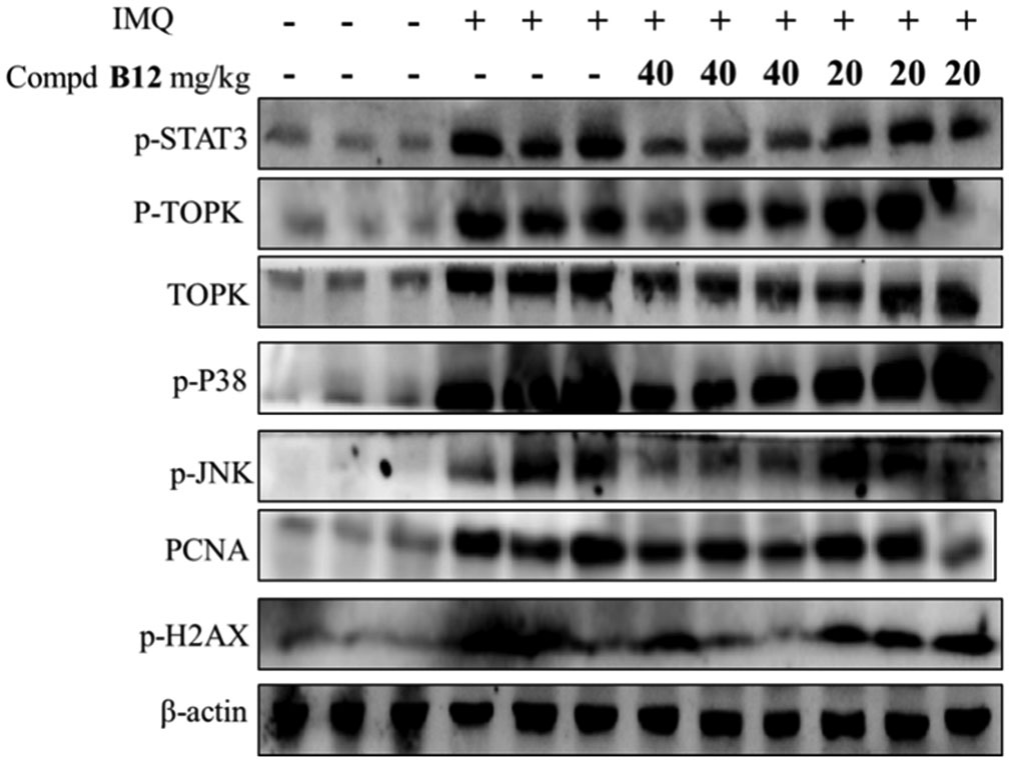
Compound **B12** inhibited expression of p-STAT3, p-TOPK, TOPK, p-p38, p-JNKs, PCNA, p-H2AX in mouse skin tissues. The results were showed as means ± SD (*n* = 3) of at least three independent experiments.

These results indicated that compound **B12** successfully reduced the scales, thickness and erythema in psoriasis-like mice, histopathologically alleviated hyperkeratosis, acanthocyte proliferation and inflammatory cell infiltration. As shown in Figure 9, the expression of related proteins in mouse skin tissues was decreased significantly after treatment with compound **B12** in a dose-dependent manner. It is worth noting that compound **B12** inhibits the expression of proliferation-related protein STAT3 and precancerous factor PCNA in skin tissues.

Collectively, these results demonstrate that compound **B12** could improve psoriasis-like skin inflammation in *vivo*.

### 3.10. Metabolic stability assay in liver microsomes

With the above information, the metabolic stability of compound **B12** was further evaluated in human liver microsomes *in vitro*. As shown in Table 2, compound **B12** showed acceptable stability against the metabolism of human liver microsomes with a half-life (t_1/2_) of 111.1 min. This result indicated that compound **B12** might be selected as a promising candidate for the treatment of psoriasis-like skin inflammation.

**Table 2.** Metabolic stability in human liver microsomes.

Compound	Human		
	T_1/2_ (min)	*In vitro* CL_int_ (µL/min/mg protein)	CL_hep_ (mL/min/kg)
**B12**	111.1	12.5	8.5
Dextromethorphan	39.5	35.1	13.7

## 4. Conclusions

**Figure 10.**
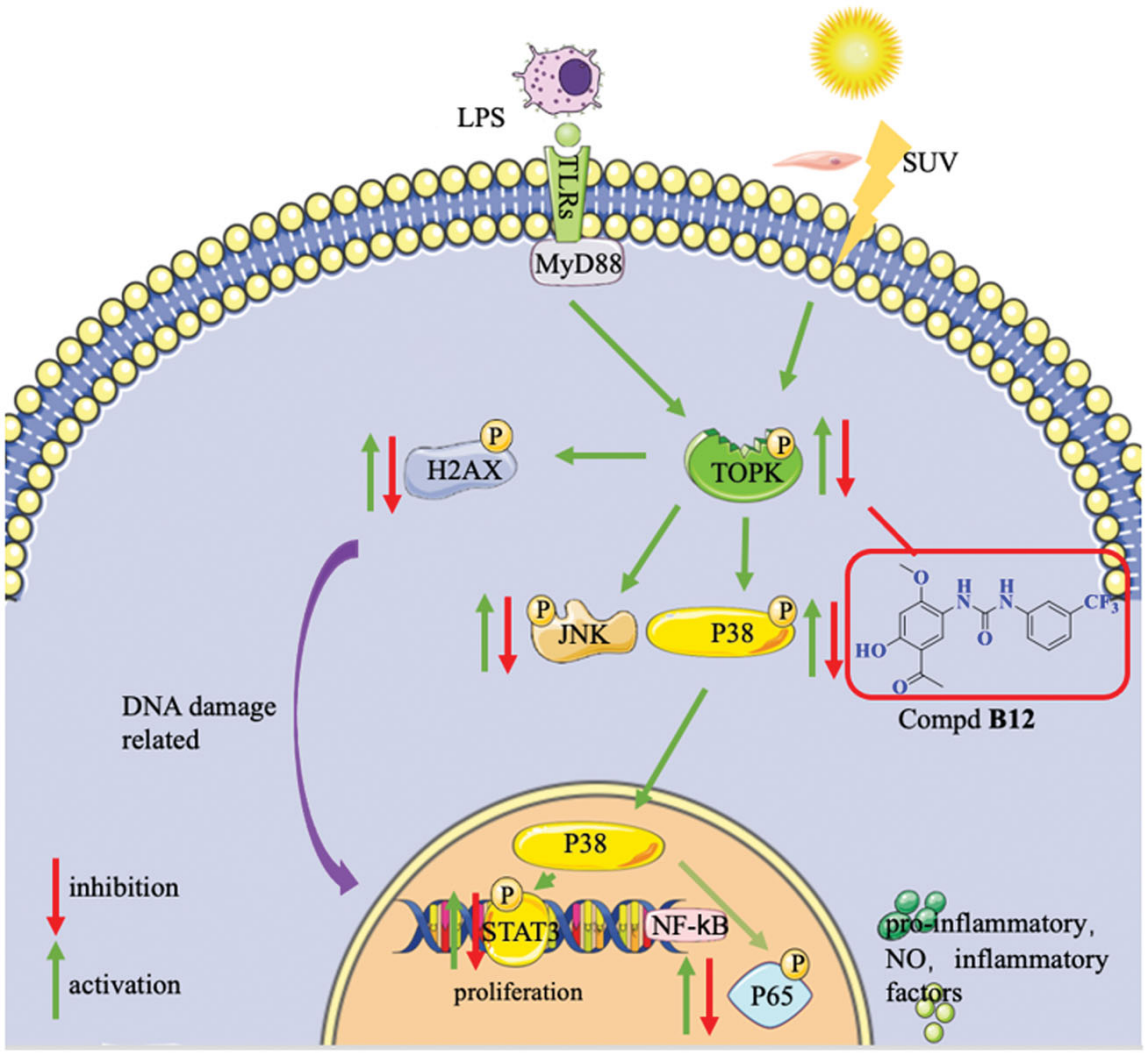
Proposed mechanisms of compound **B12** for anti-inflammatory.

In summary, 38 new paeonol derivatives were synthesised and their anti-inflammatory activities were evaluated. Title compound **B12** was found to be the most potent compound without obvious cytotoxicity. The preliminary mechanism indicated that compound **B12** could interact with TOPK, to regulating its downstream pathways MAPK and NF-κB and inhibit the expression of DNA damage-related protein H2AX and proliferation-related protein STAT3 (Figure 10). Anti-inflammatory studies revealed that this compound could effectively relieve histological changes in IMQ-induced murine psoriasis-like skin inflammation.

## Supplementary Material

Supplemental Material
