# Supplementary material for: Discovery of novel paeonol-based derivatives against skin inflammation in vitro and in vivo
Source: J Enzyme Inhib Med Chem. 2022 Feb 28;37(1):817–31. doi: 10.1080/14756366.2022.2043852 (PMC8890542; doi:10.1080/14756366.2022.2043852)
Supplement: Supplemental Material [file IENZ_A_2043852_SM3341.pdf]

Supporting Information

1. ADMET Prediction……………………………………………………S1-S3

2. ^{1}H NMR, ^{13}C NMR and HRMS spectra of all title compounds………. S4-S58

3. Purity data analyzed by HPLC of Compounds…………..………….. S58-S63

2. ADMET Prediction

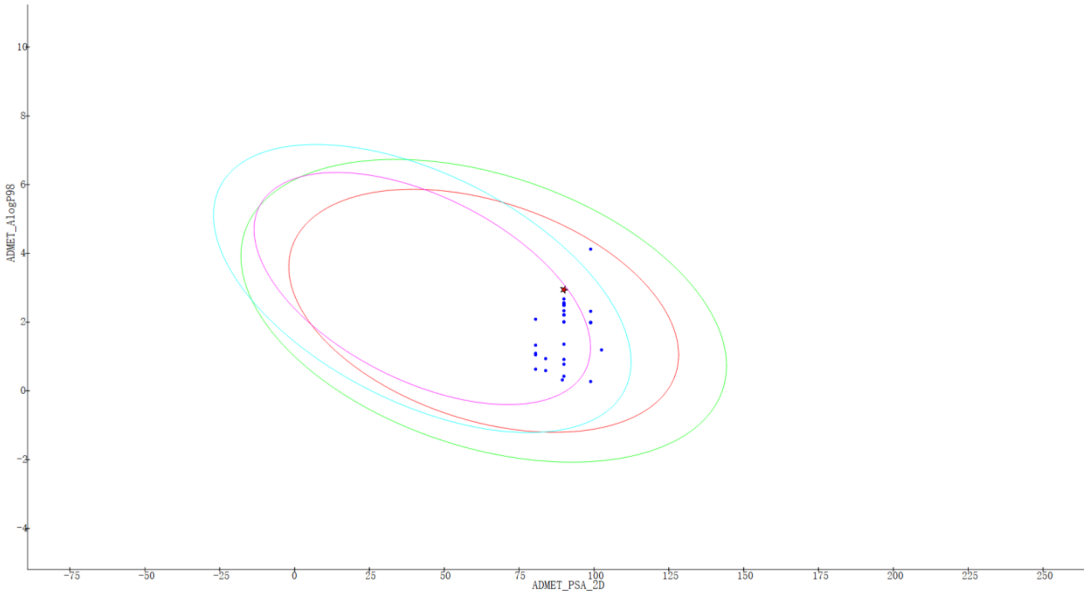

Figure 4. Regression of ADMET_PSA_2D and ADMET_AlogP98. Red represents the 95% confidence interval of HIA, and green denotes the 99% confidence interval of HIA. Pink indicates the 95% confidence interval of BBB, and the sky-blue ellipse signifies the 99% confidence interval of BBB. All compounds are within the confidence interval.

Table 1. ADMET prediction for compounds **A1~A14**, **B1~B13**, **C1~C7** and **D1~D4**

Compd	Solubility level^a	BBB level^b	CYP2D6^c	Absorption level^d	PPB^e	AlogP98^f	PSA 2D^g
**A1**	3	3	FALSE	0	FALSE	0.776	89.967
**A2**	3	3	FALSE	0	FALSE	0.427	89.967
**A3**	3	3	FALSE	0	FALSE	1.331	80.509
**A4**	3	3	FALSE	0	TRUE	2.086	80.509
**A5**	3	3	FALSE	0	FALSE	0.273	98.897
**A6**	2	3	FALSE	0	TRUE	2.564	89.967
**A7**	3	3	FALSE	0	FALSE	0.633	80.509
**A8**	3	3	FALSE	0	TRUE	1.359	89.967
**A9**	3	3	FALSE	0	TRUE	0.917	89.967
**A10**	3	3	FALSE	0	FALSE	0.591	83.862
**A11**	3	3	FALSE	0	FALSE	0.321	89.44
**A12**	3	3	FALSE	0	FALSE	1.094	80.509
**A13**	3	3	FALSE	0	FALSE	0.939	83.862

**A14**	3	3	FALSE	0	FALSE	1.048	80.509
**B1**	3	3	FALSE	0	TRUE	1.193	102.521
**B2**	3	3	FALSE	0	TRUE	2.004	89.967
**B3**	3	3	FALSE	0	TRUE	2.49	89.967
**B4**	3	3	FALSE	0	TRUE	2.49	89.967
**B5**	3	3	FALSE	0	TRUE	2.49	89.967
**B6**	3	3	FALSE	0	TRUE	2.209	89.967
**B7**	3	3	FALSE	0	TRUE	2.209	89.967
**B8**	3	3	FALSE	0	TRUE	2.209	89.967
**B9**	3	3	FALSE	0	TRUE	1.987	98.897
**B10**	3	3	FALSE	0	TRUE	1.987	98.897
**B11**	3	3	FALSE	0	TRUE	1.988	98.897
**B12**	2	3	FALSE	0	TRUE	2.946	89.967
**B13**	2	3	FALSE	0	TRUE	2.946	89.967
**C1**	2	3	FALSE	0	TRUE	4.124	98.897
**C2**	3	3	FALSE	0	TRUE	2.675	89.967
**C3**	3	3	FALSE	0	TRUE	2.011	89.967
**C4**	3	3	FALSE	0	TRUE	2.497	89.967
**C5**	3	3	FALSE	0	TRUE	2.216	89.967
**C6**	3	3	FALSE	0	TRUE	1.994	98.897
**C7**	3	3	FALSE	0	TRUE	1.994	98.897
**D1**	3	3	FALSE	0	TRUE	2.332	89.967
**D2**	3	3	FALSE	0	TRUE	2.538	89.967
**D3**	3	3	FALSE	0	TRUE	2.316	98.897
**D4**	3	3	FALSE	0	TRUE	2.538	89.967

[a] Distribution: Aqueous solubility

[b] Distribution: Blood–brain barrier penetration

[c] Metabolism

[d] Absorption

[e] Excretion

[f] Predicted octanol/water

[g] Two-dimensional

[h] level 0 means good

[i] level 1 means very good

[j] level 2 means moderate

[k] level 3 means poor

2. ^{1}H NMR, ^{13}C NMR and HRMS spectra of all title compounds

^{1}H, ^{13}C NMR of compound **A1**

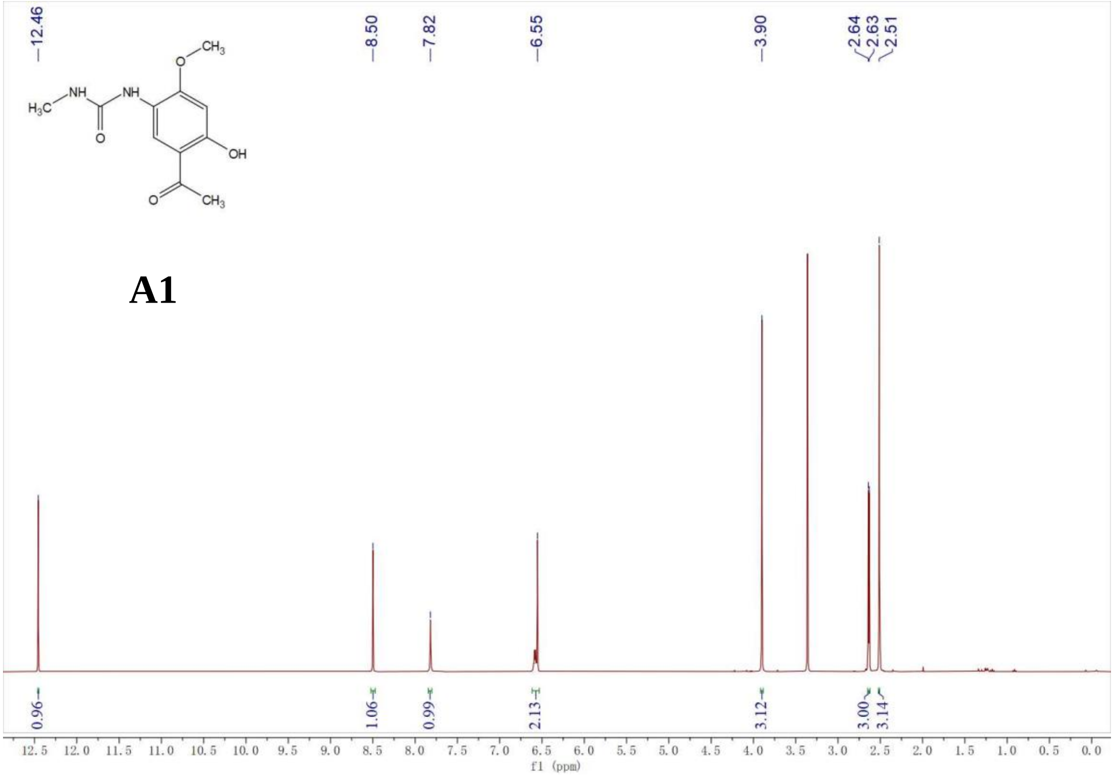

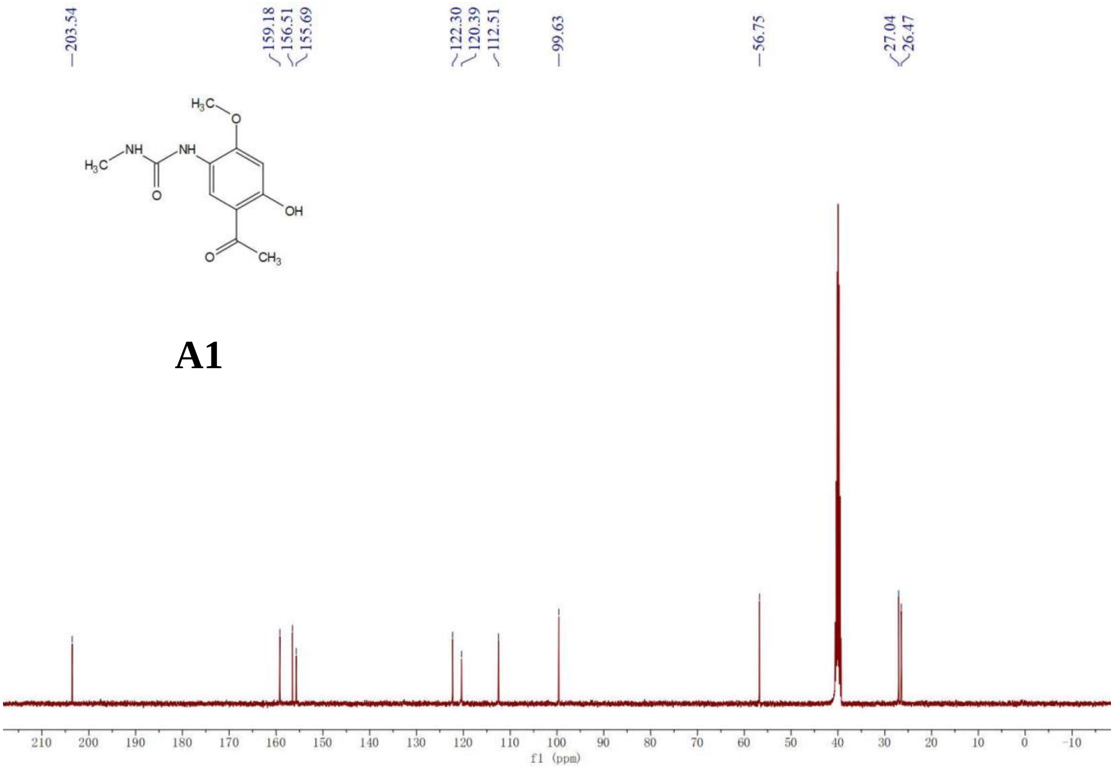

HRMS of compound **A1**

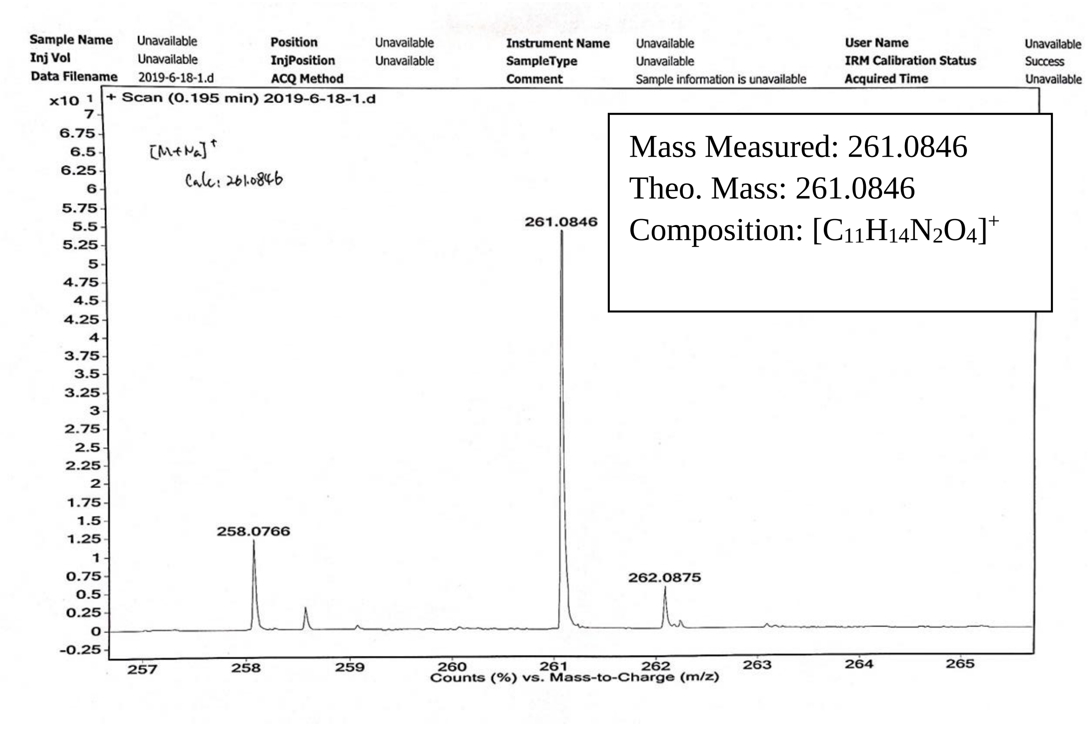

^{1}H, ^{13}C NMR of compound **A2**

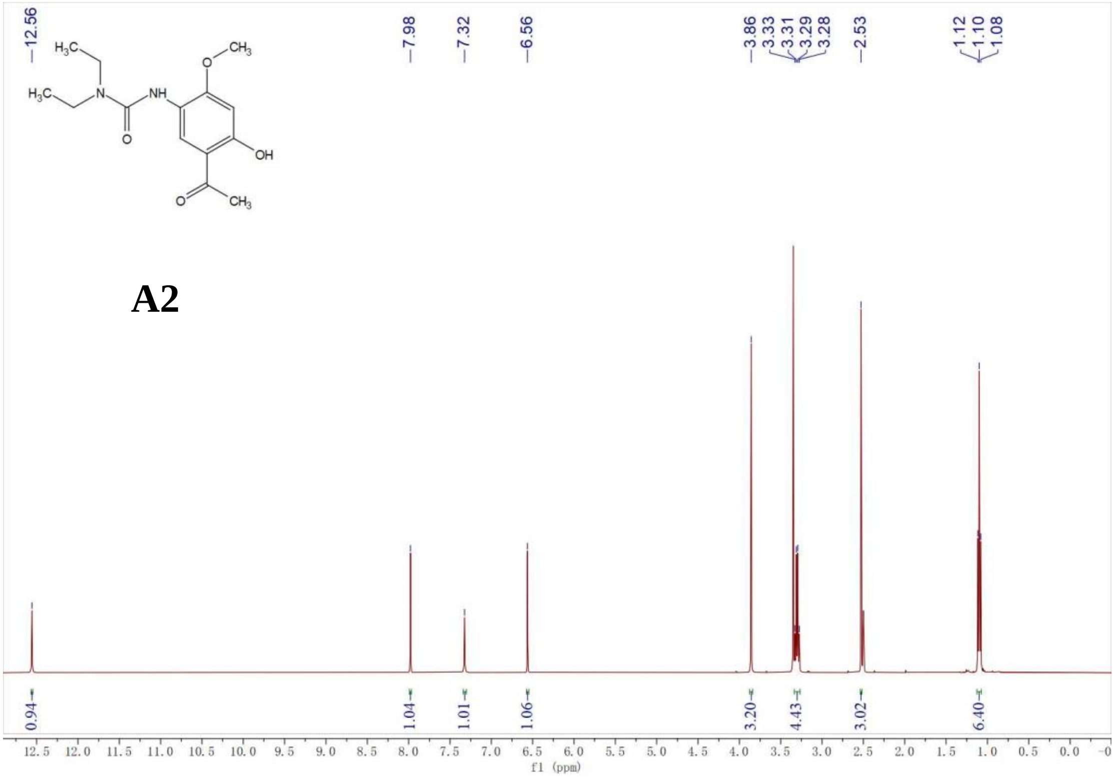

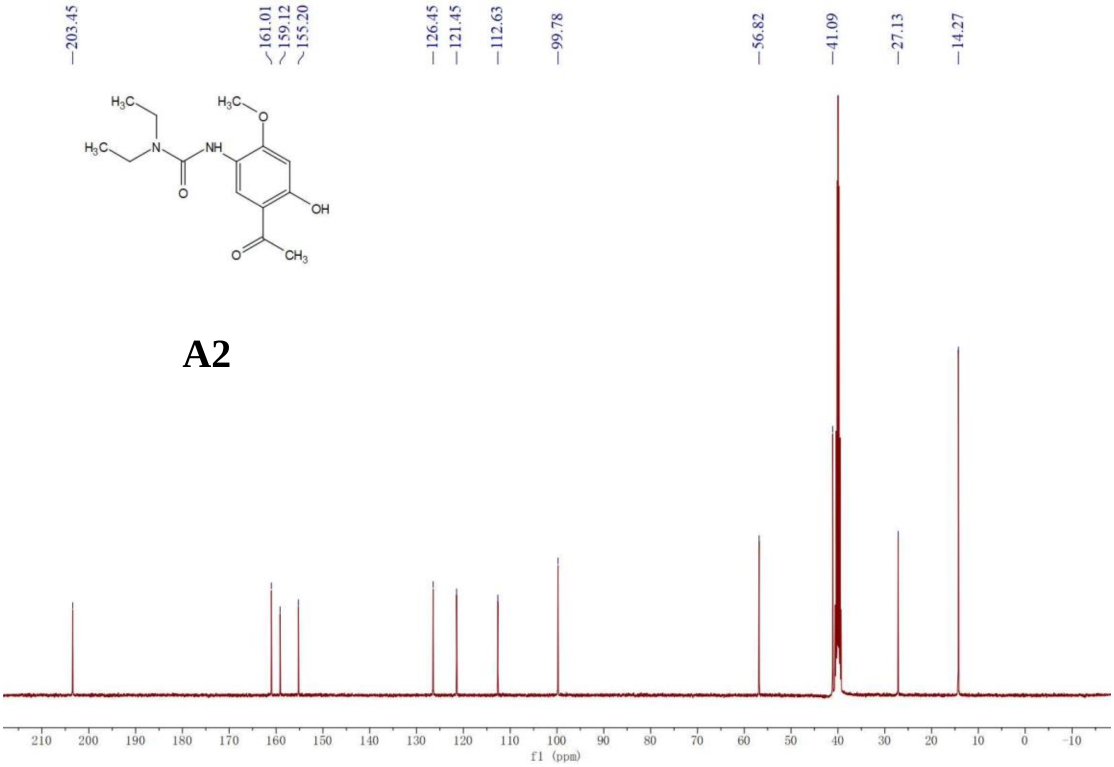

A2

HRMS of compound **A2**

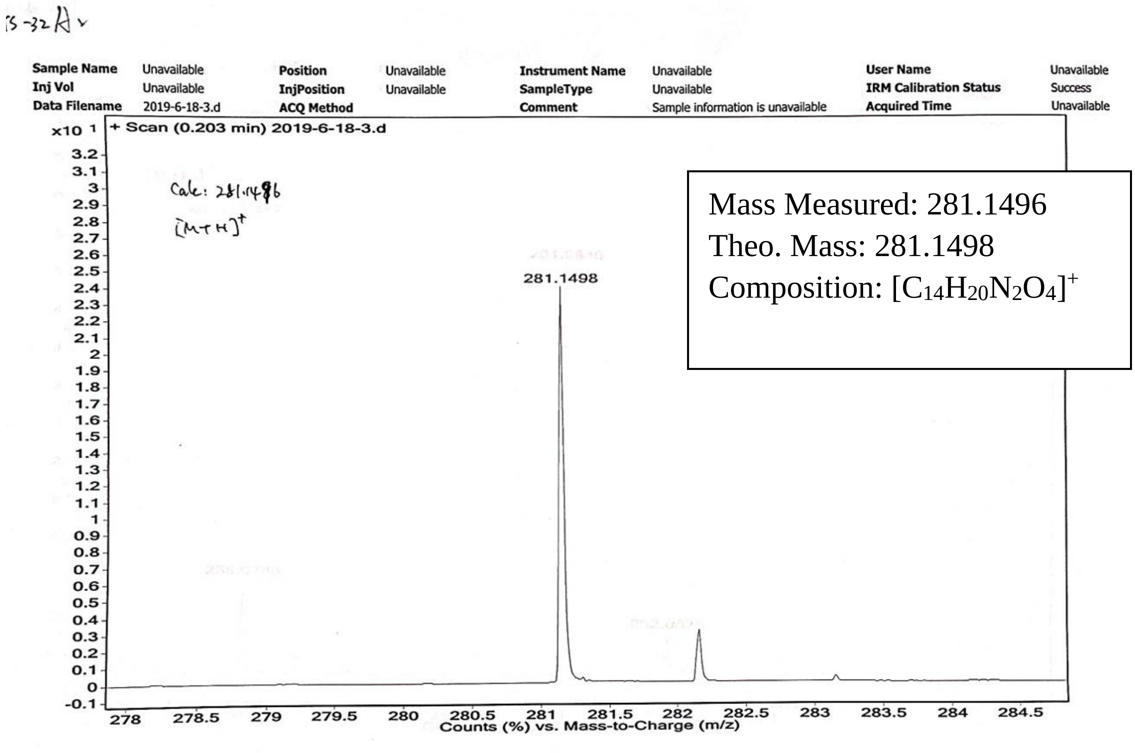

Mass Measured: 281.1496

Theo. Mass: 281.1498

Composition: $[C_{14}H_{20}N_2O_4]^+$

^{1}H,^{13}C NMR of compound **A3**

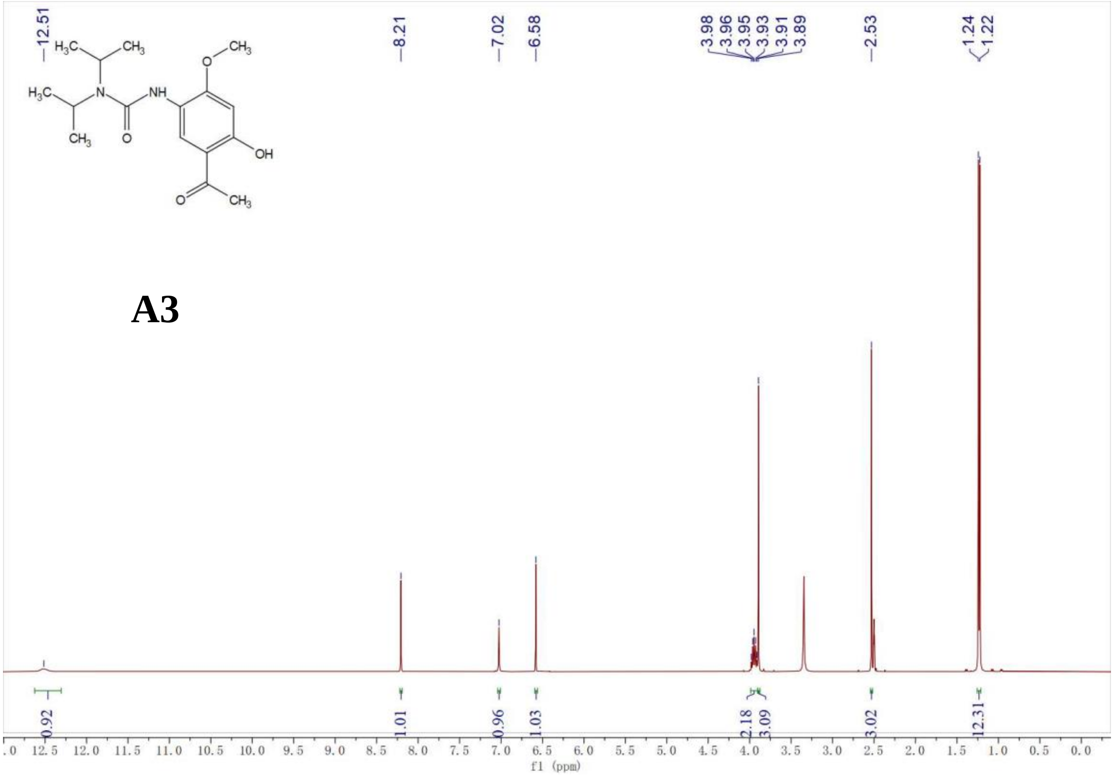

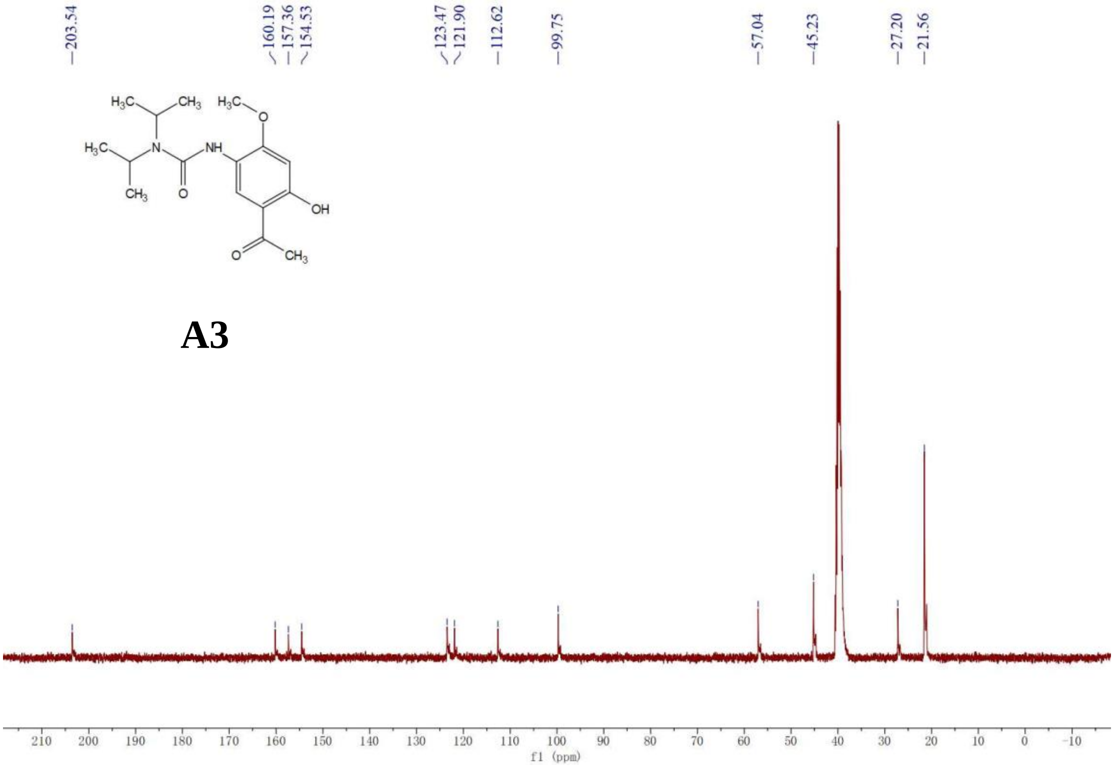

HRMSof compound **A3**

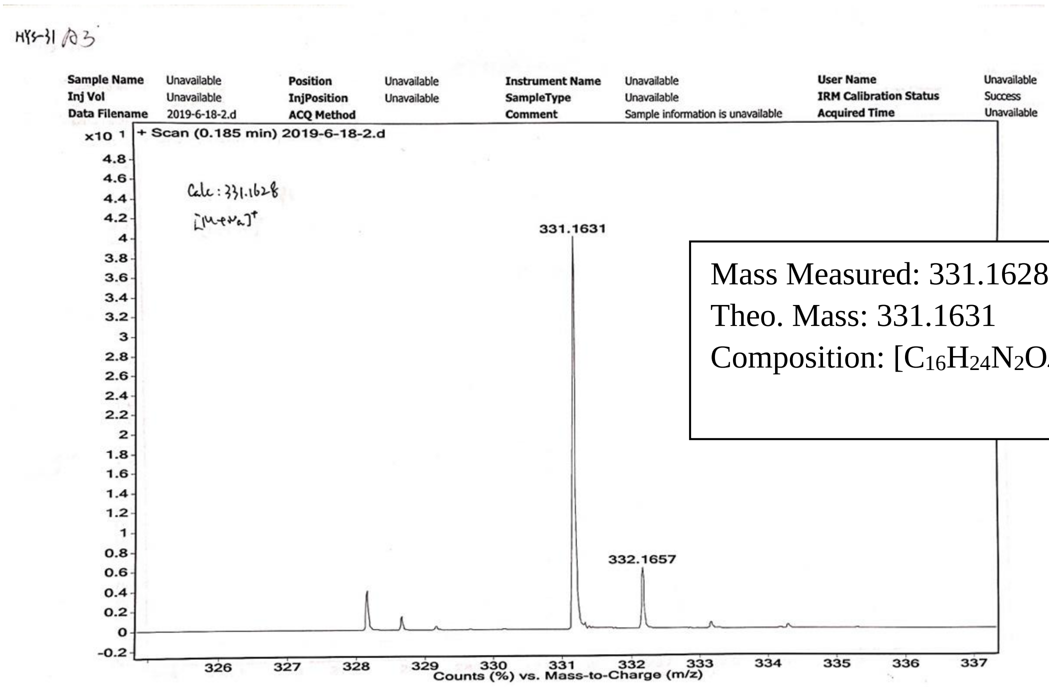

Mass Measured: 331.1628
Theo. Mass: 331.1631
Composition: $[C_{16}H_{24}N_2O_4]^+$

^{1}H,^{13}C NMR of compound **A4**

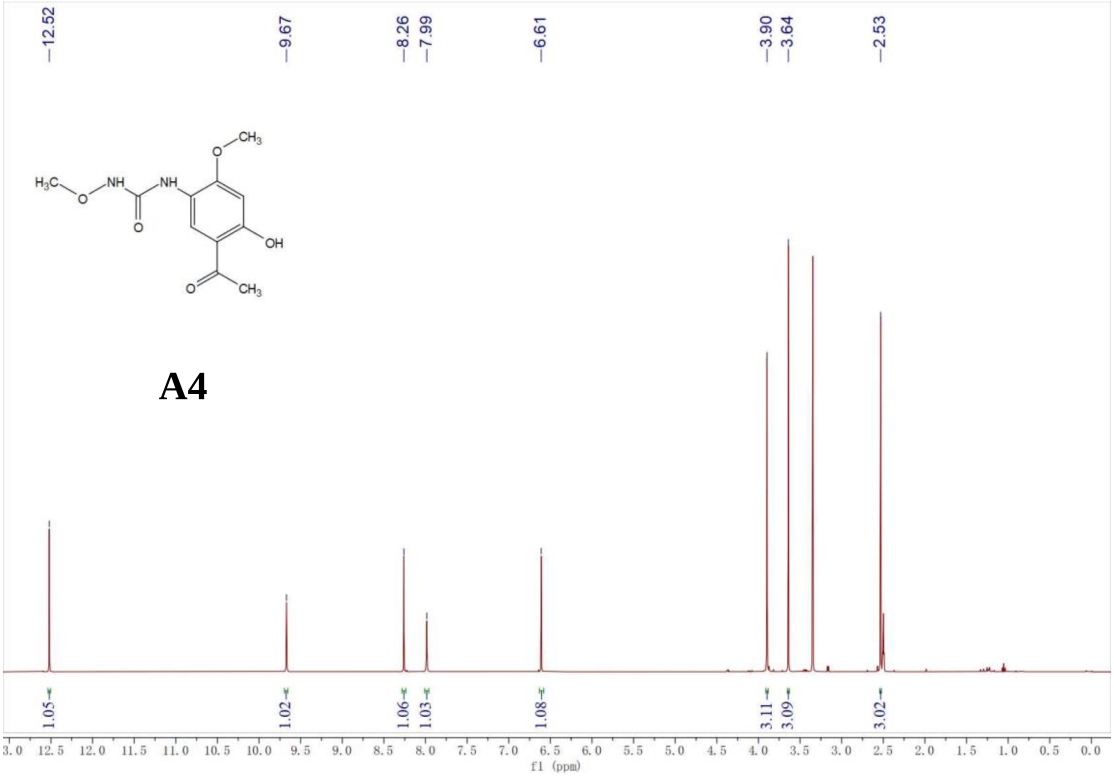

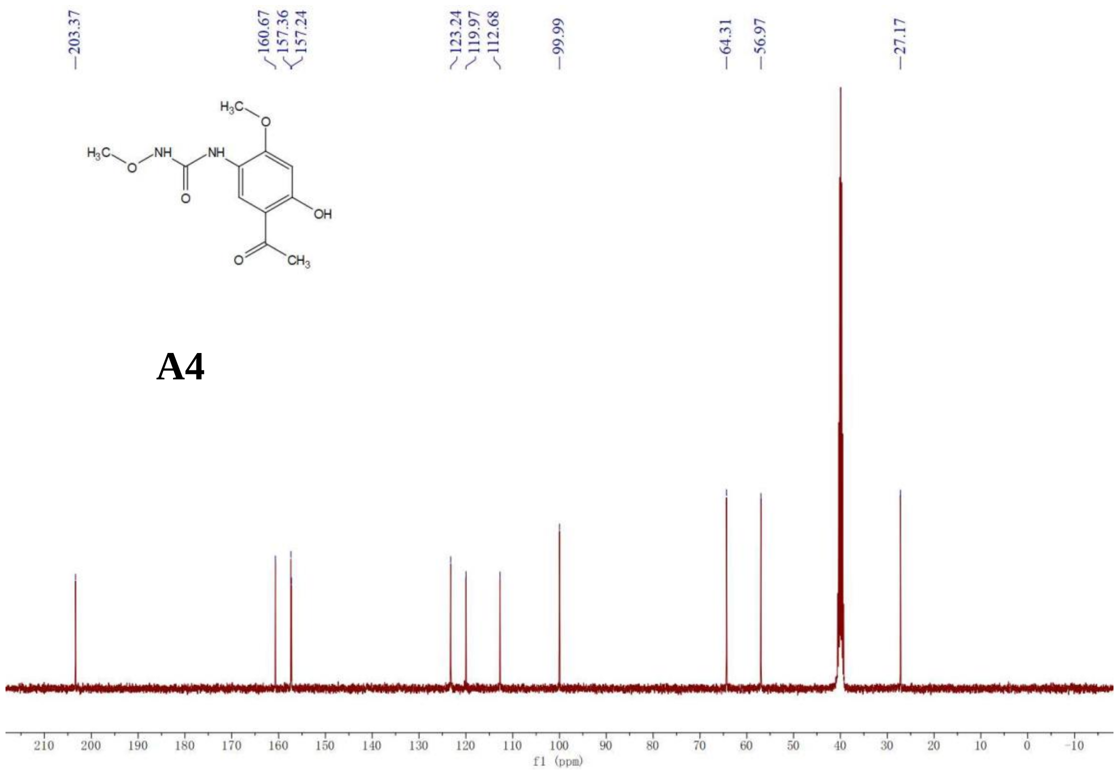

HRMS of compound **A4**

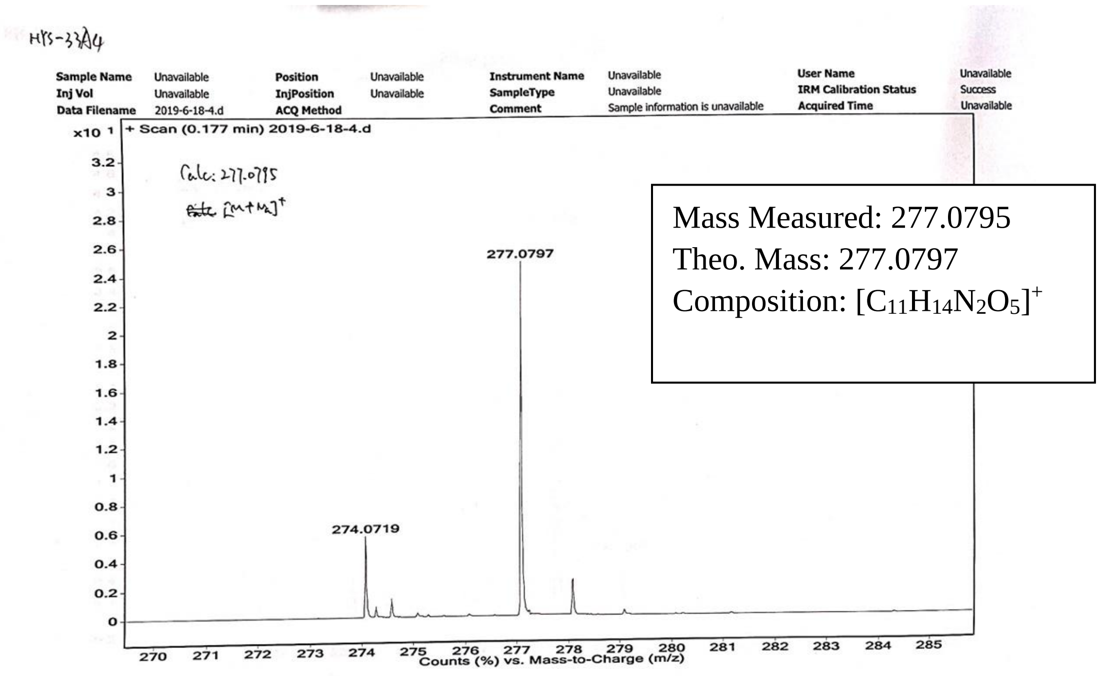

Mass Measured: 277.0795
Theo. Mass: 277.0797
Composition: $[C_{11}H_{14}N_2O_5]^+$

^{1}H,^{13}C NMRof compound **A5**

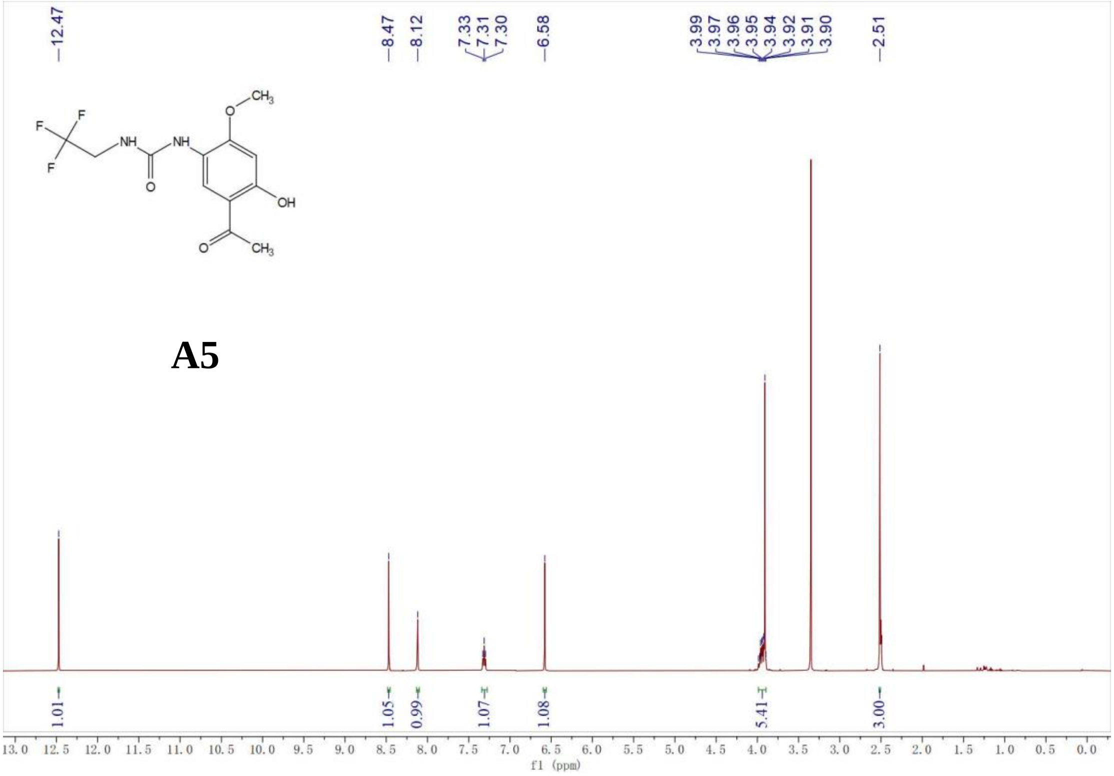

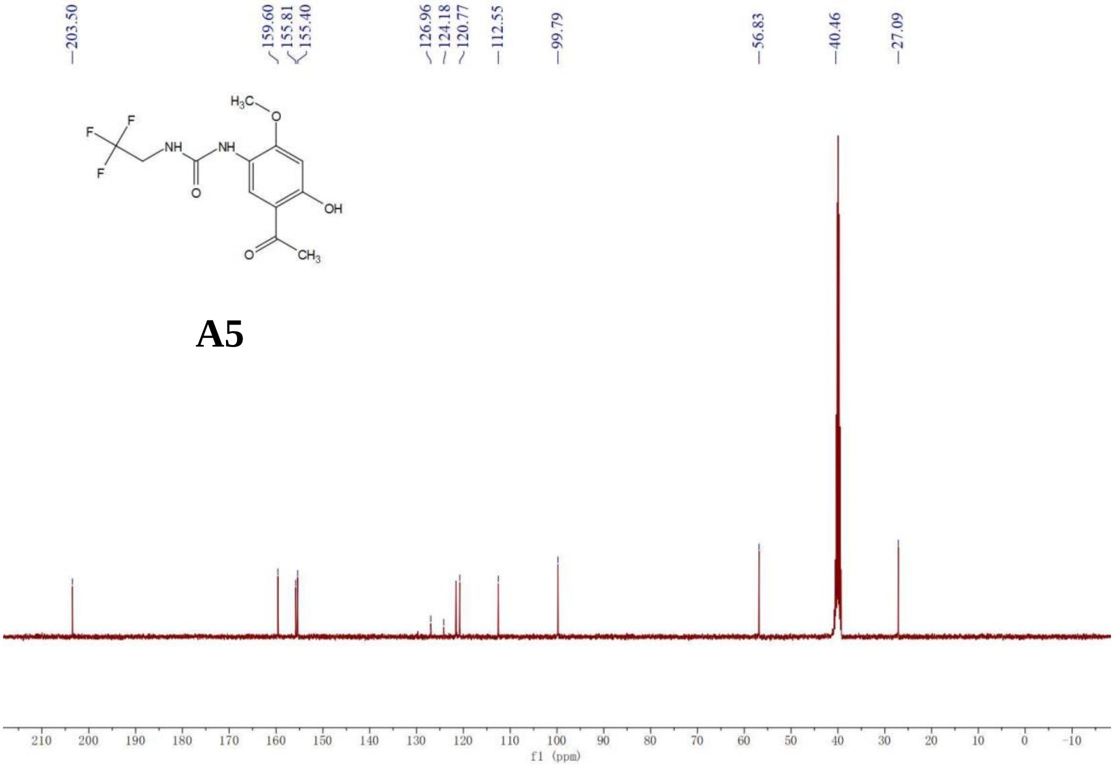

HRMS of compound **A5**

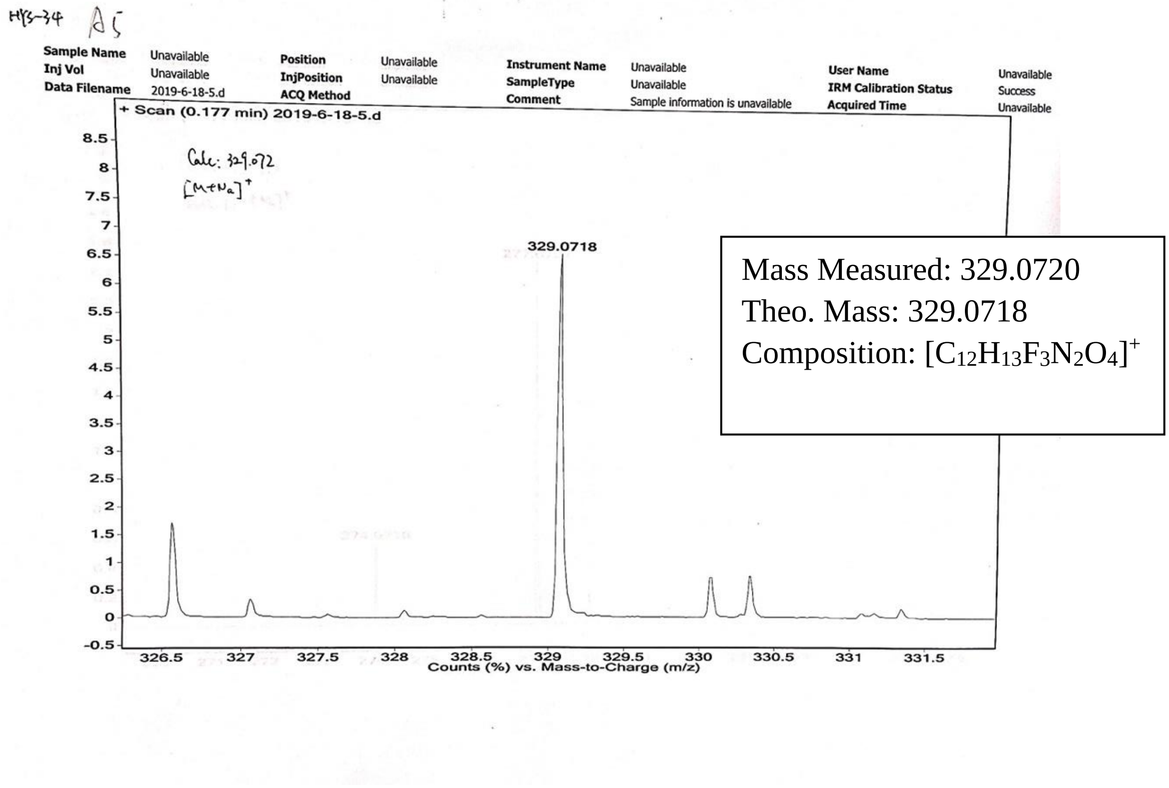

^{1}H,^{13}C NMR of compound **A6**

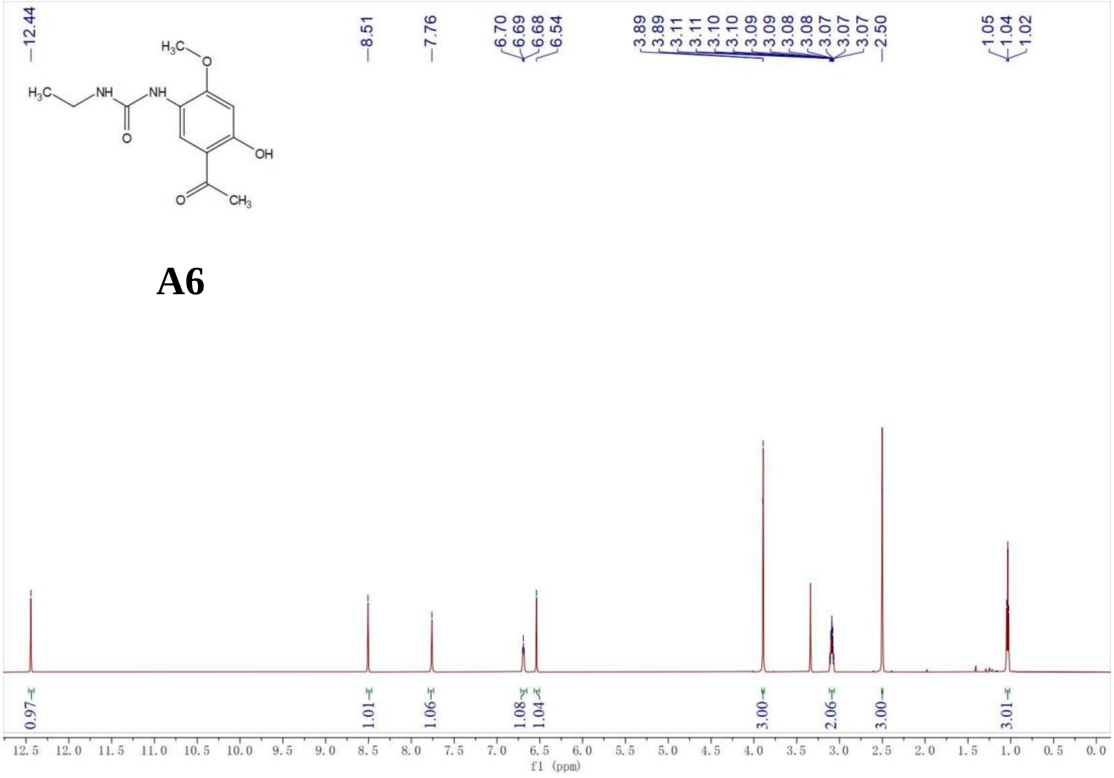

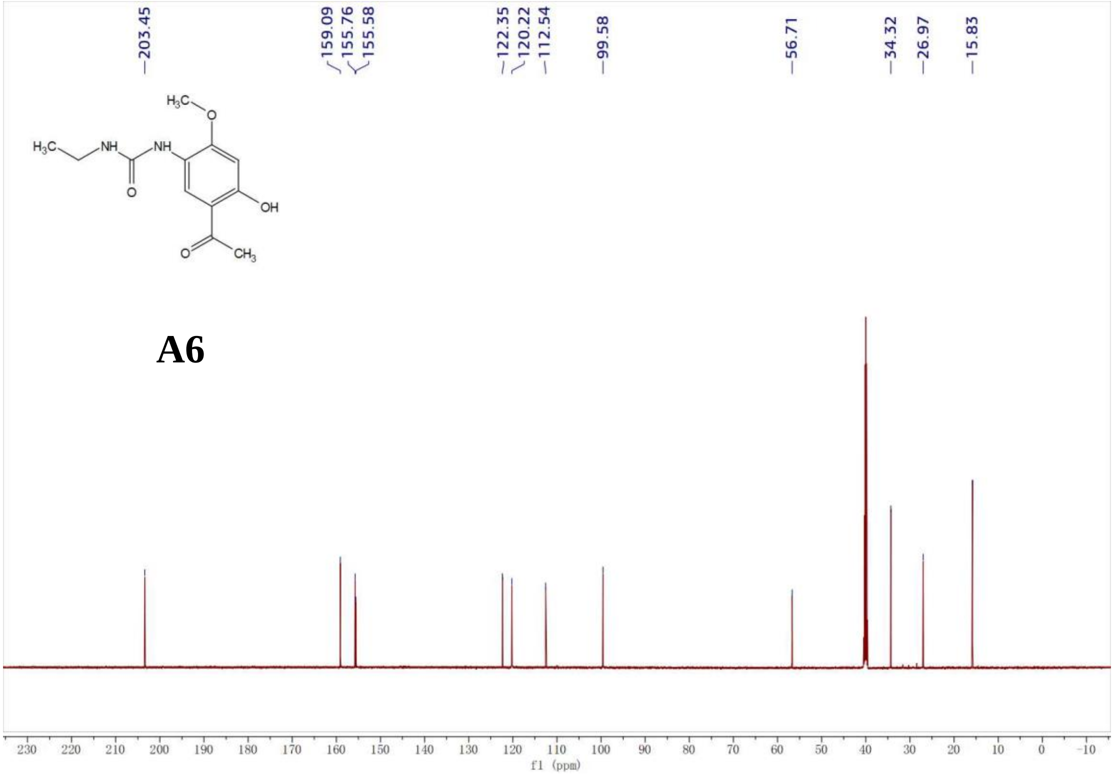

HRMS of compound **A6**

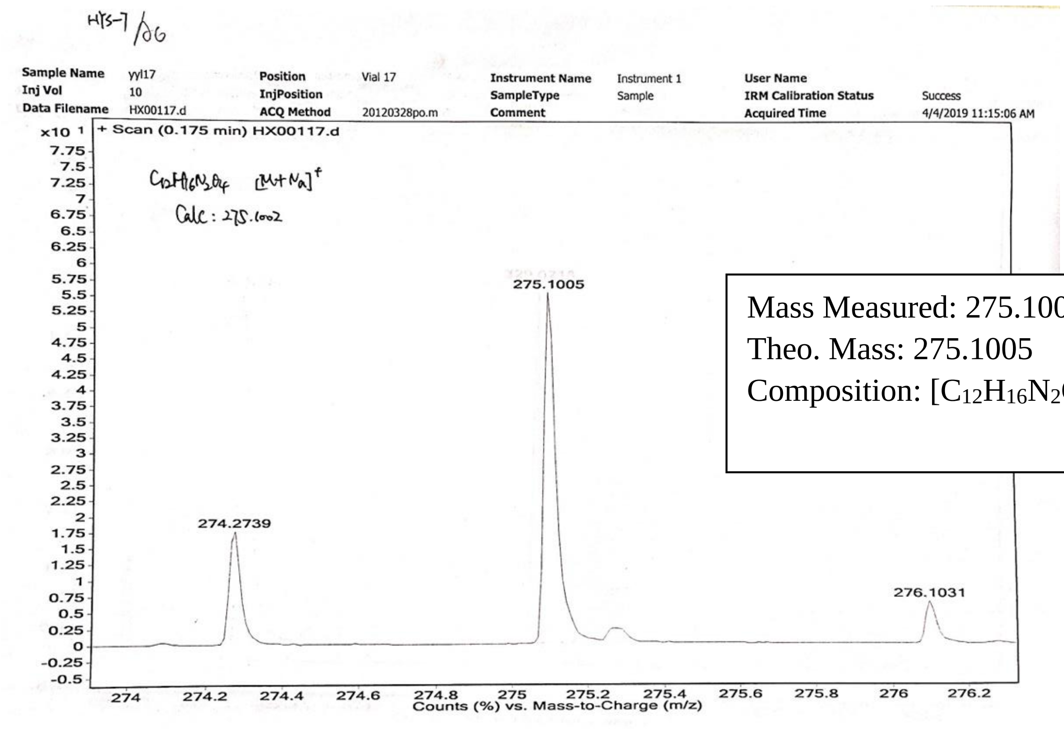

Mass Measured: 275.1002
Theo. Mass: 275.1005
Composition: $[C_{12}H_{16}N_2O_4]^+$

^{1}H,^{13}C NMR of compound **A7**

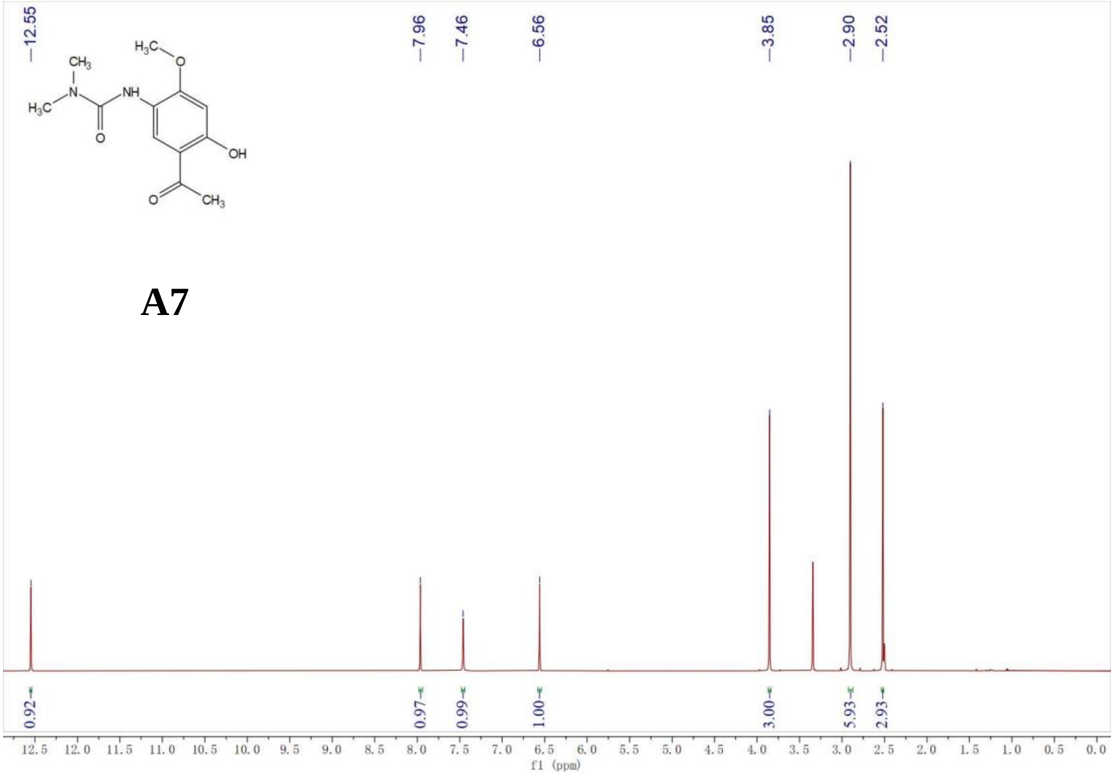

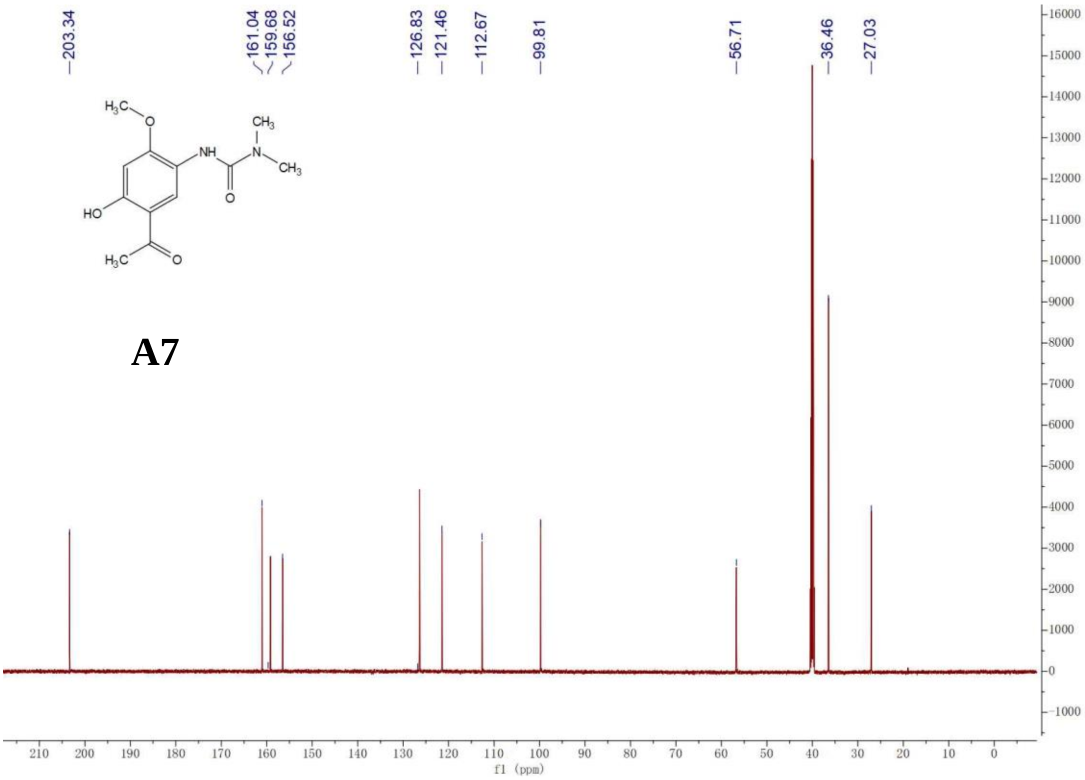

HRMS of compound **A7**

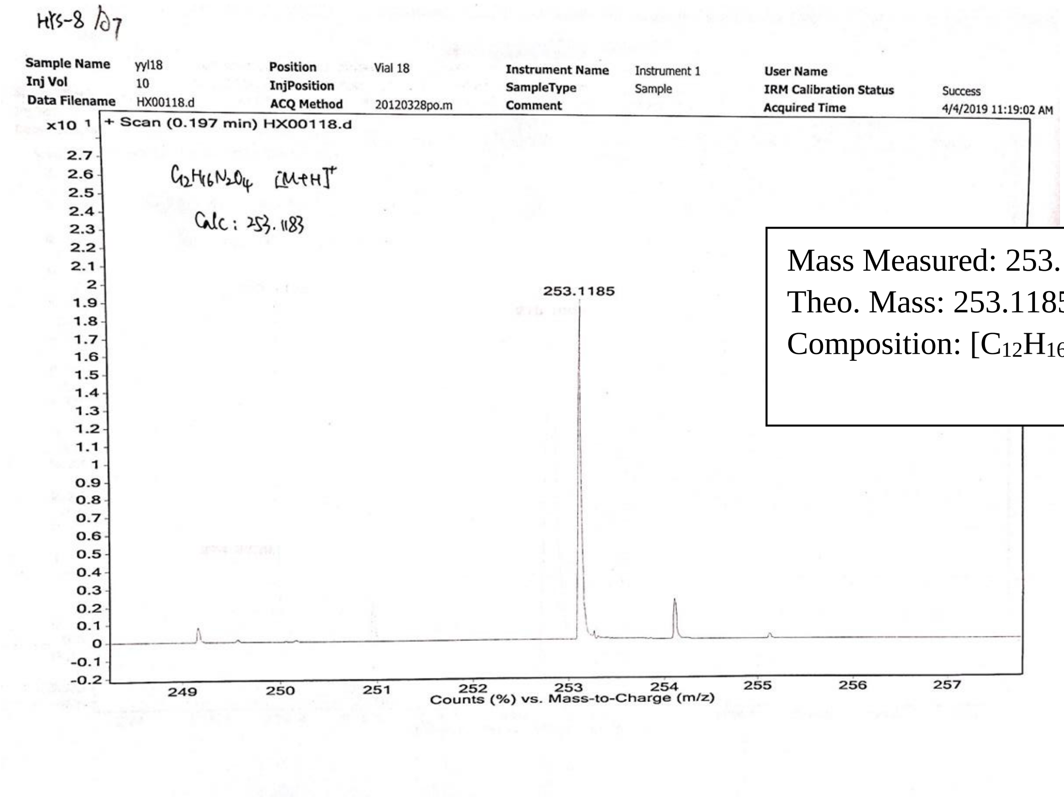

Mass Measured: 253.1183
Theo. Mass: 253.1185
Composition: $[C_{12}H_{16}N_2O_4]^+$

^{1}H,^{13}C NMR of compound **A8**

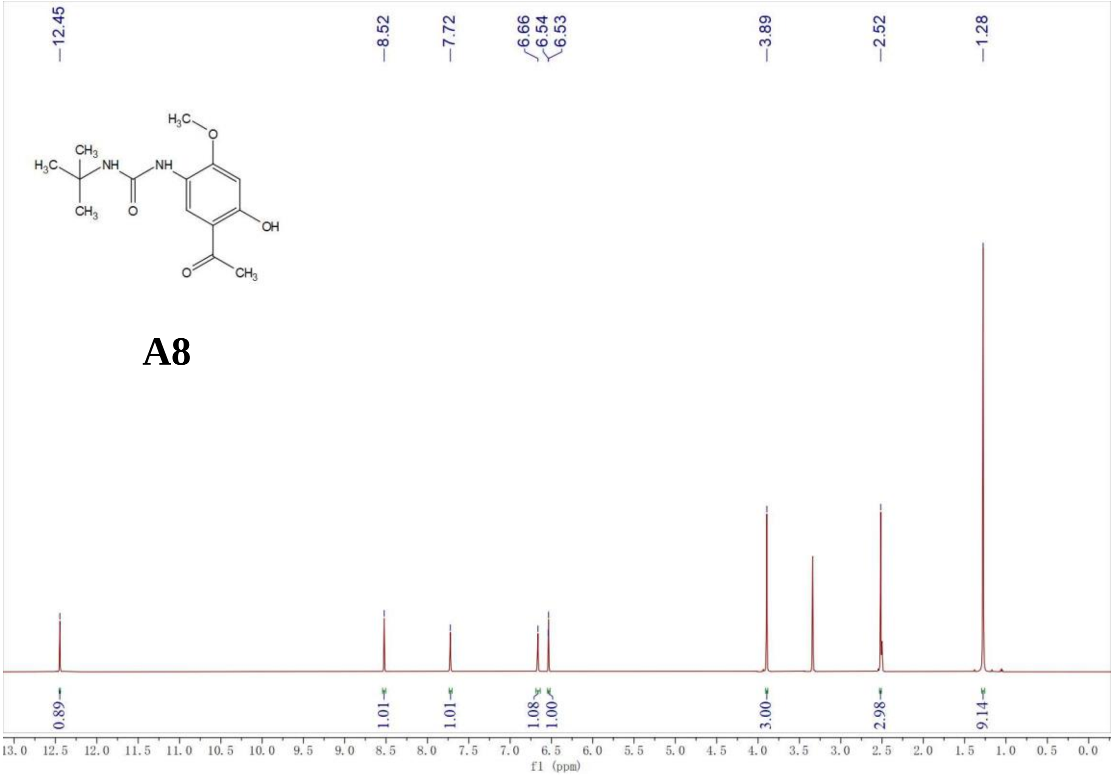

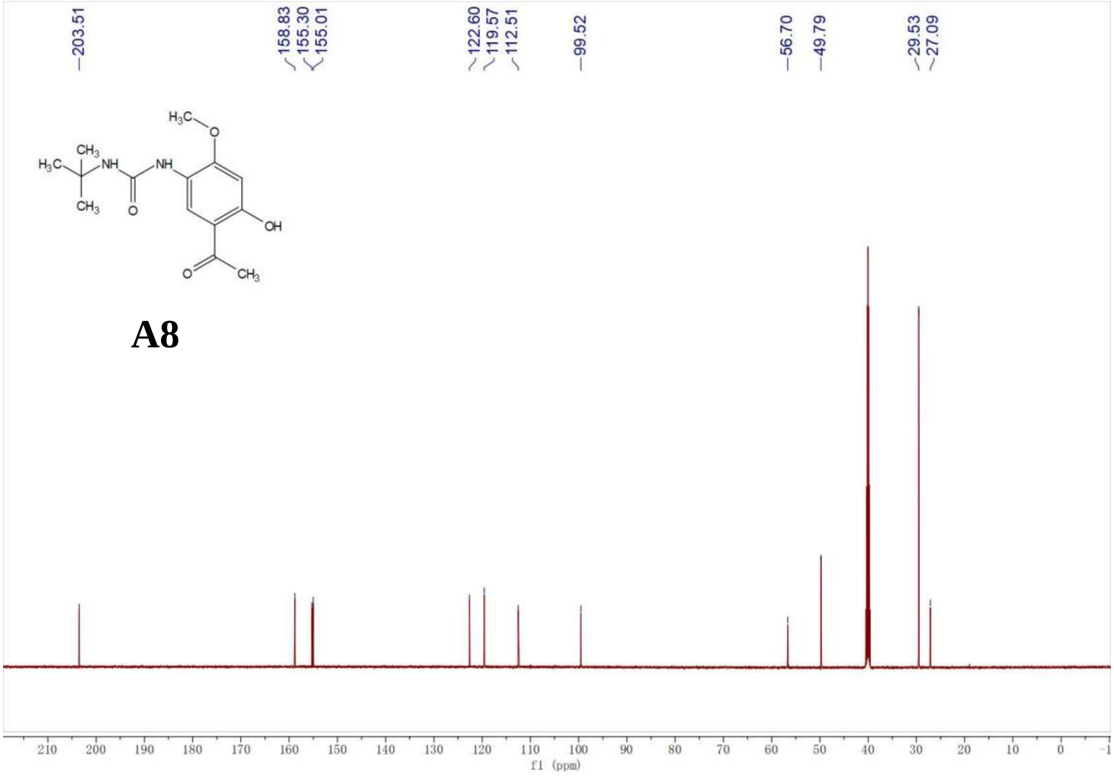

HRMS of compound **A8**

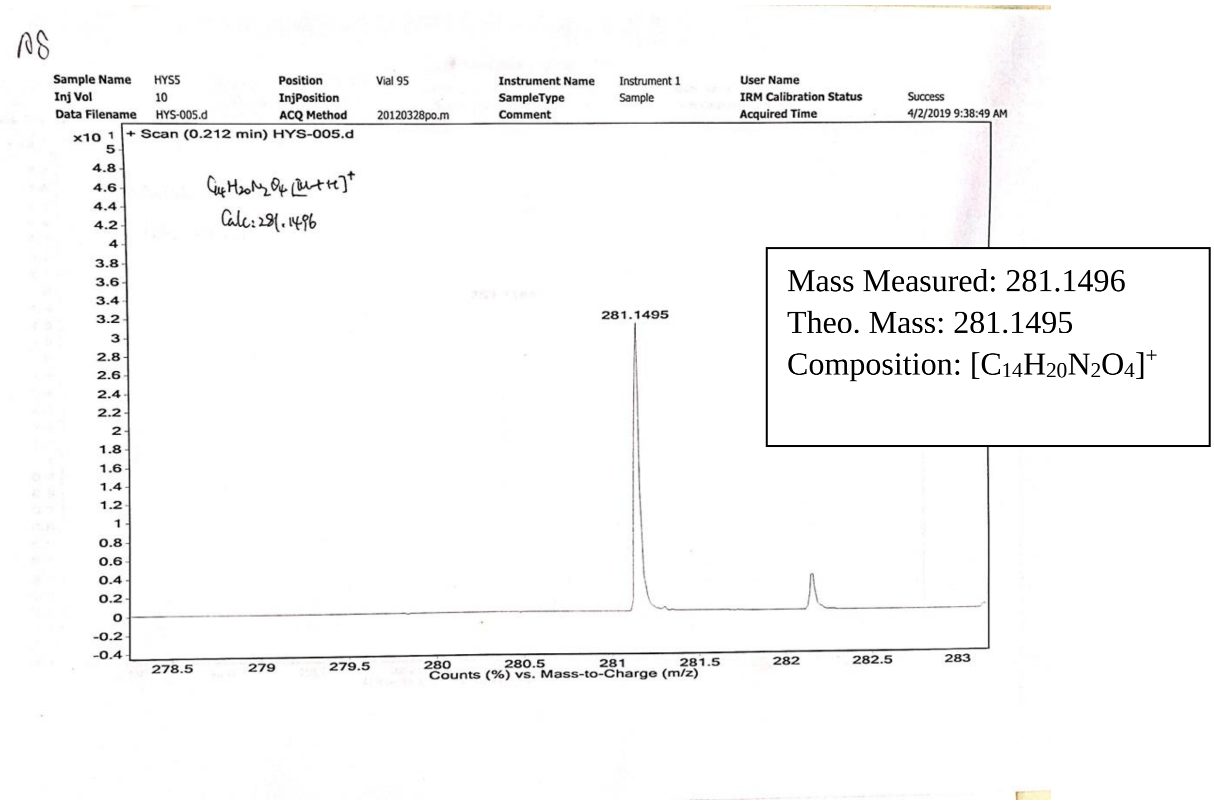

Mass Measured: 281.1496
Theo. Mass: 281.1495
Composition: $[C_{14}H_{20}N_2O_4]^+$

^{1}H,^{13}C NMR of compound **A9**

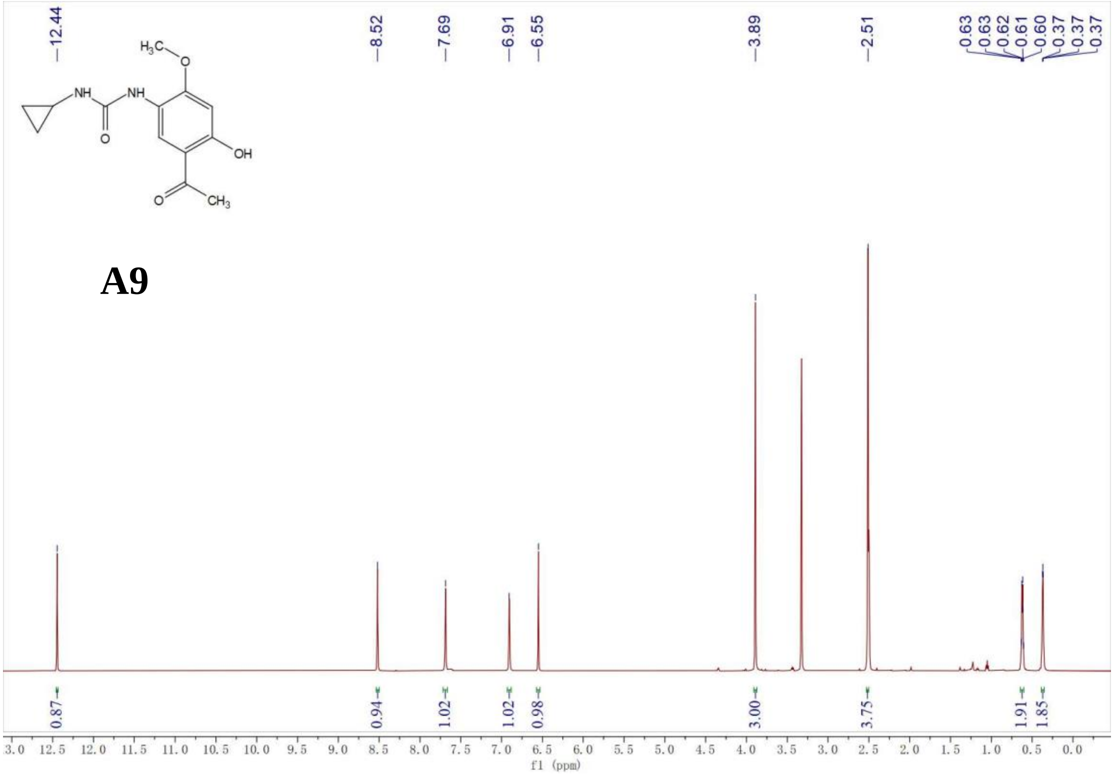

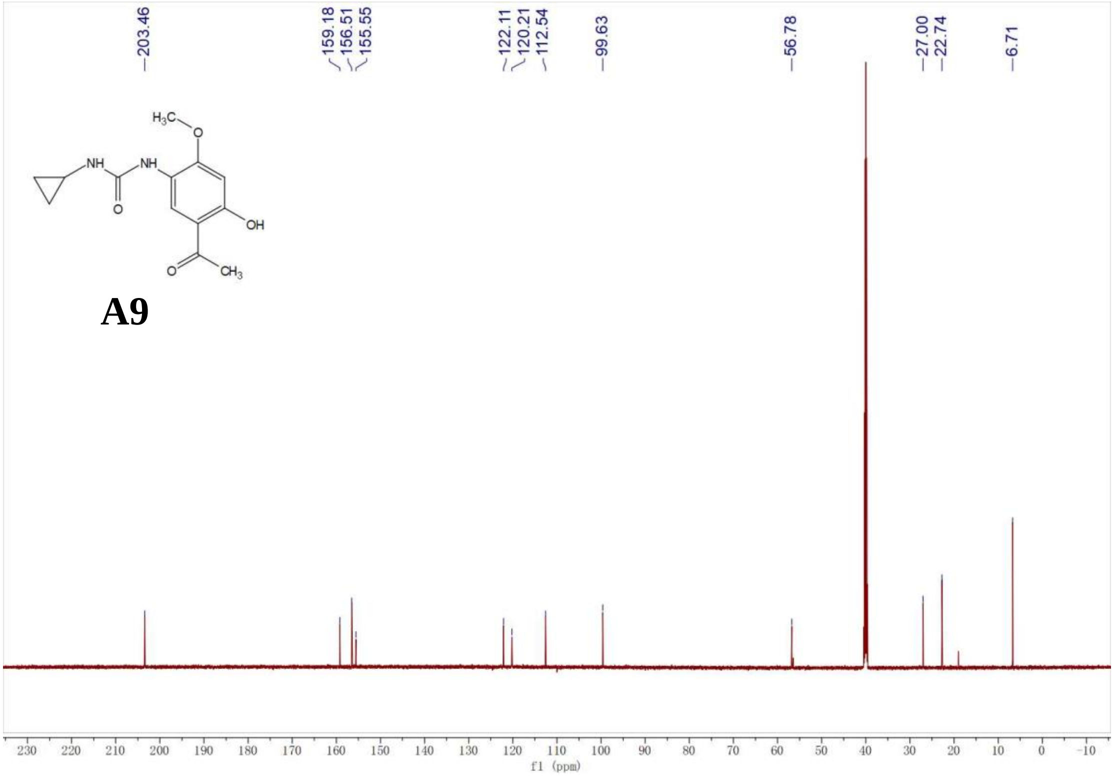

HRMS of compound **A9**

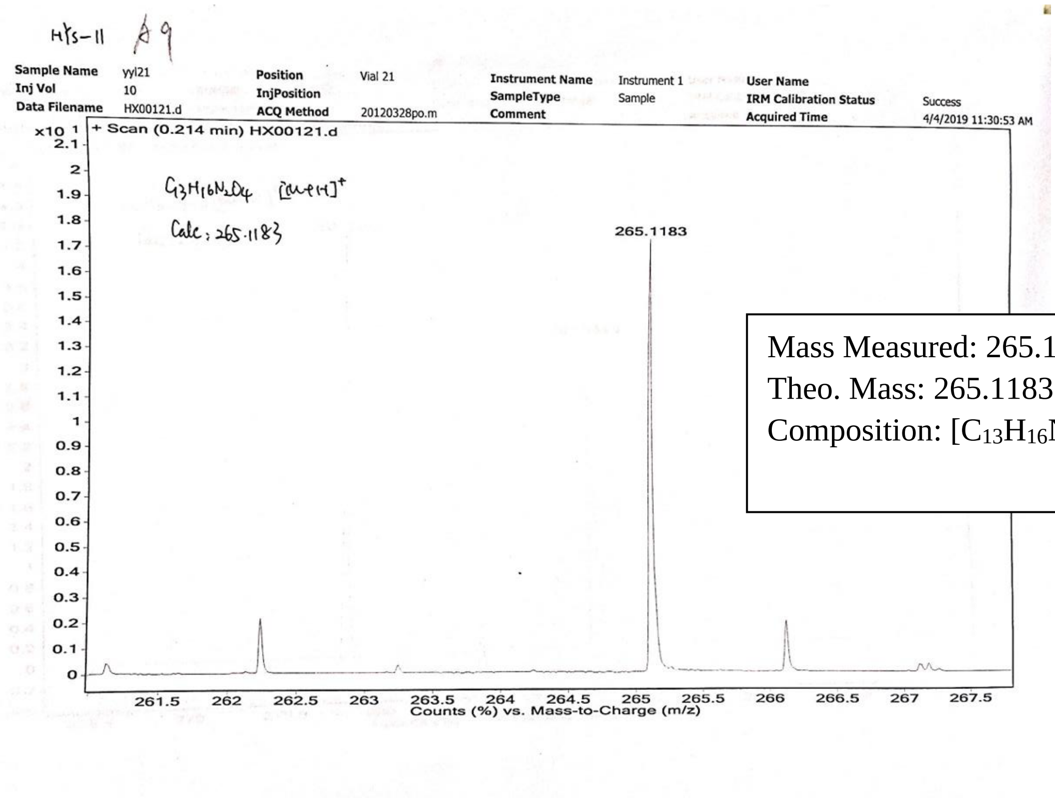

Mass Measured: 265.1183
Theo. Mass: 265.1183
Composition: $[C_{13}H_{16}N_2O_4]^+$

^1H,^{13}C NMR of compound **A10**

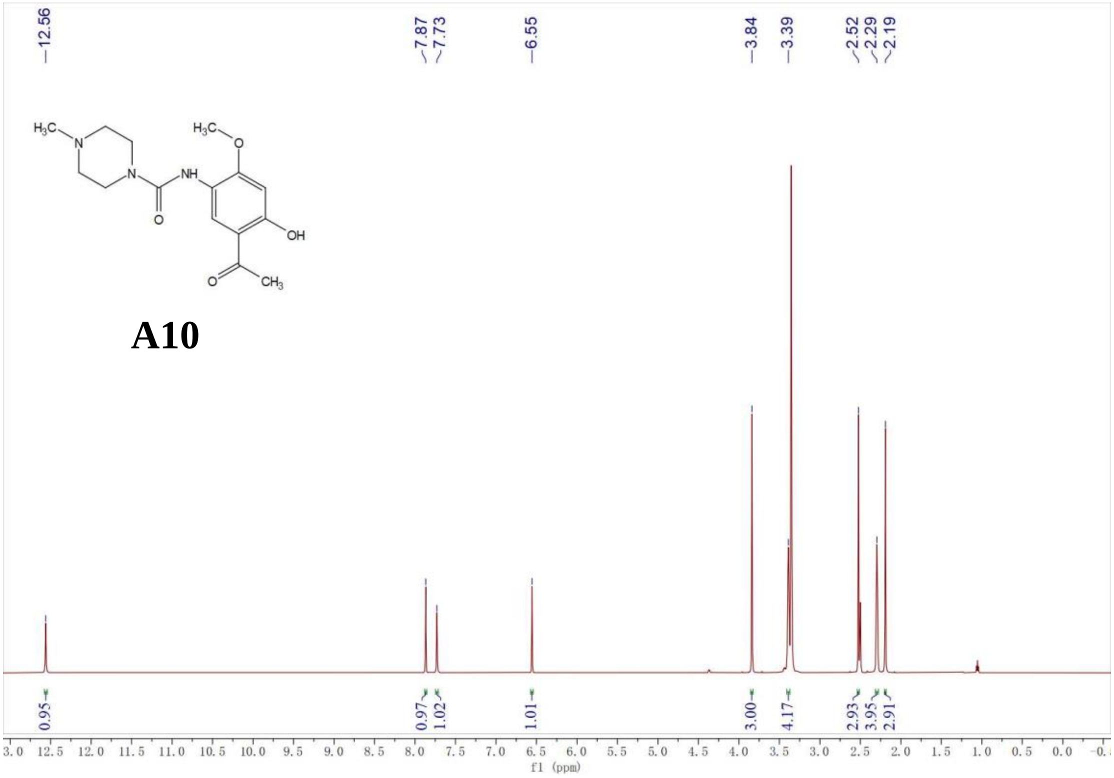

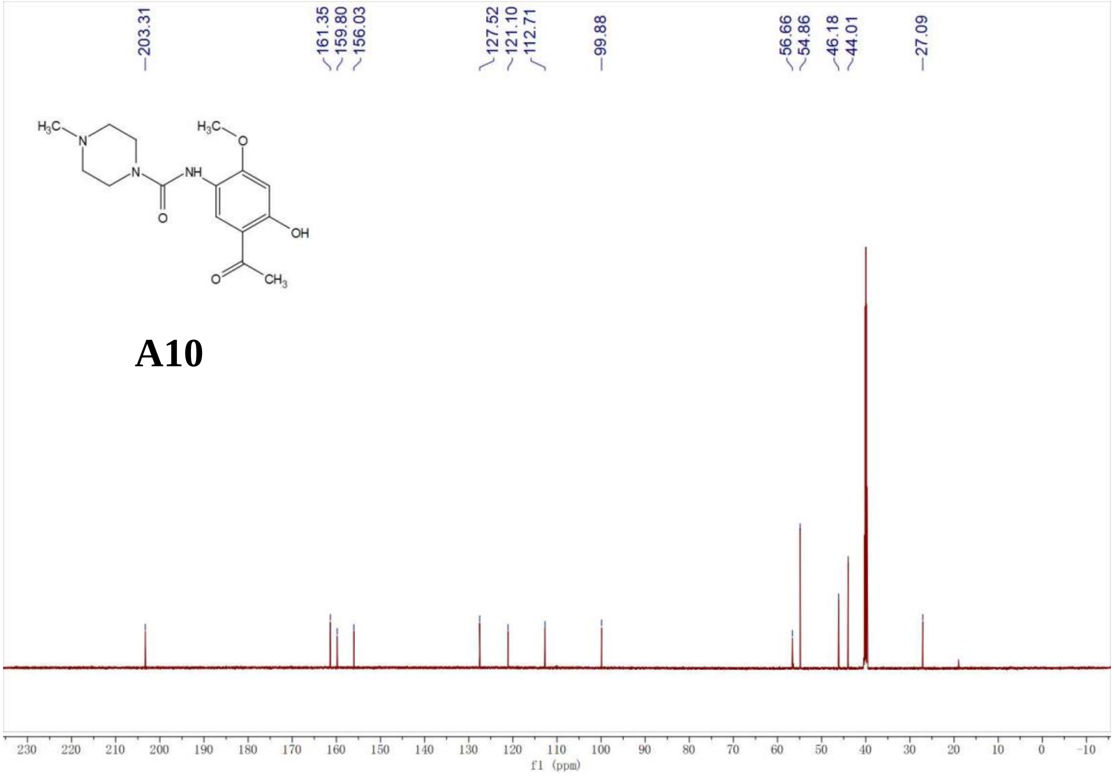

HRMS of compound **A10**

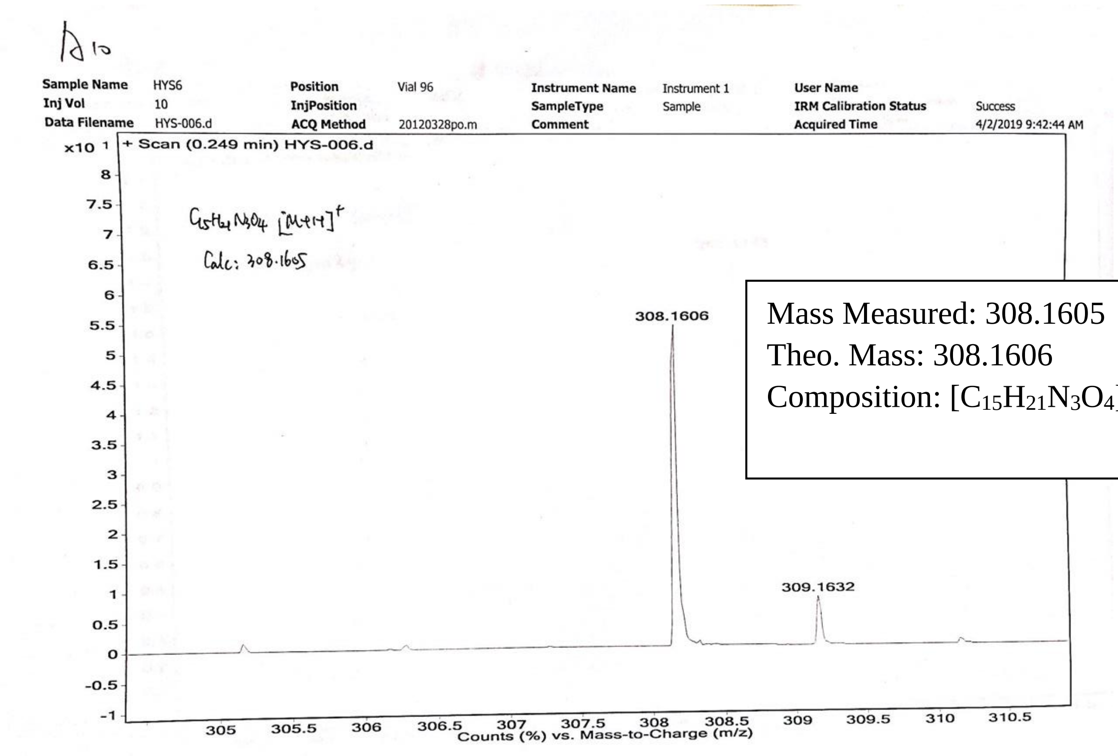

Mass Measured: 308.1605
Theo. Mass: 308.1606
Composition: $[C_{15}H_{21}N_3O_4]^+$

^{1}H,^{13}C NMR of compound **A11**

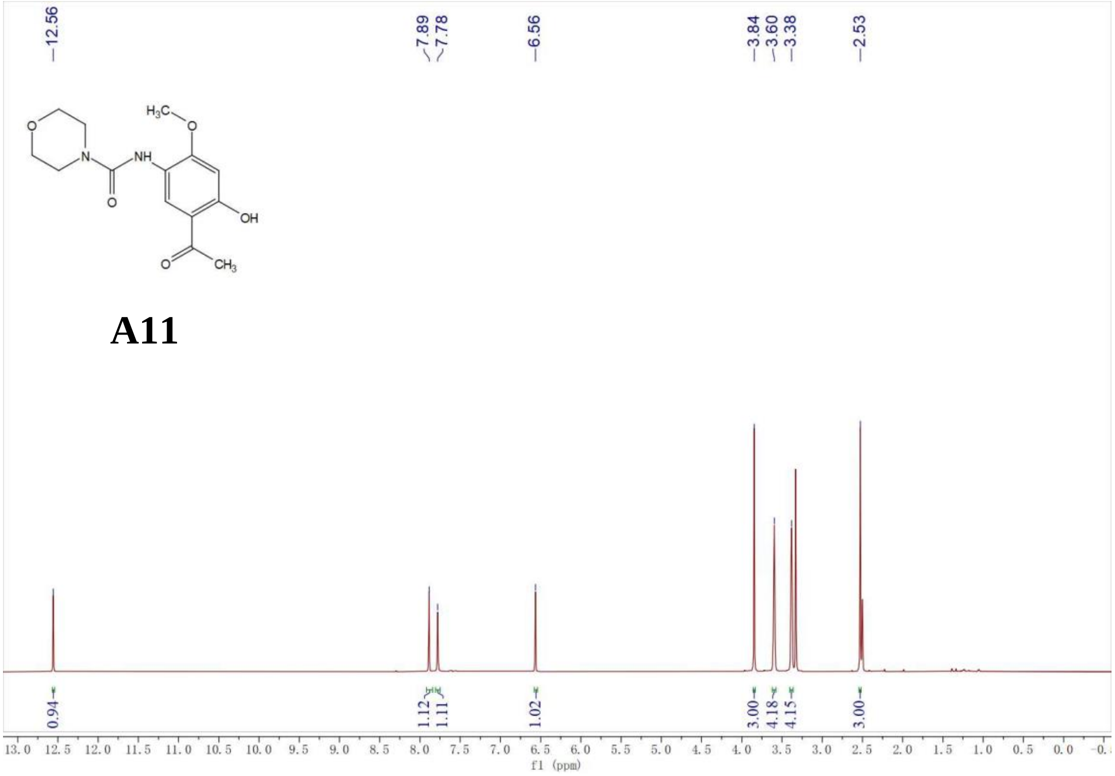

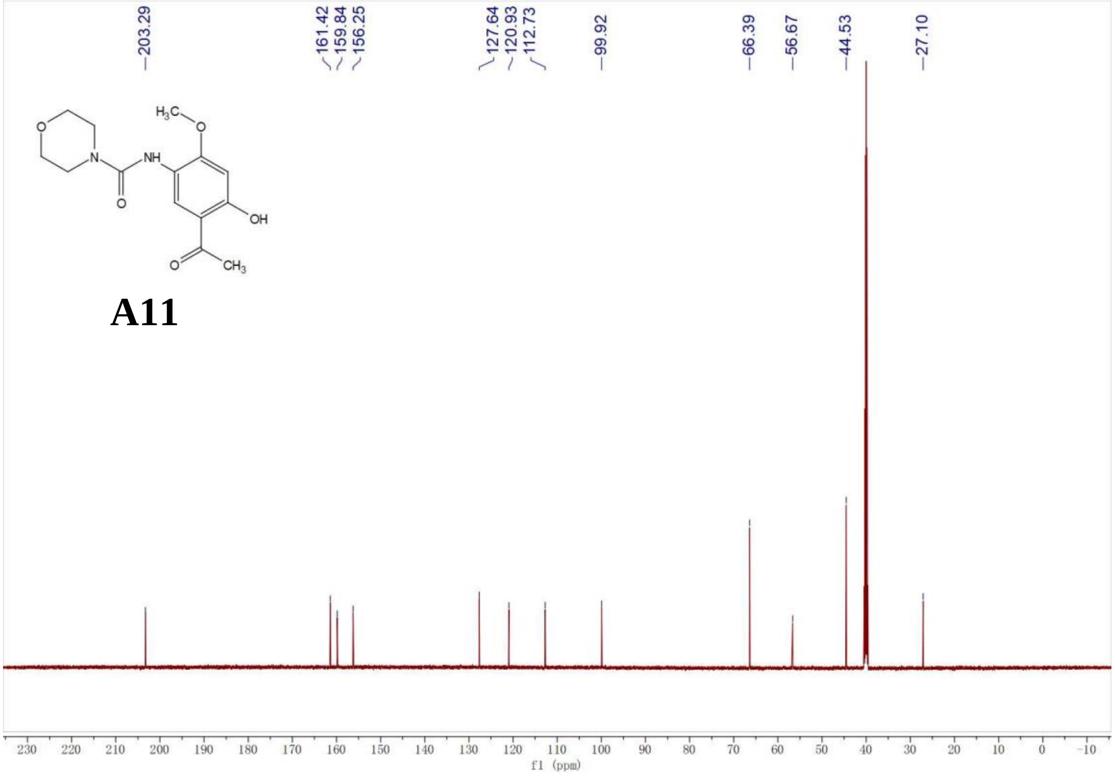

HRMS of compound **A11**

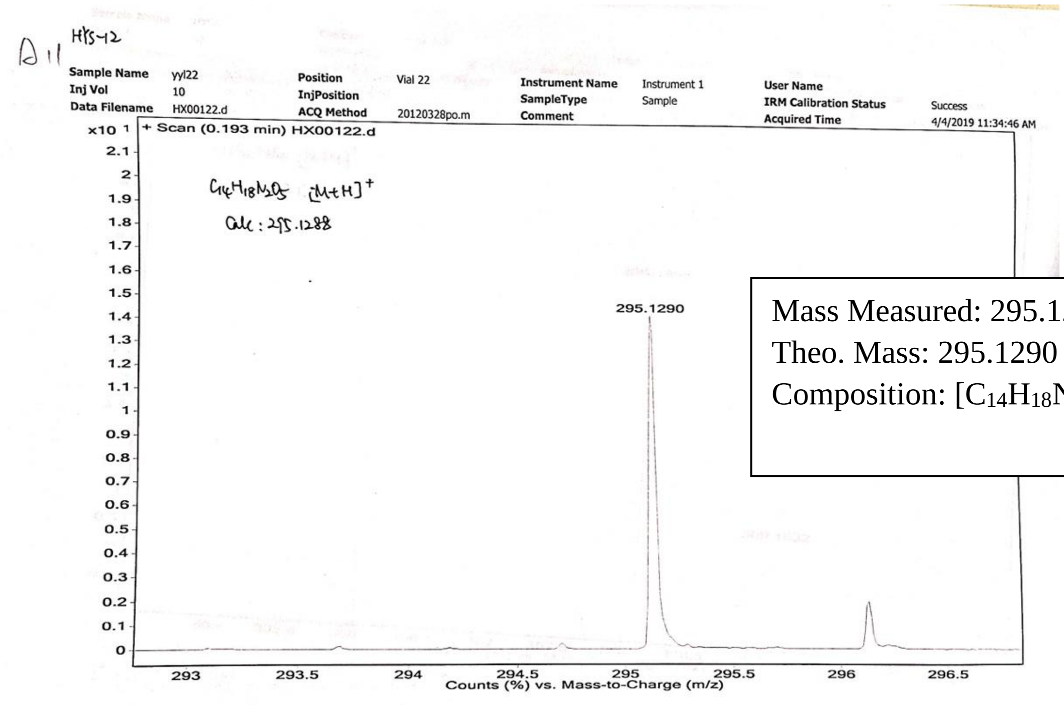

Mass Measured: 295.1288
Theo. Mass: 295.1290
Composition: $[C_{14}H_{18}N_2O_5]^+$

^{1}H,^{13}C NMR of compound **A12**

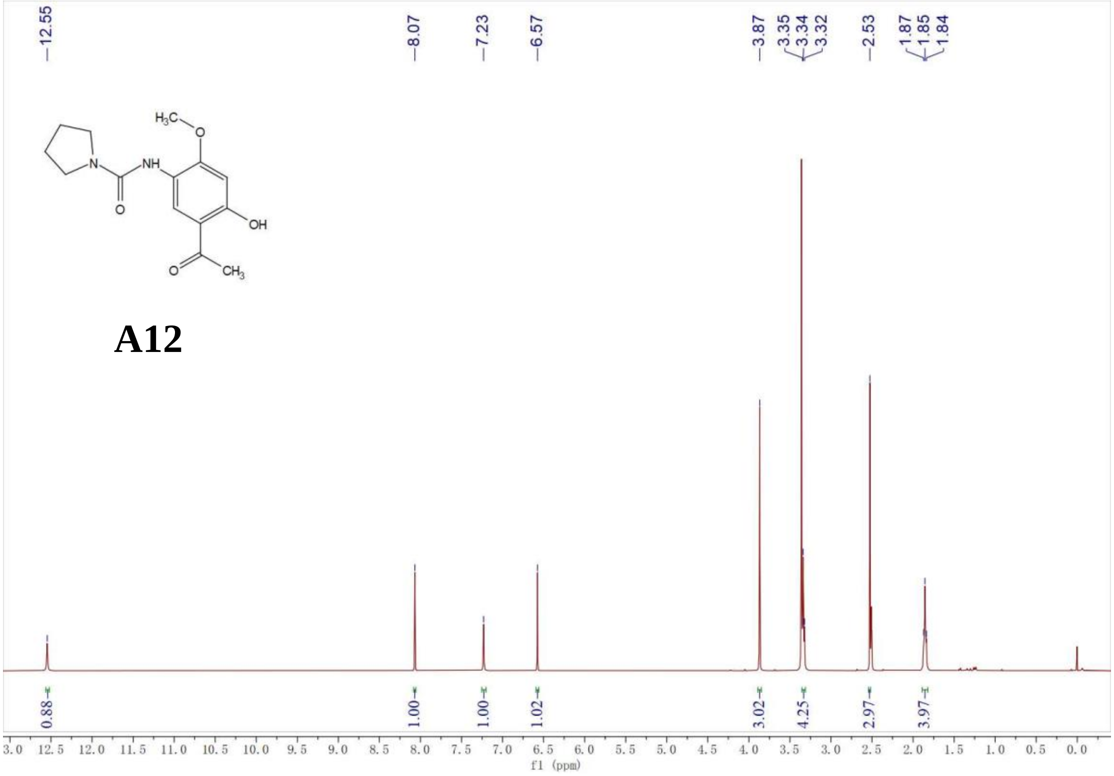

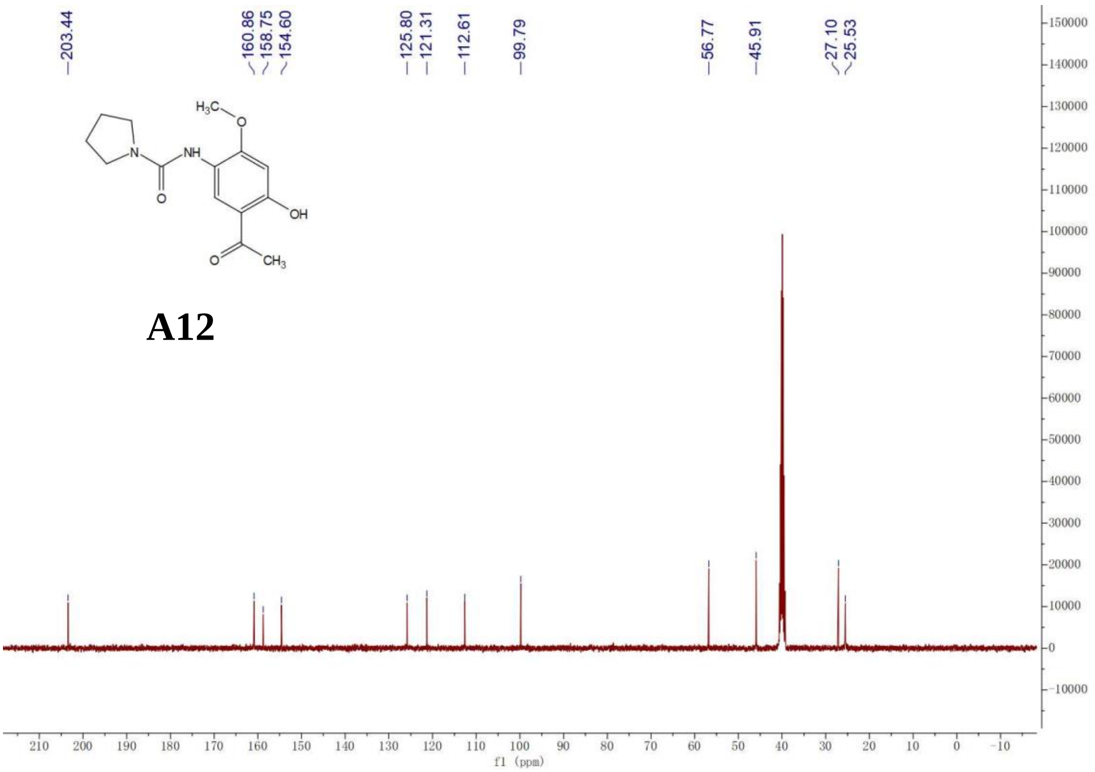

A12

HRMS of compound **A12**

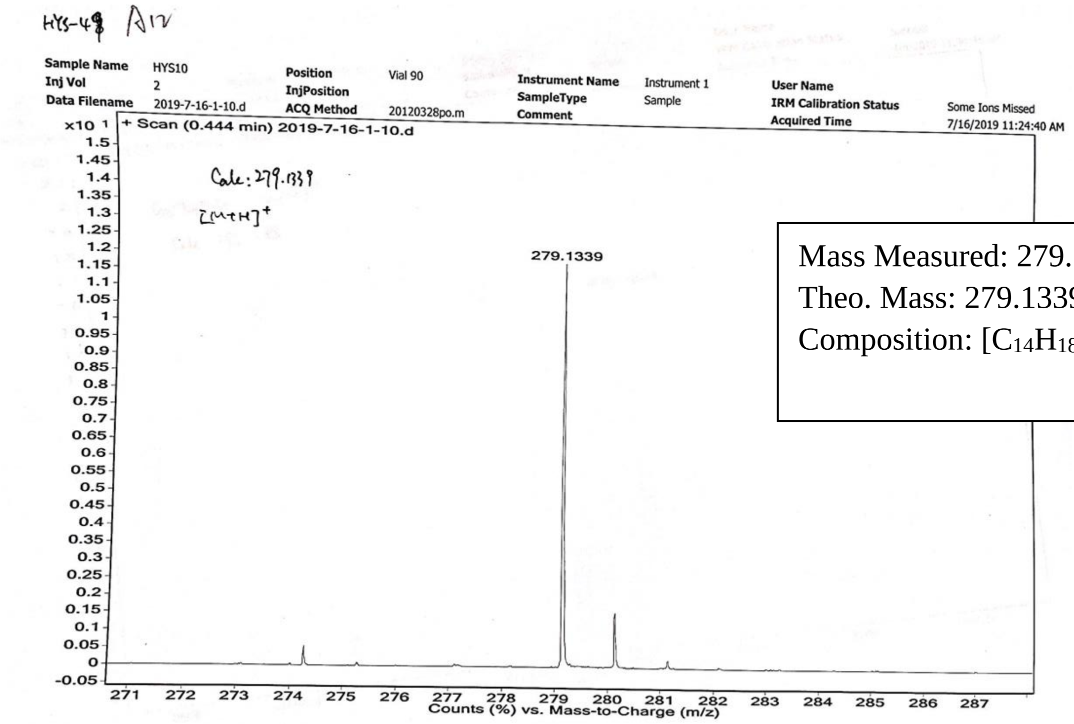

Mass Measured: 279.1339
Theo. Mass: 279.1339
Composition: $[C_{14}H_{18}N_2O_4]^+$

^{1}H,^{13}C NMR of compound **A13**

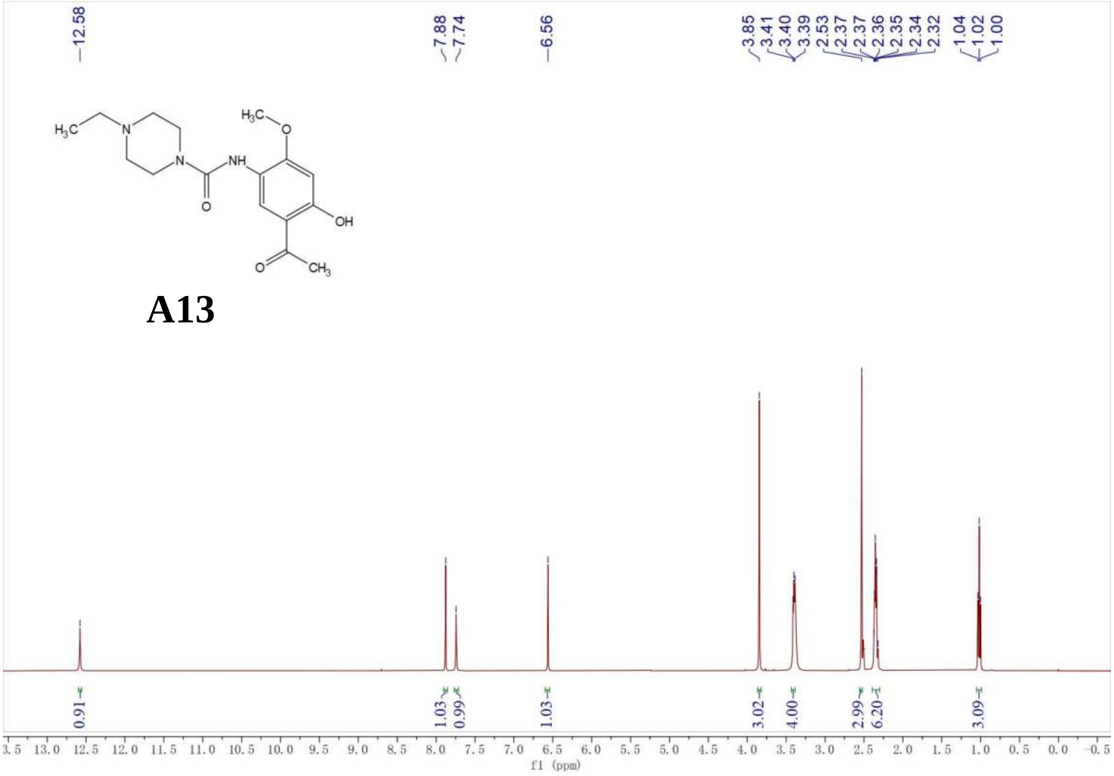

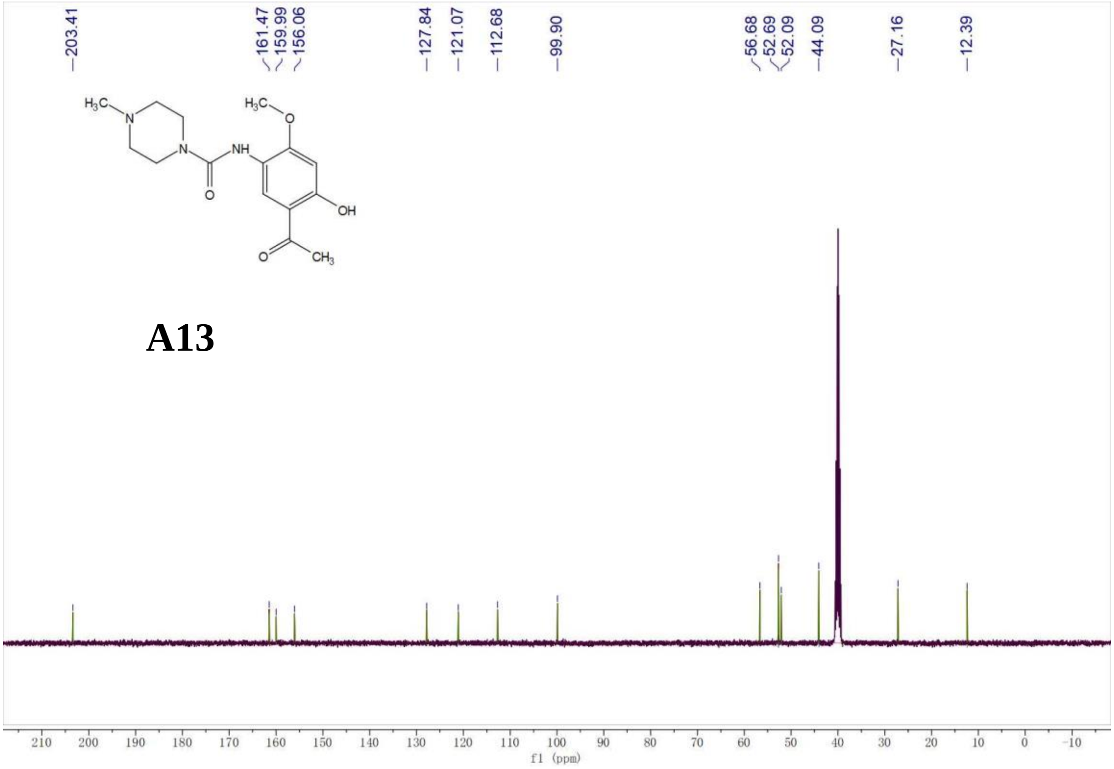

HRMS of compound **A13**

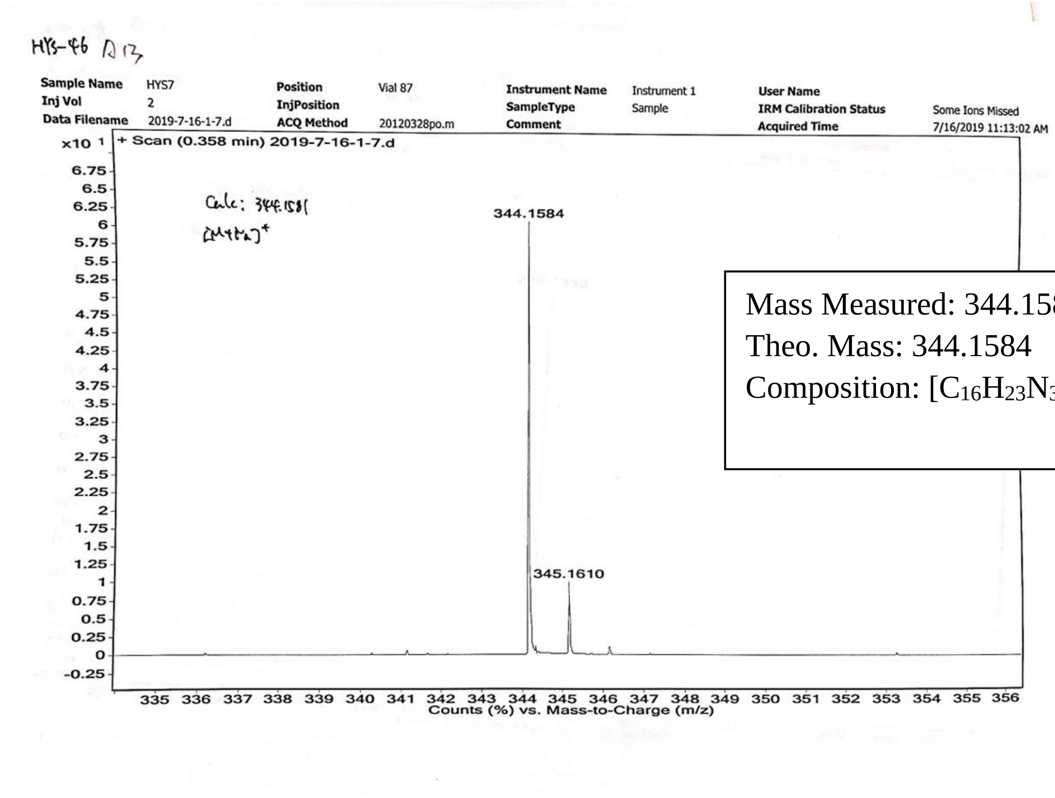

Mass Measured: 344.1581
Theo. Mass: 344.1584
Composition: $[C_{16}H_{23}N_3O_4]^+$

^{1}H,^{13}C NMR of compound **A14**

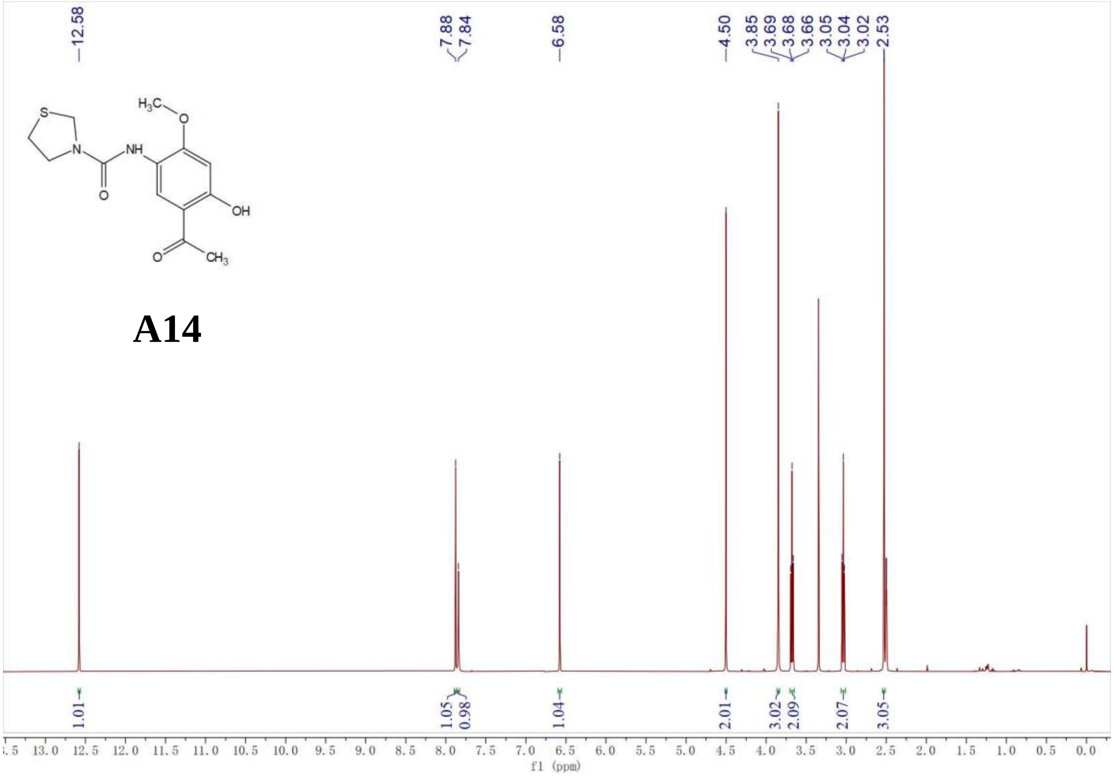

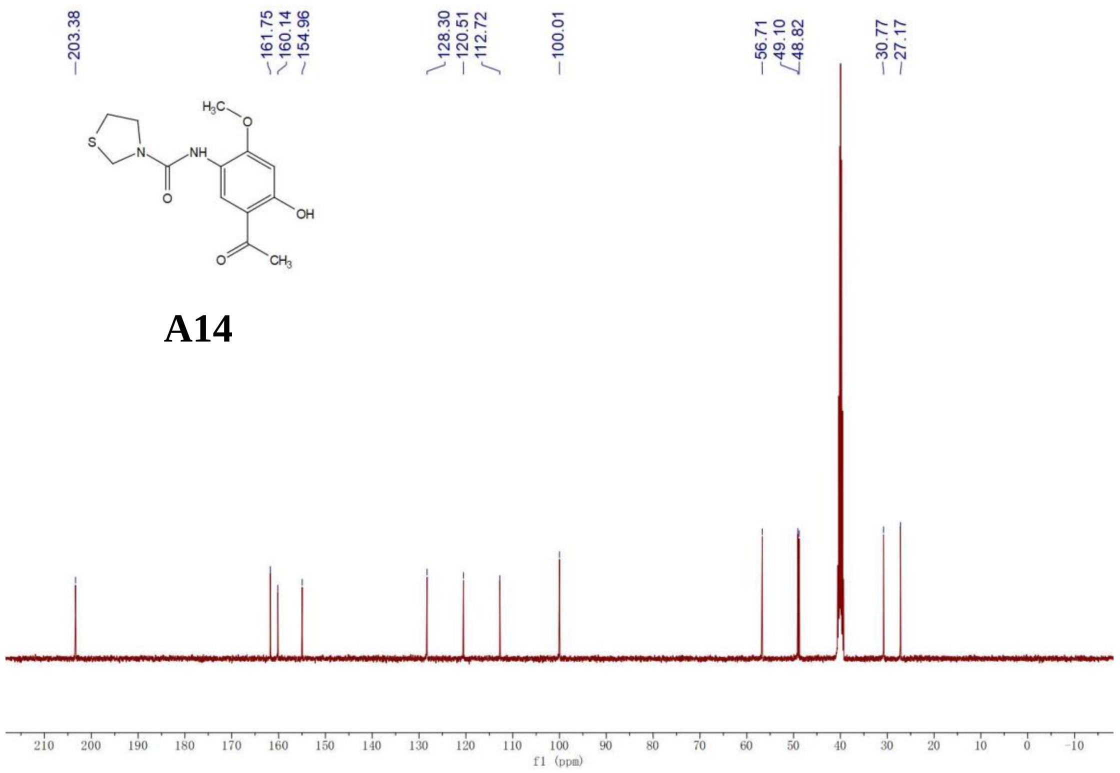

A14

HRMS of compound **A14**

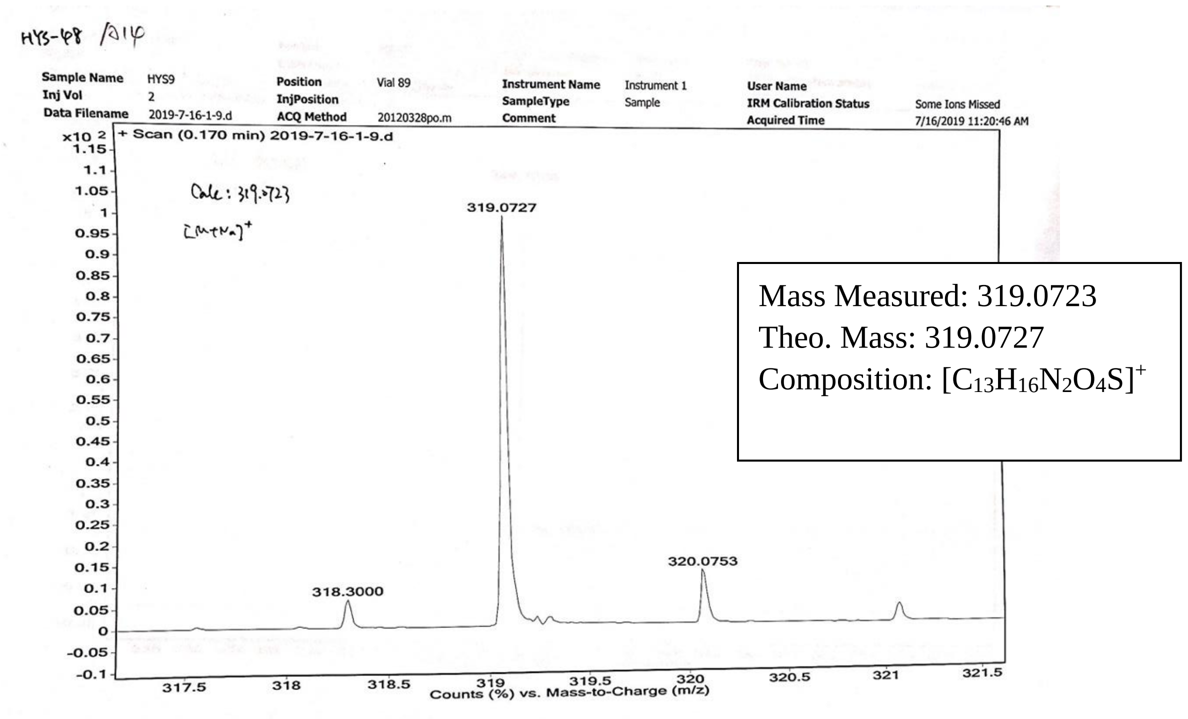

^{1}H,^{13}C NMR of compound **B1**

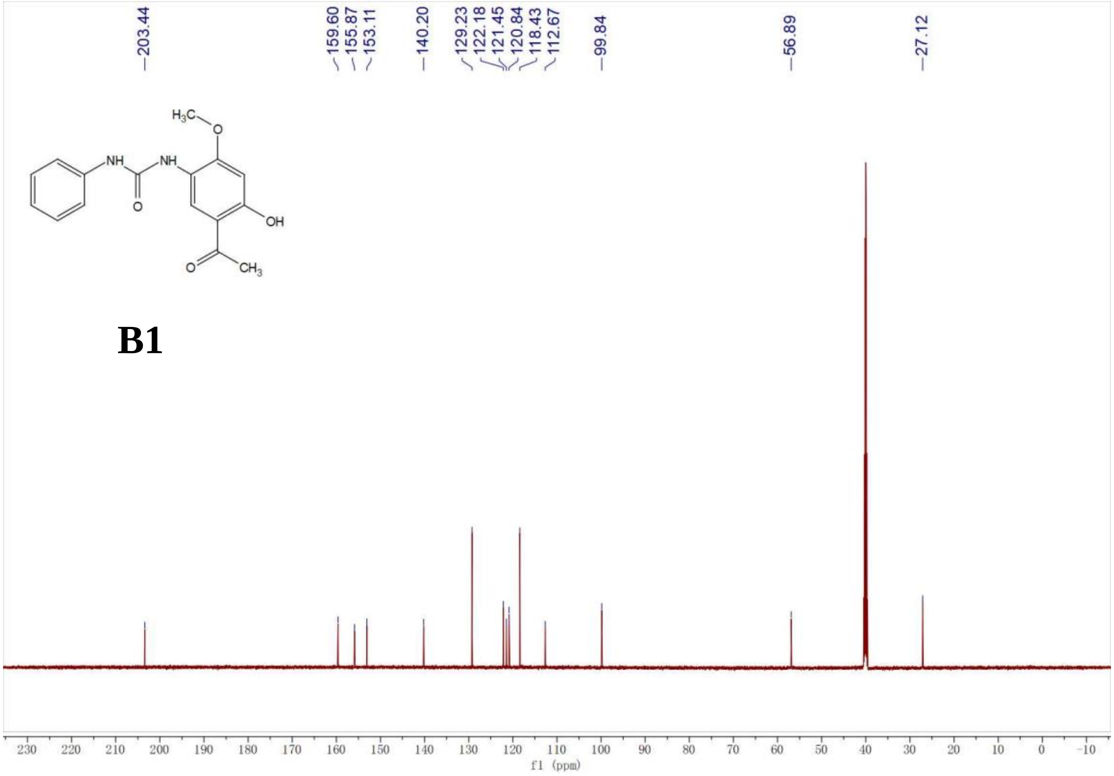

HRMS of compound **B1**

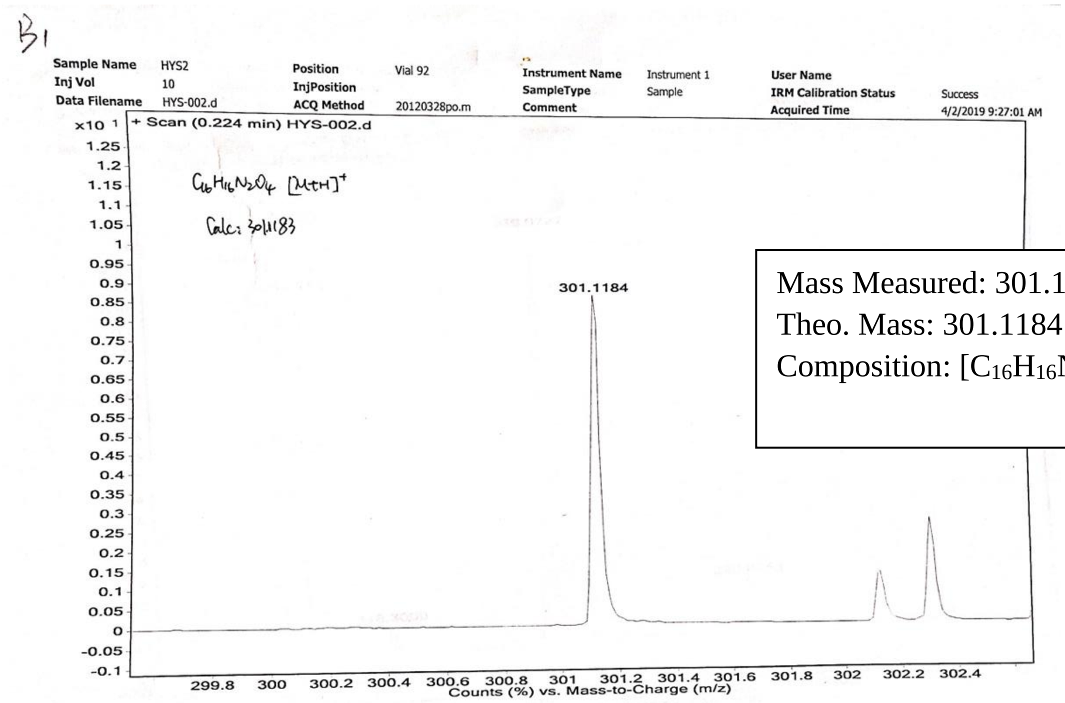

Mass Measured: 301.1183
Theo. Mass: 301.1184
Composition: $[C_{16}H_{16}N_2O_4]^+$

^{1}H,^{13}C NMR of compound **B2**

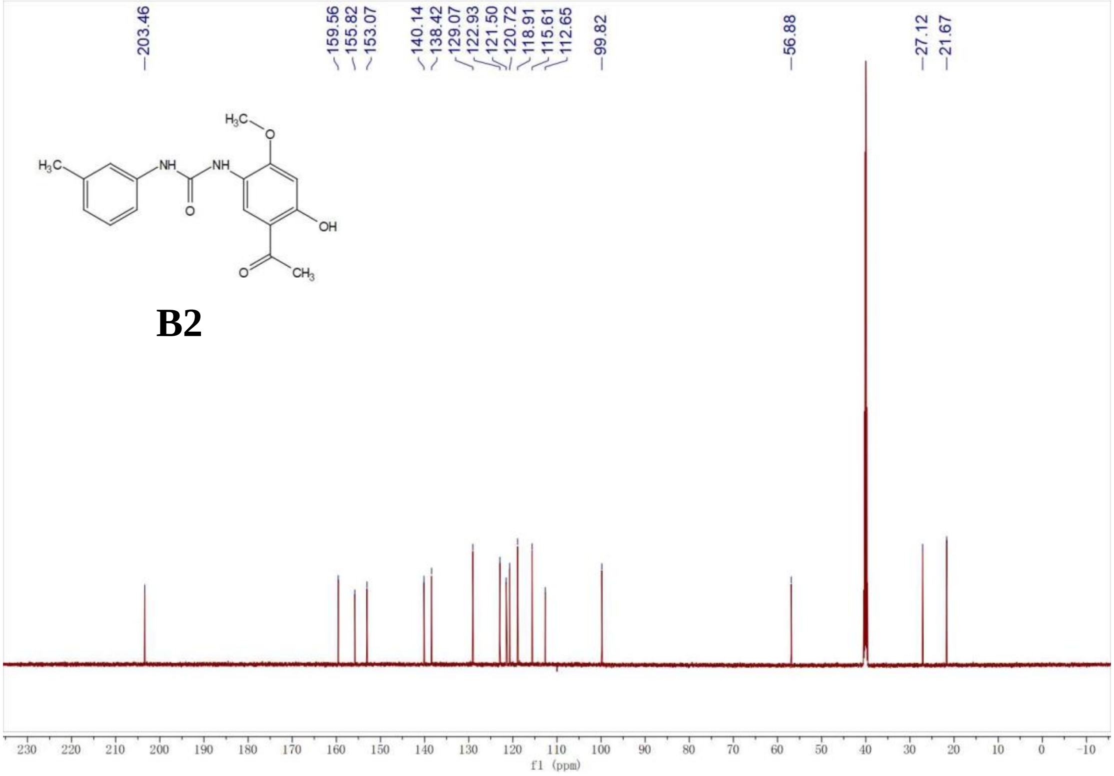

HRMS of compound **B2**

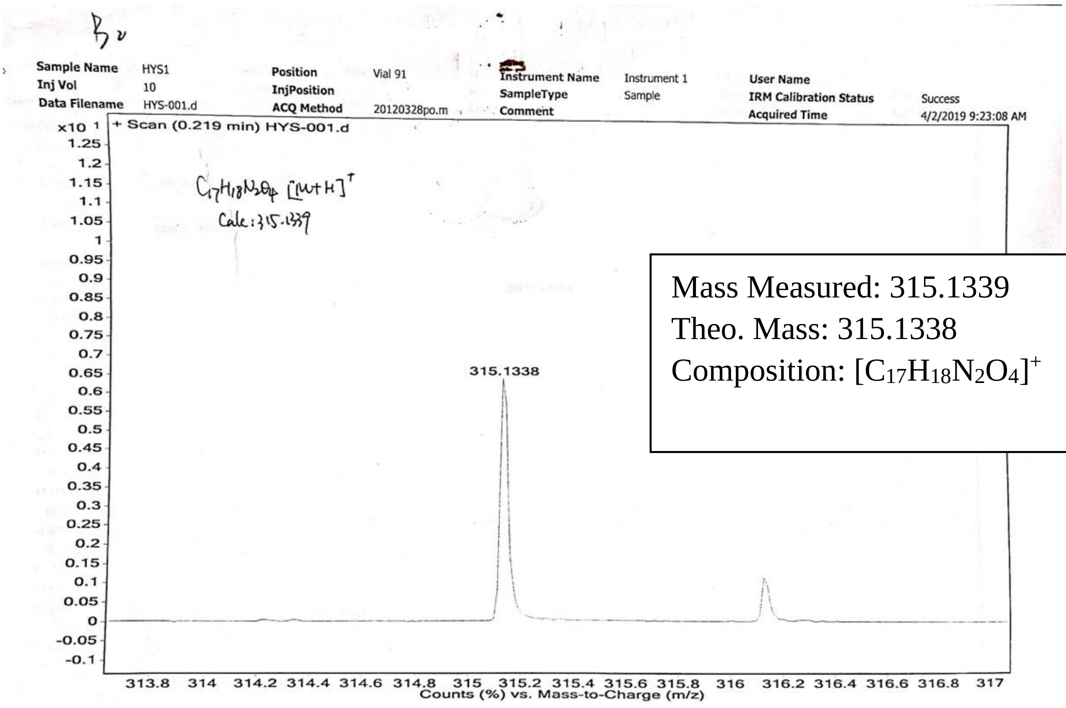

Mass Measured: 315.1339
Theo. Mass: 315.1338
Composition: $[C_{17}H_{18}N_2O_4]^+$

^{1}H,^{13}C NMR of compound **B3**

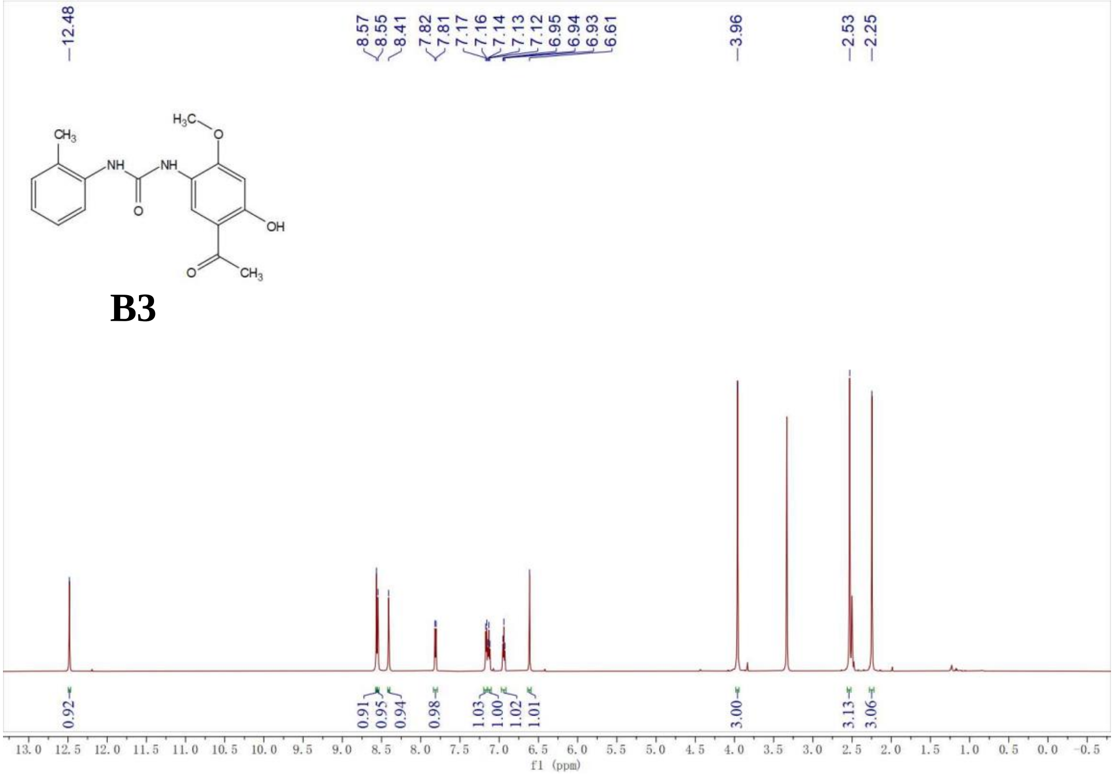

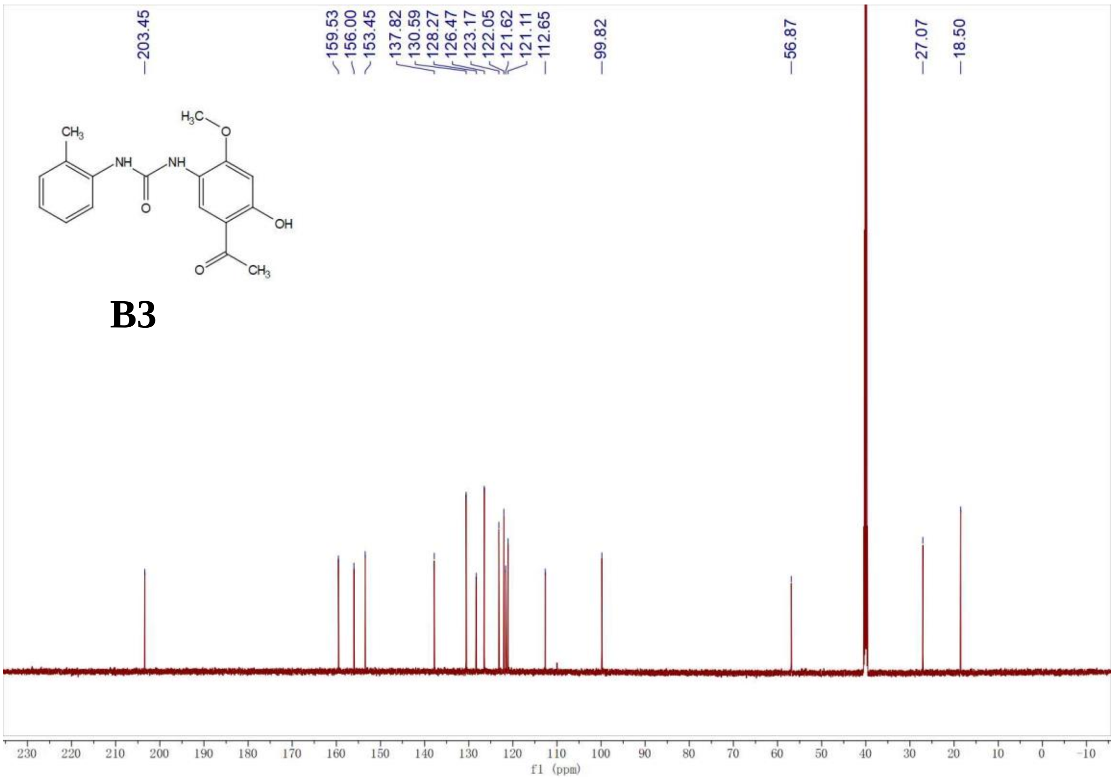

HRMS of compound **B3**

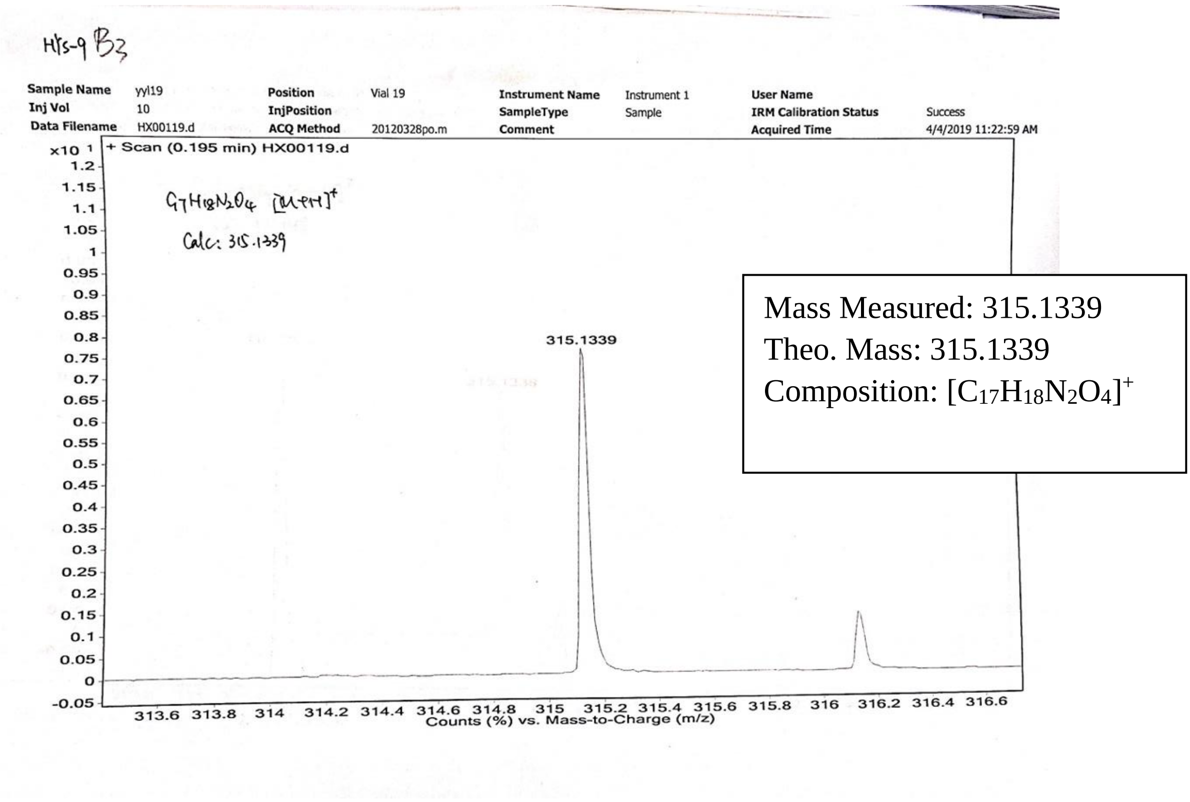

Mass Measured: 315.1339
Theo. Mass: 315.1339
Composition: $[C_{17}H_{18}N_2O_4]^+$

^1H,^{13}C NMR of compound **B4**

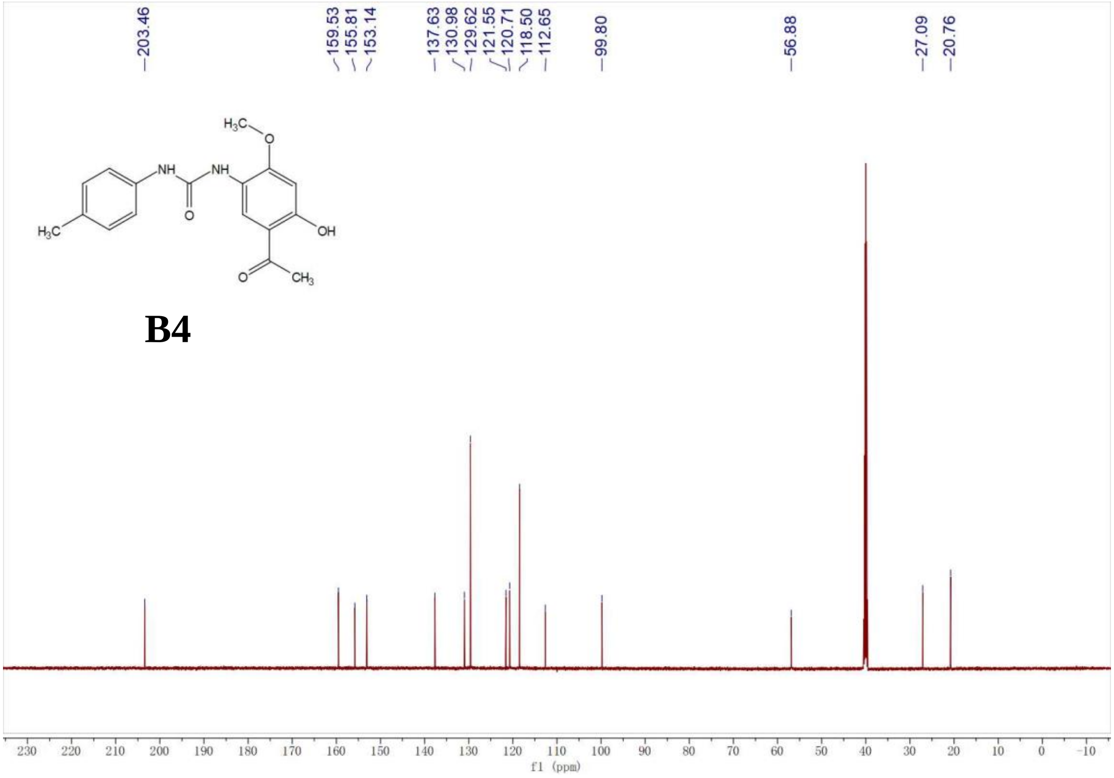

HRMS of compound **B4**

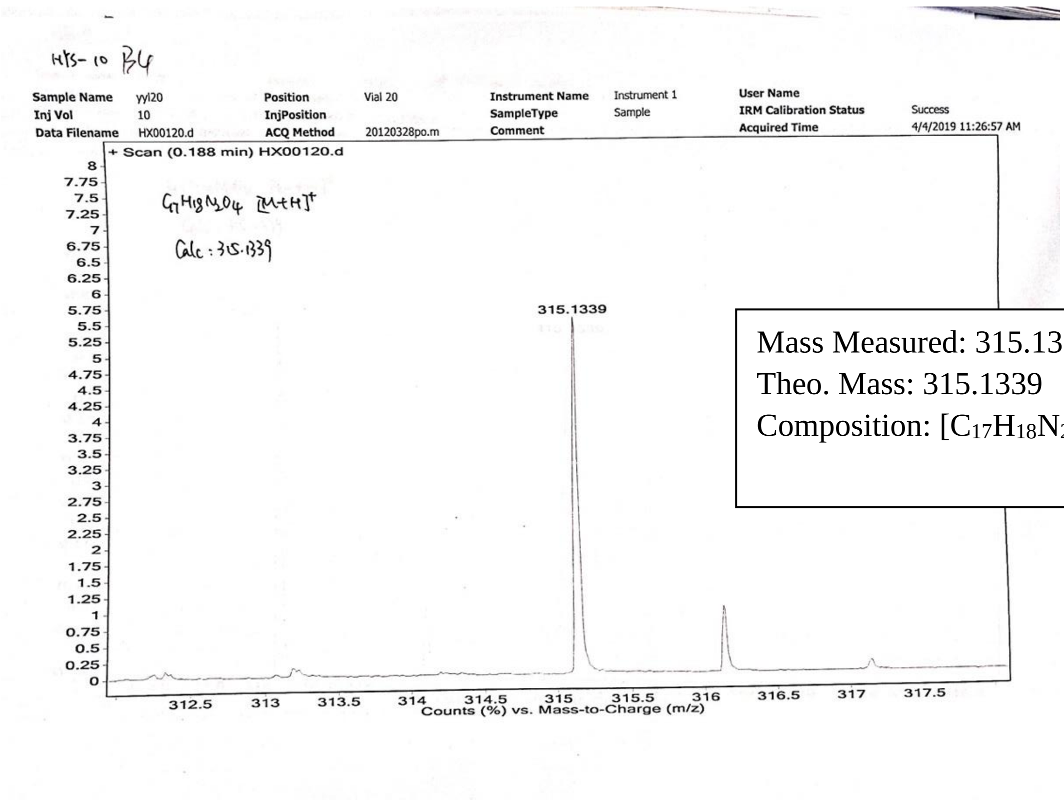

Mass Measured: 315.1339
Theo. Mass: 315.1339
Composition: $[C_{17}H_{18}N_2O_4]^+$

^{1}H,^{13}C NMR of compound **B5**

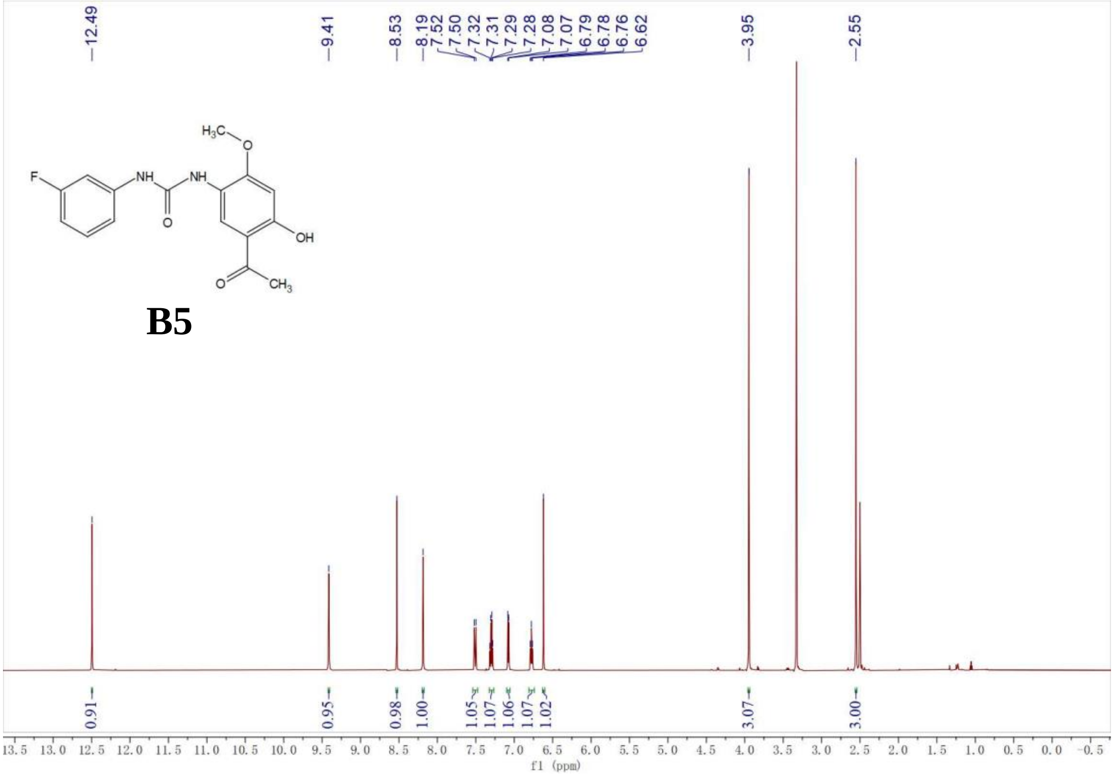

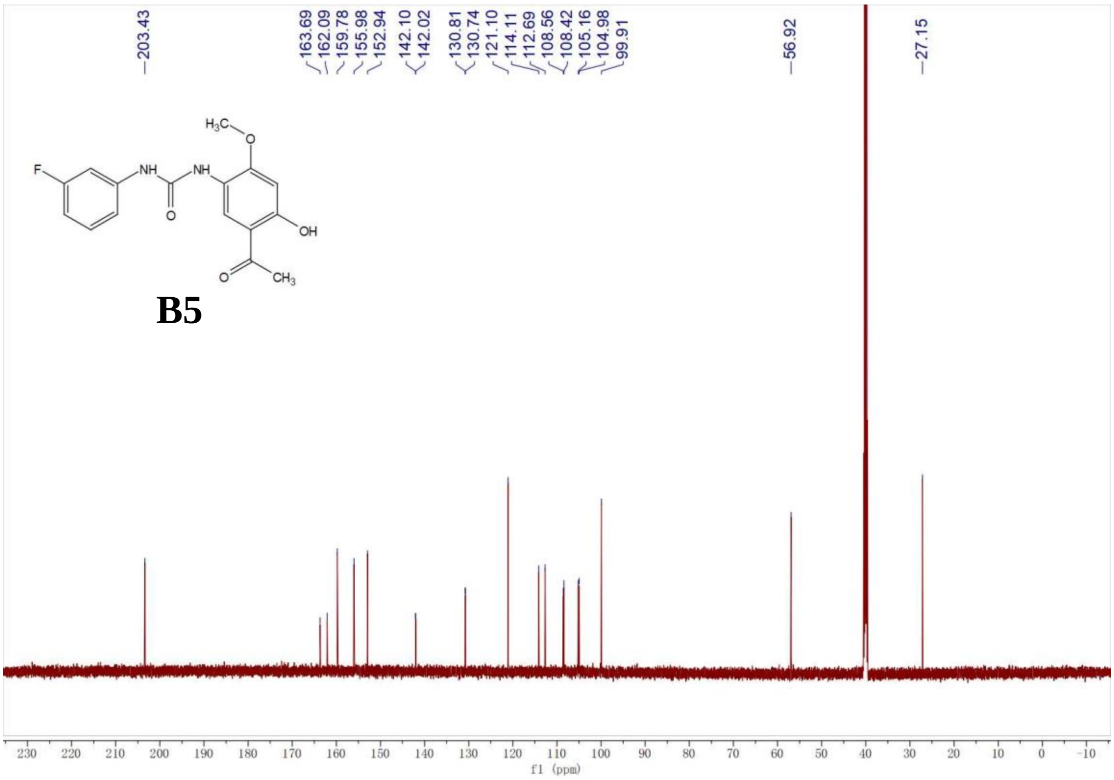

HRMS of compound **B5**

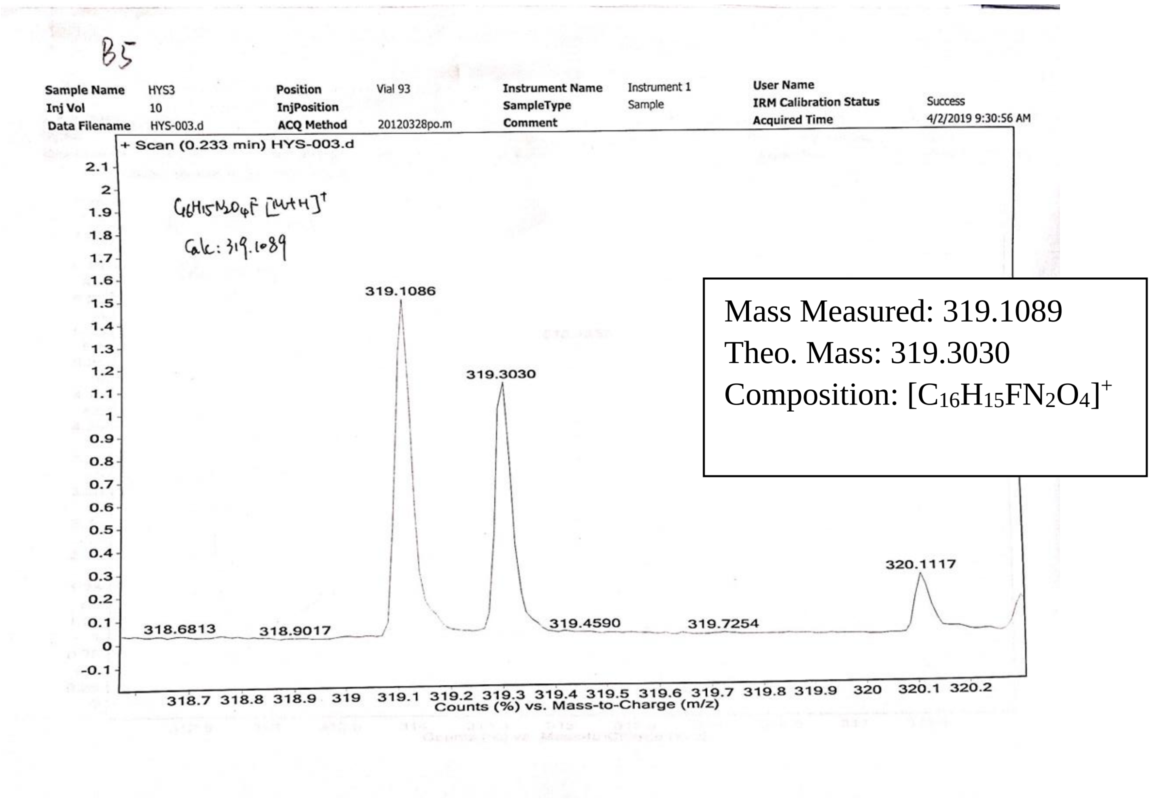

^{1}H,^{13}C NMR of compound **B6**

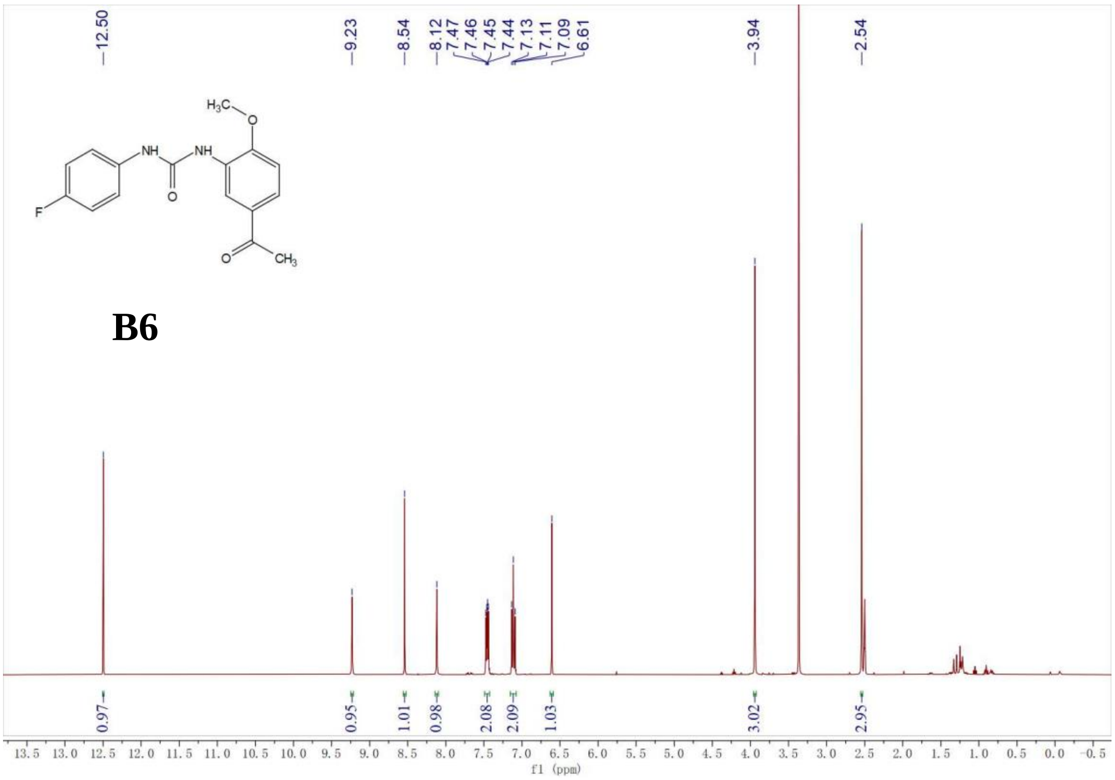

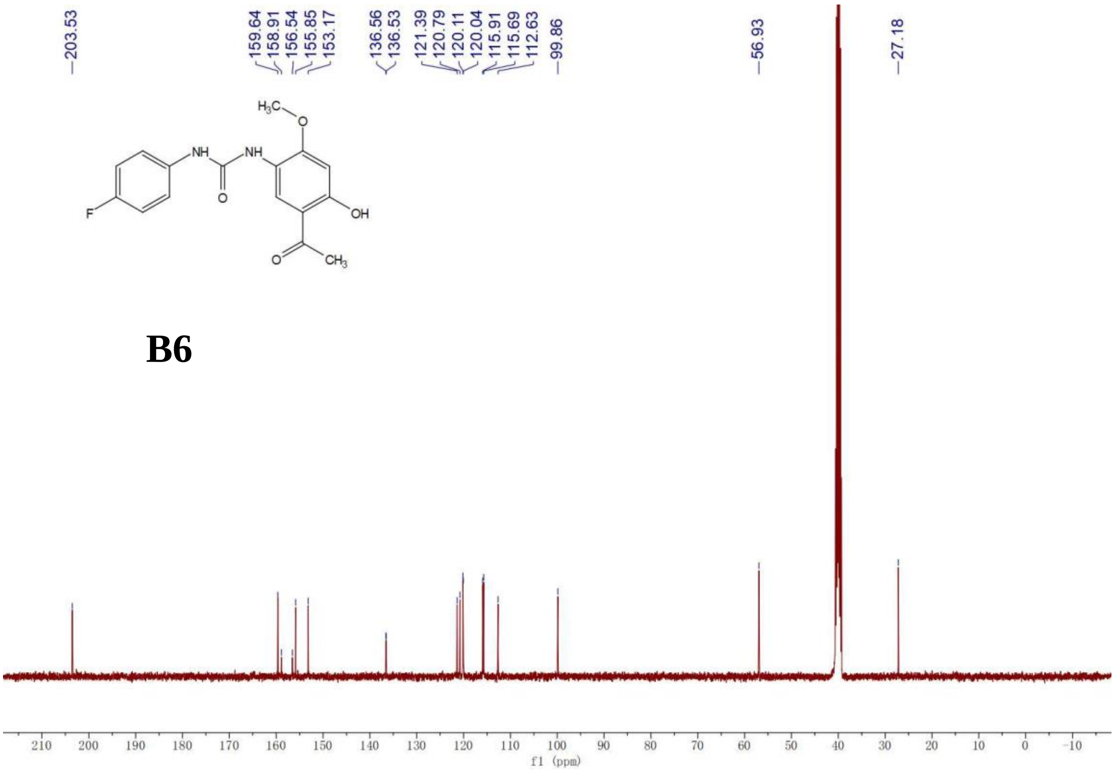

HRMS of compound **B6**

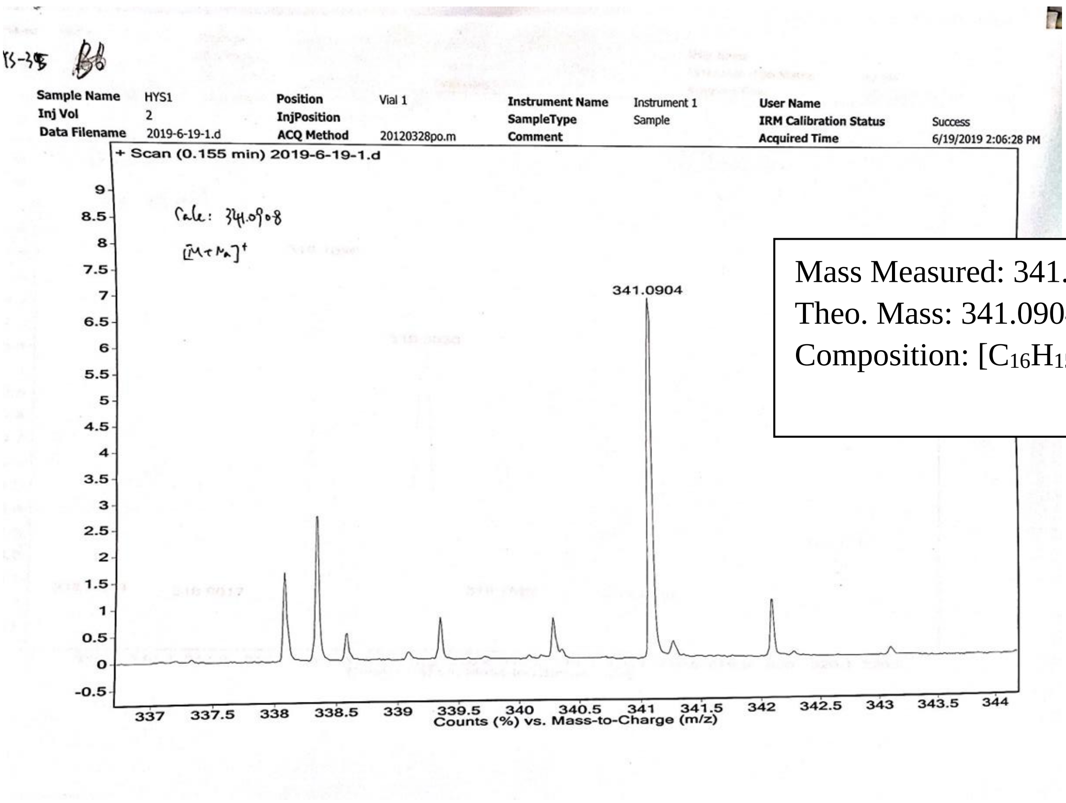

Mass Measured: 341.0908
Theo. Mass: 341.0904
Composition: $[C_{16}H_{15}FN_2O_3]^+$

^{1}H,^{13}C NMR of compound **B7**

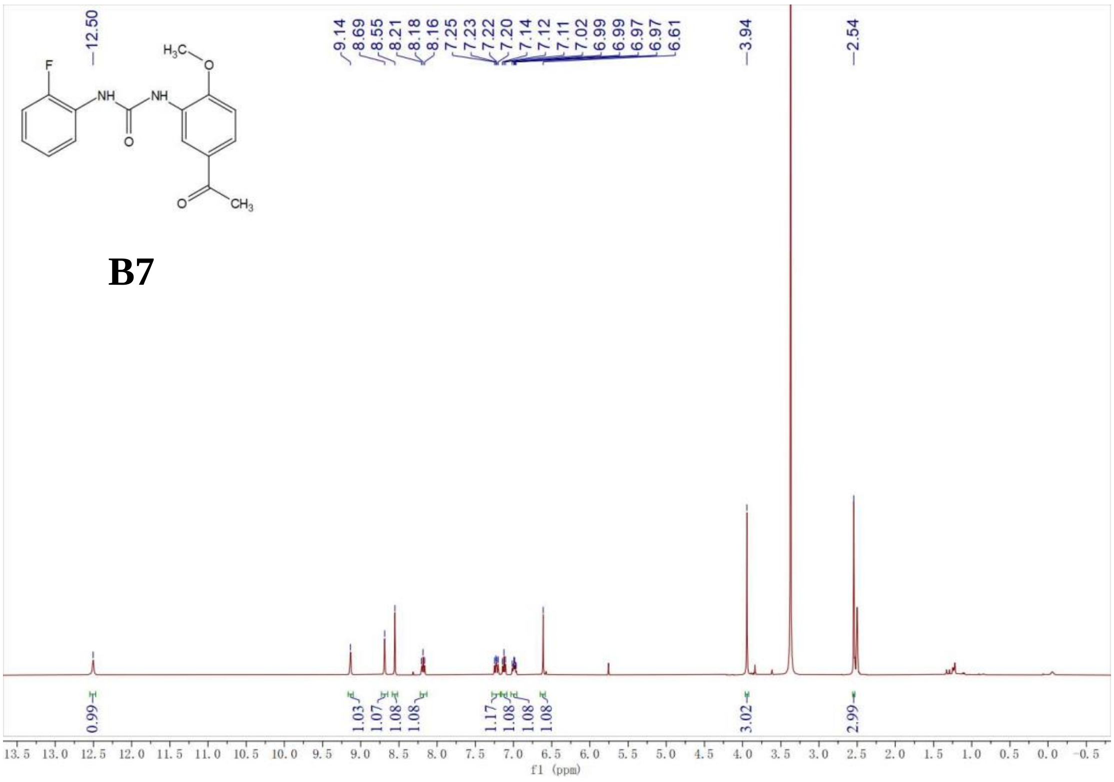

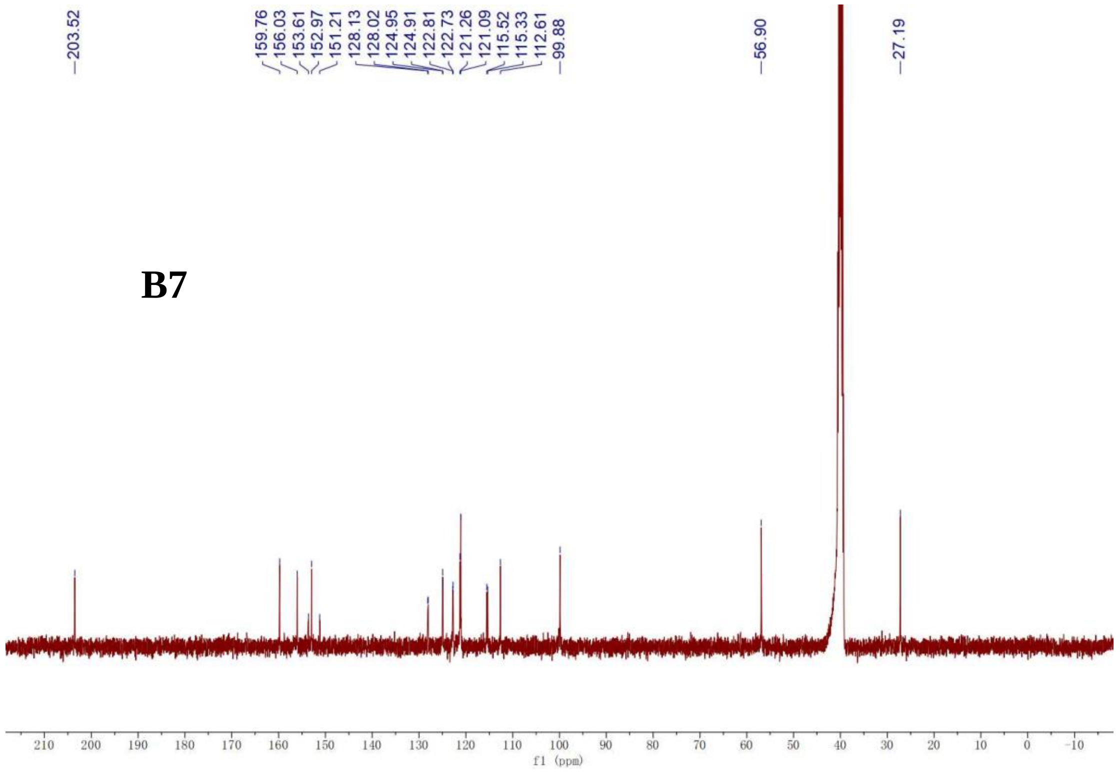

HRMS of compound **B7**

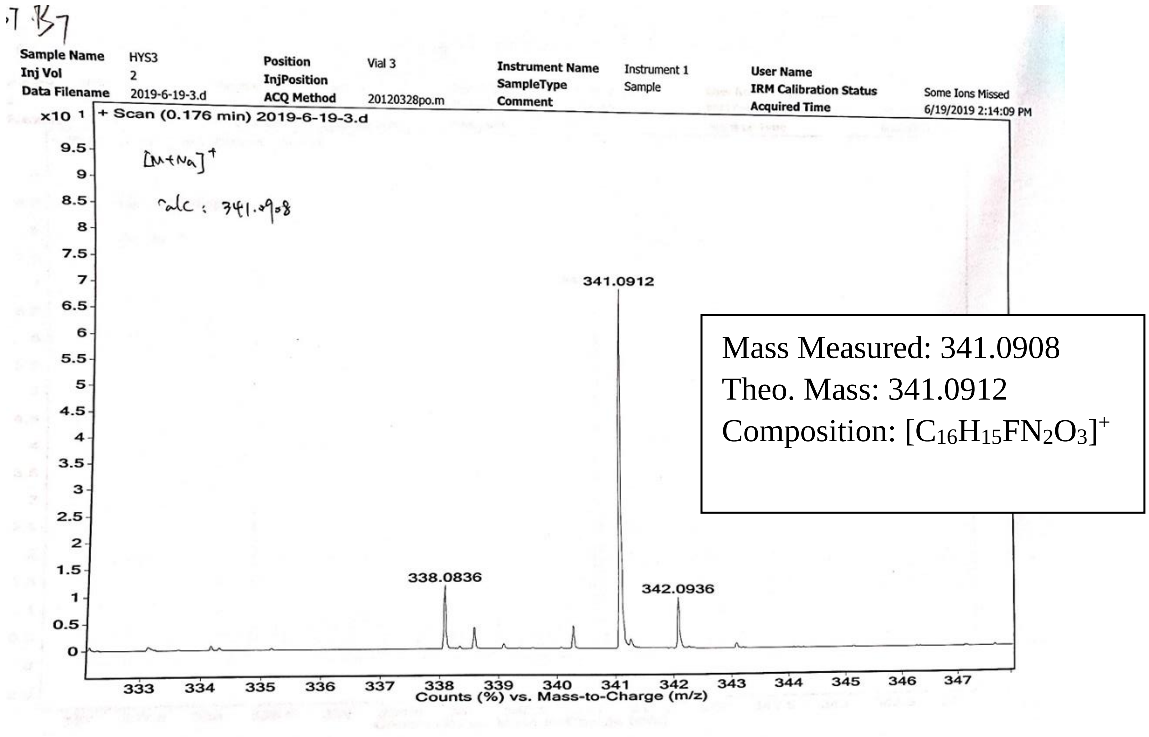

Mass Measured: 341.0908
Theo. Mass: 341.0912
Composition: $[C_{16}H_{15}FN_2O_3]^+$

^1H,^{13}C NMR of compound **B8**

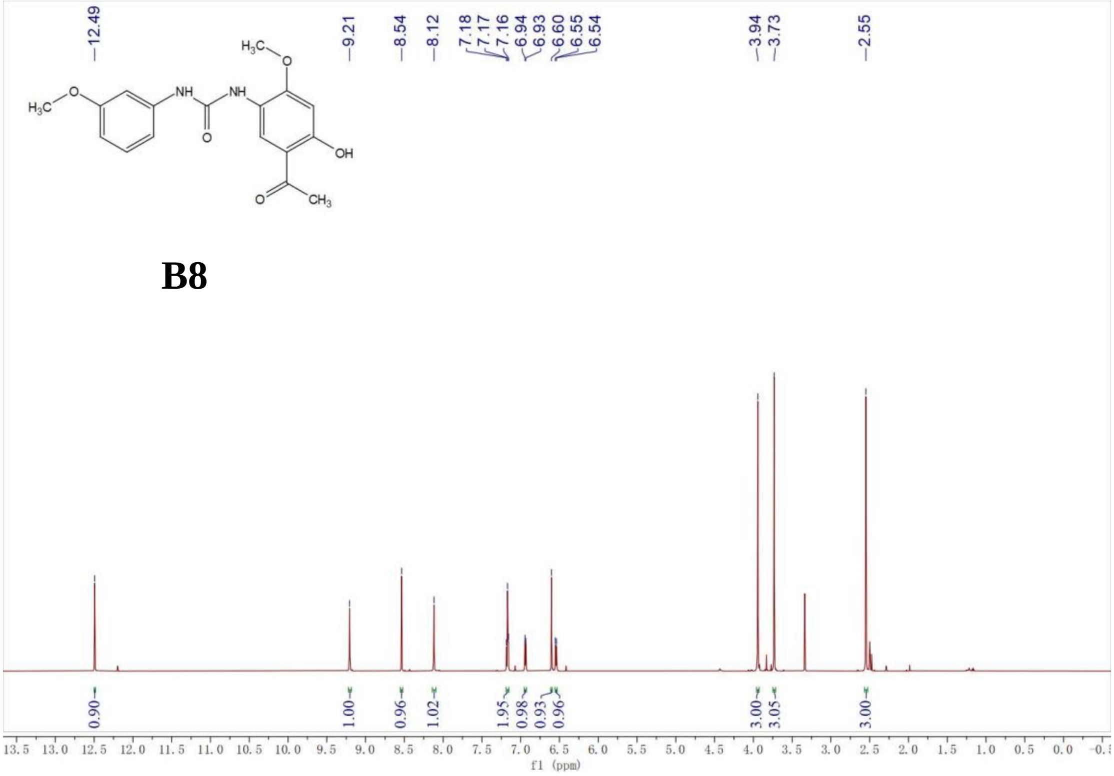

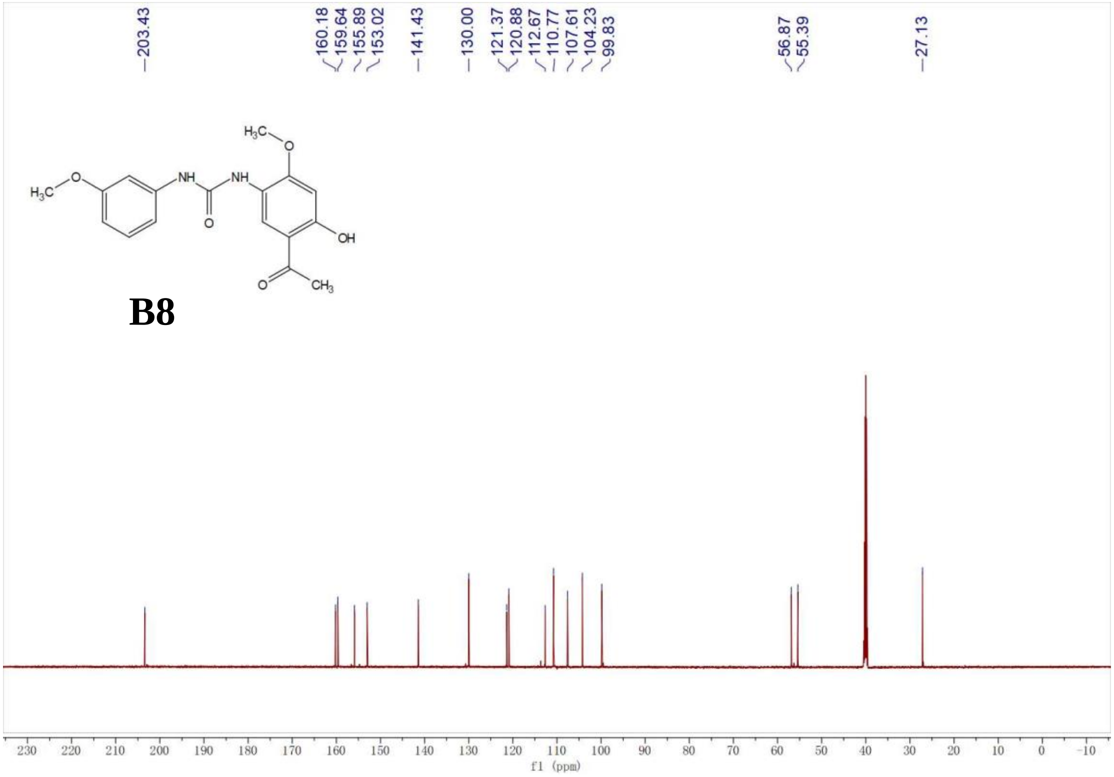

HRMS of compound **B8**

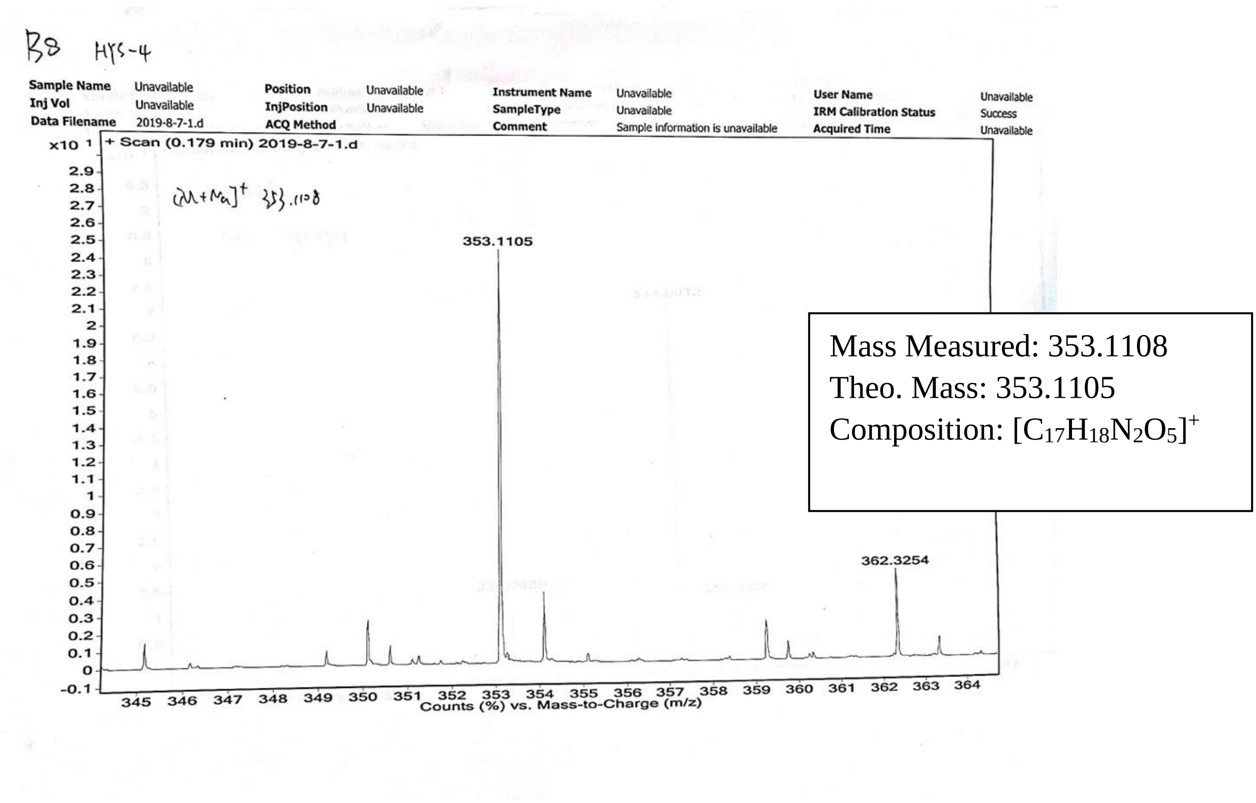

^{1}H,^{13}C NMR of compound **B9**

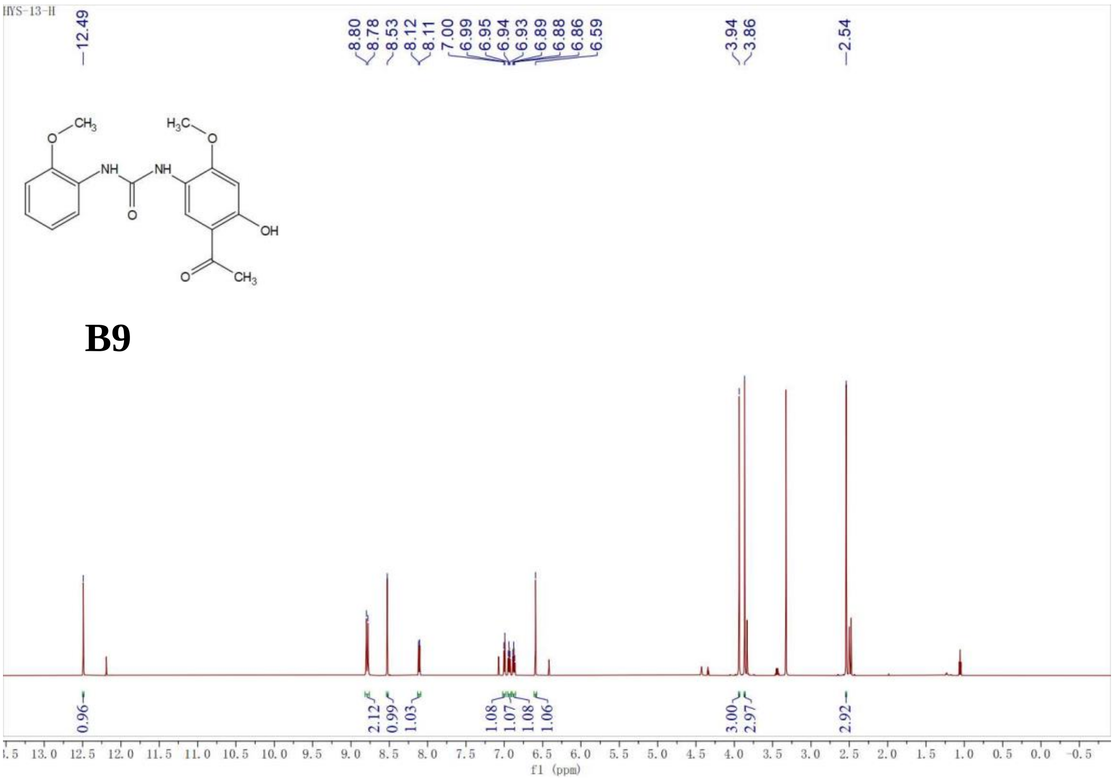

HRMS of compound **B9**

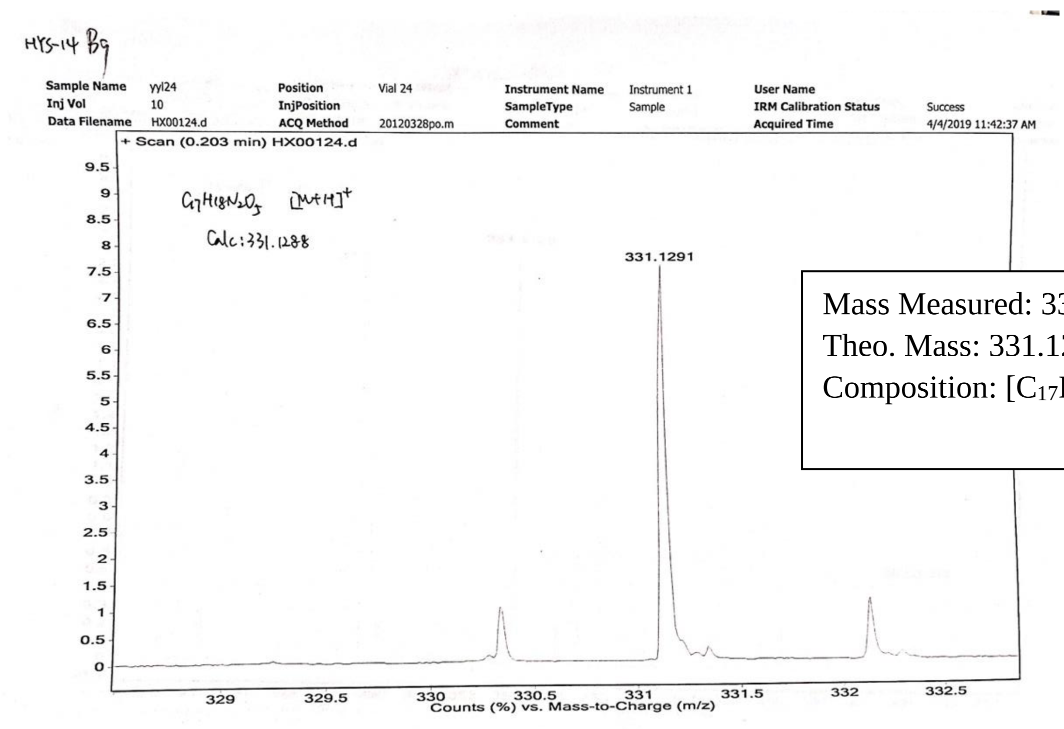

Mass Measured: 331.1288
Theo. Mass: 331.1291
Composition: $[C_{17}H_{18}N_2O_5]^+$

^{1}H,^{13}C NMR of compound **B10**

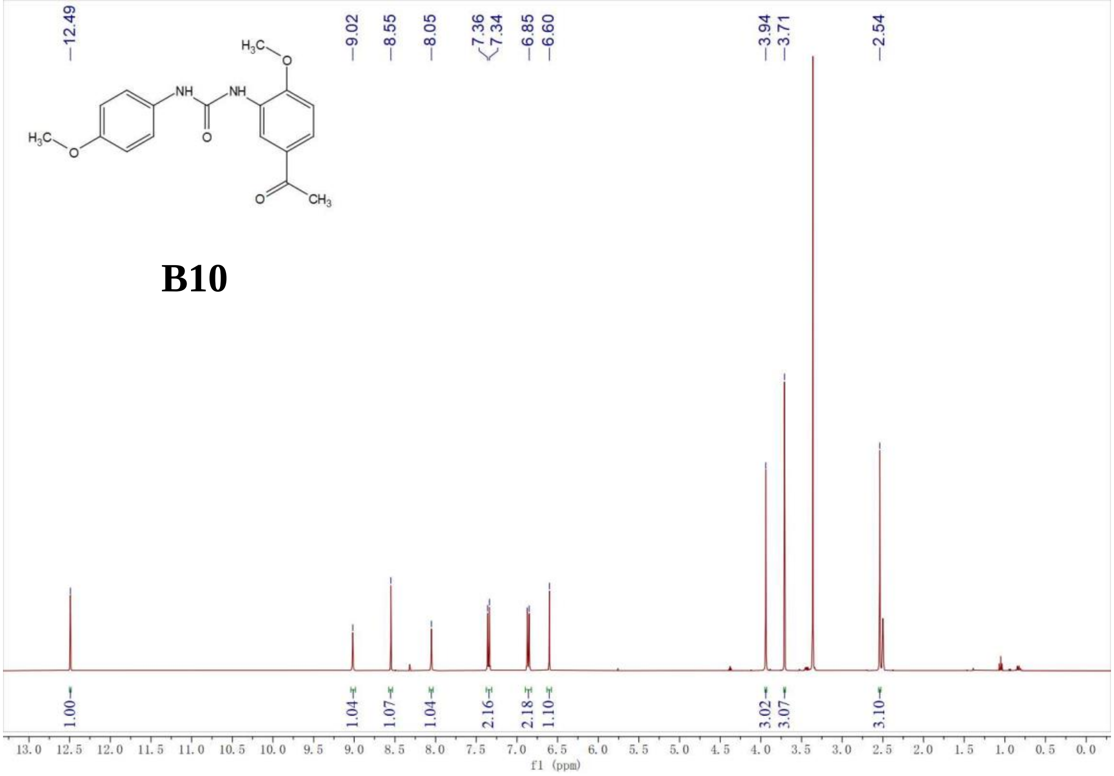

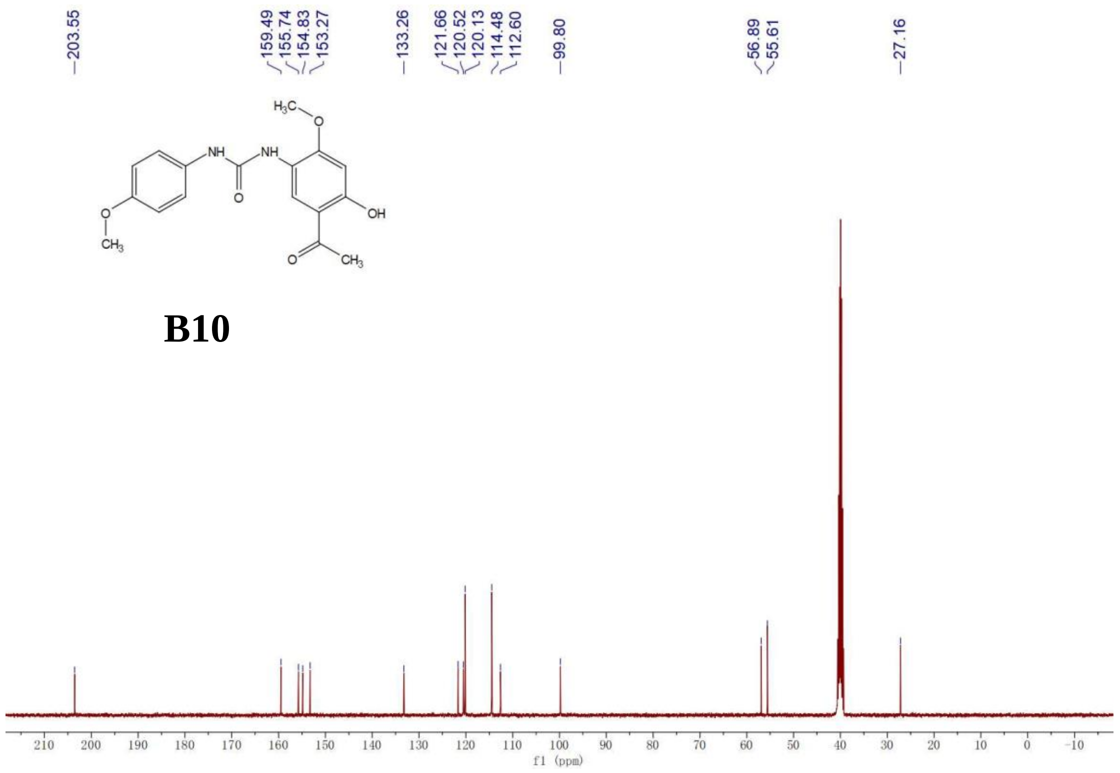

HRMS of compound **B10**

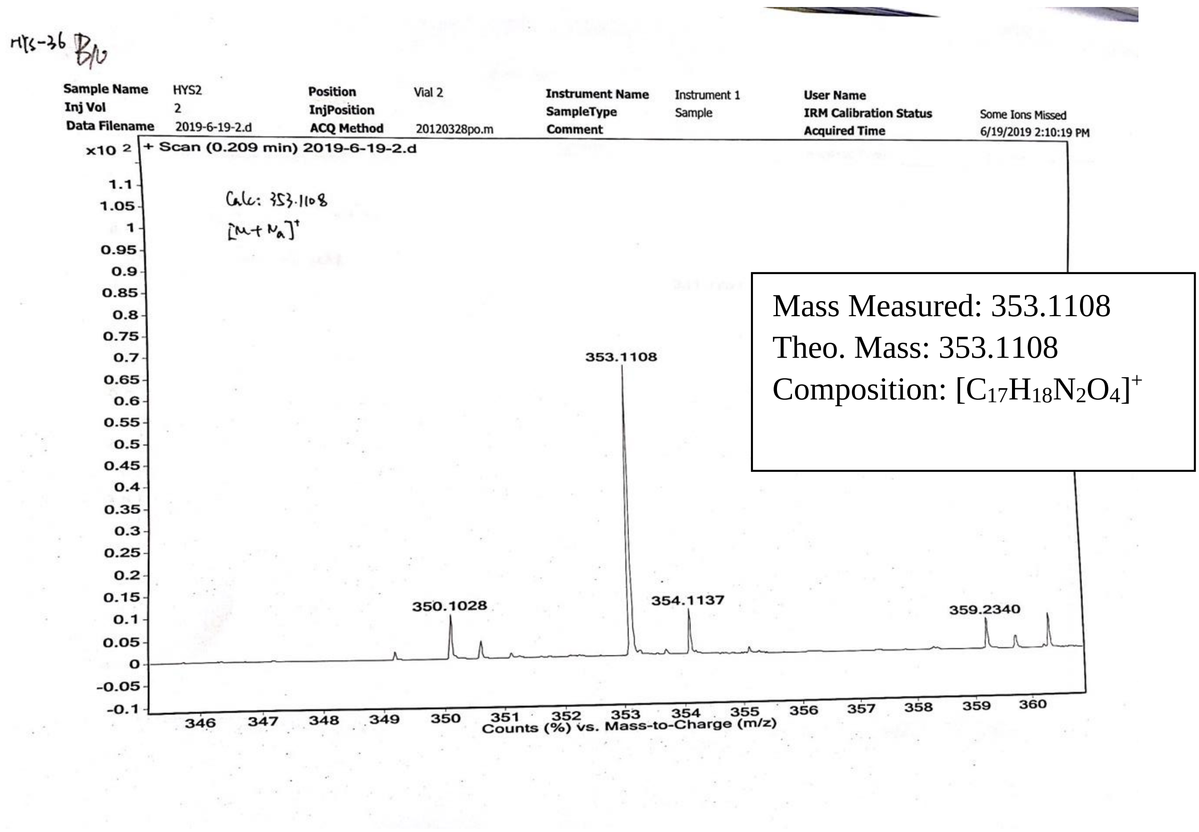

^{1}H,^{13}C NMR of compound **B11**

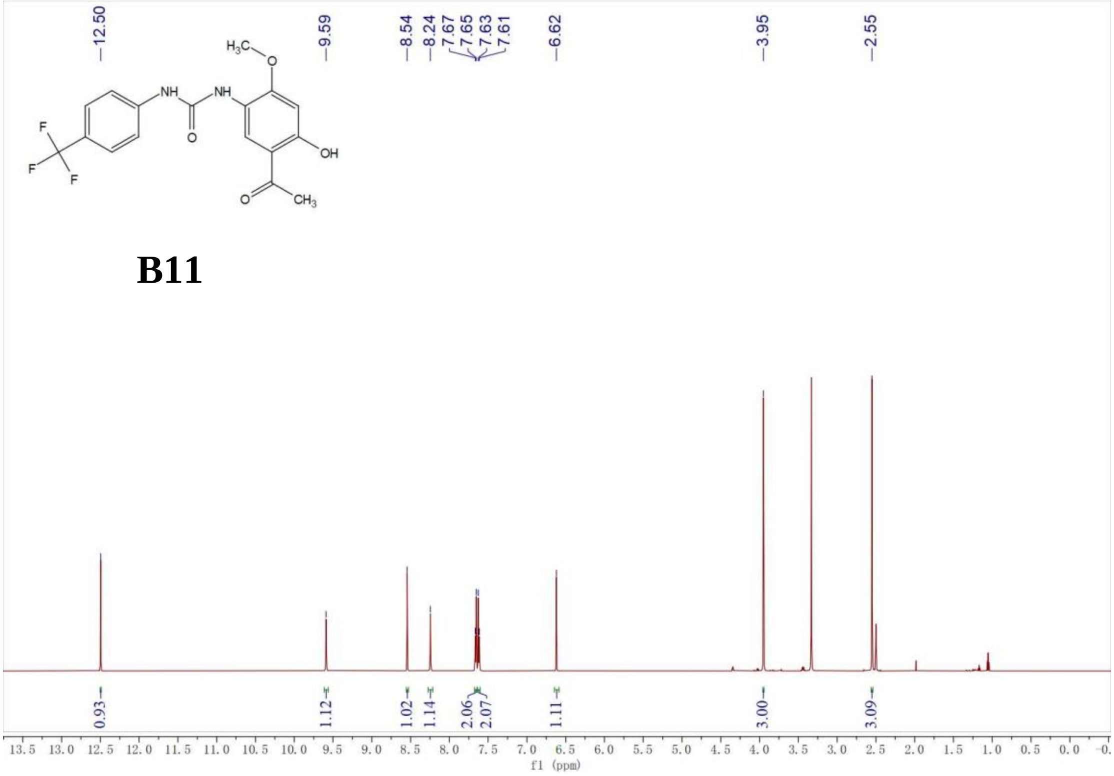

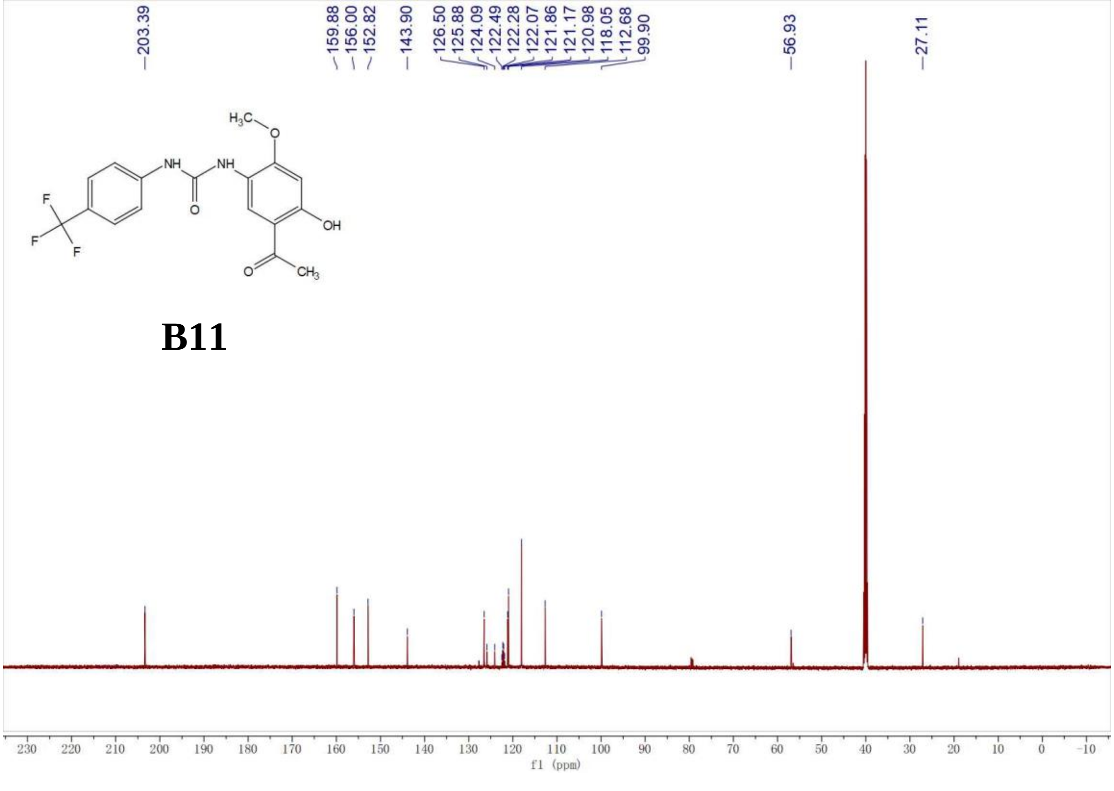

HRMS of compound **B11**

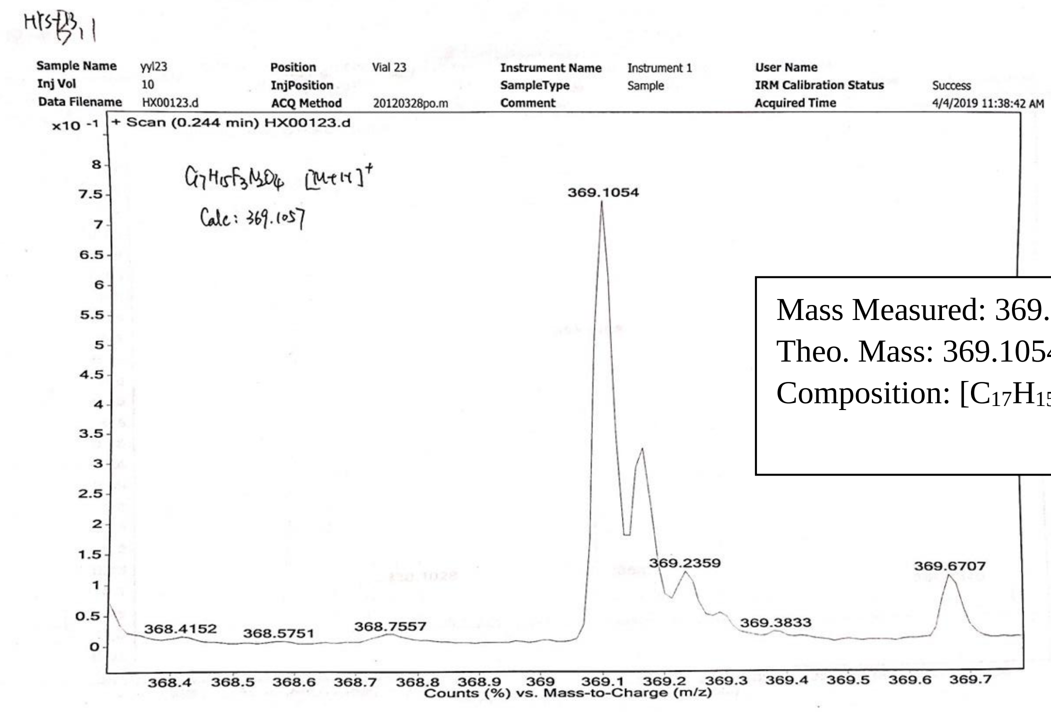

Mass Measured: 369.1057
Theo. Mass: 369.1054.
Composition: $[C_{17}H_{15}F_3N_2O_4]^+$

^{1}H,^{13}C NMR of compound **B12**

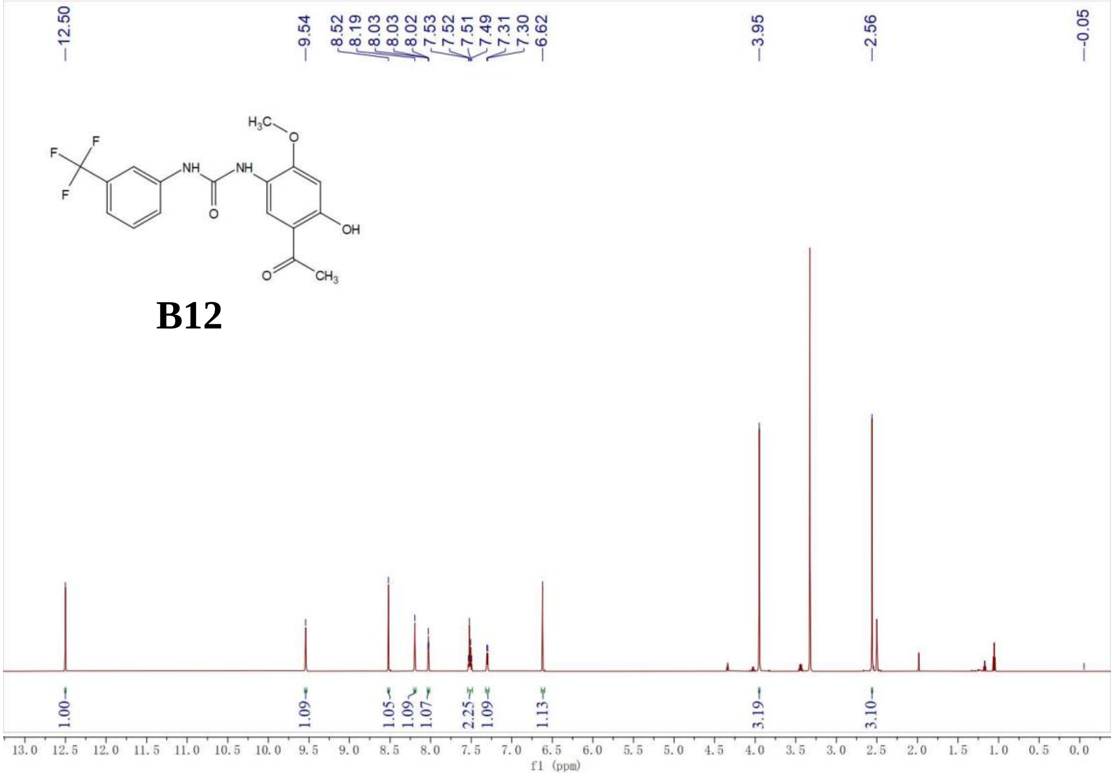

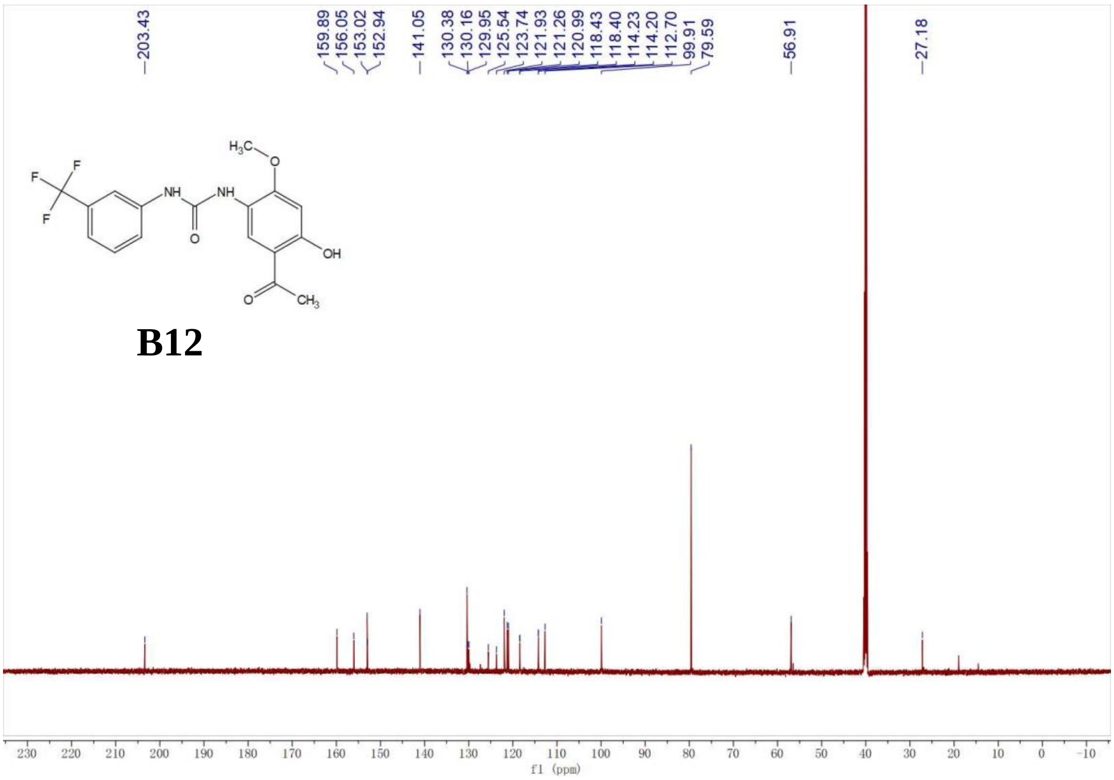

B12

HRMS of compound **B12**

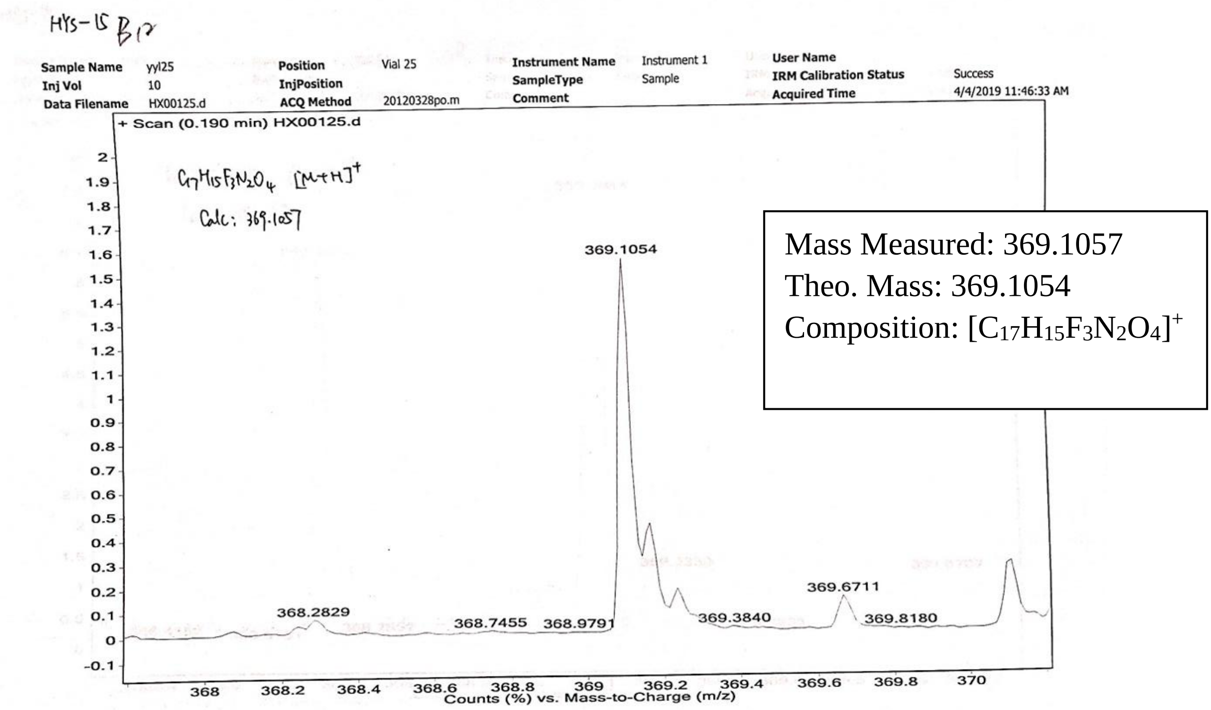

Mass Measured: 369.1057
Theo. Mass: 369.1054
Composition: $[C_{17}H_{15}F_3N_2O_4]^+$

^{1}H,^{13}C NMR of compound **B13**

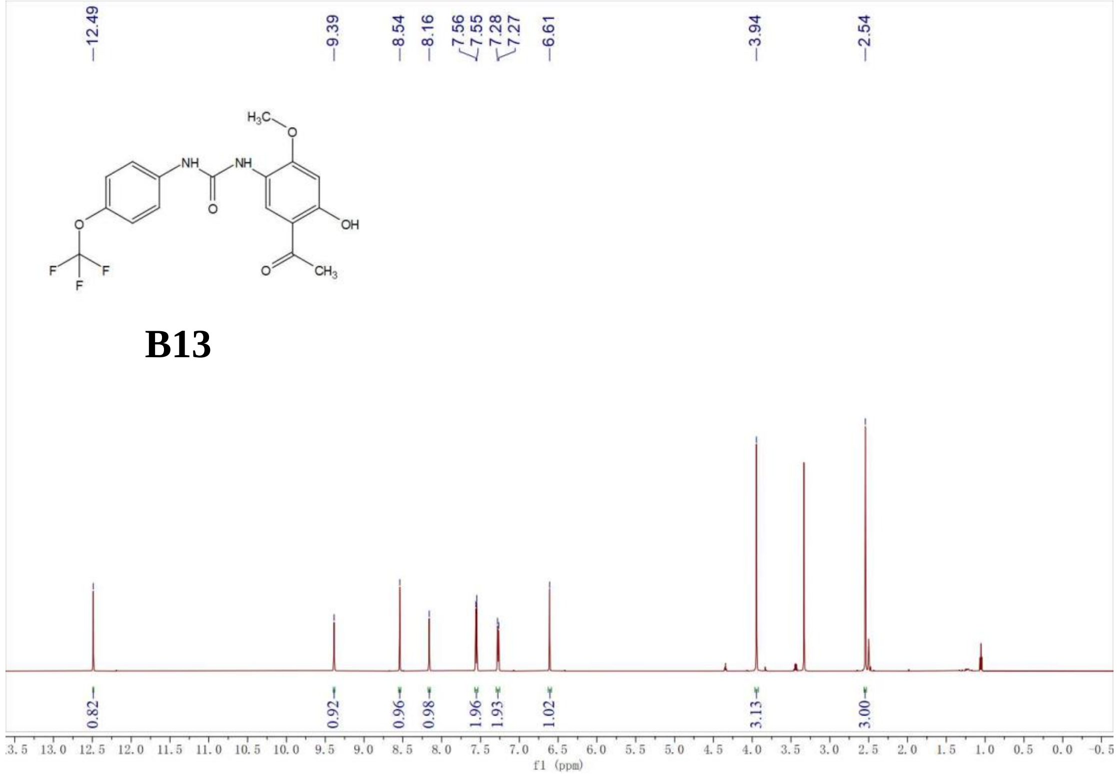

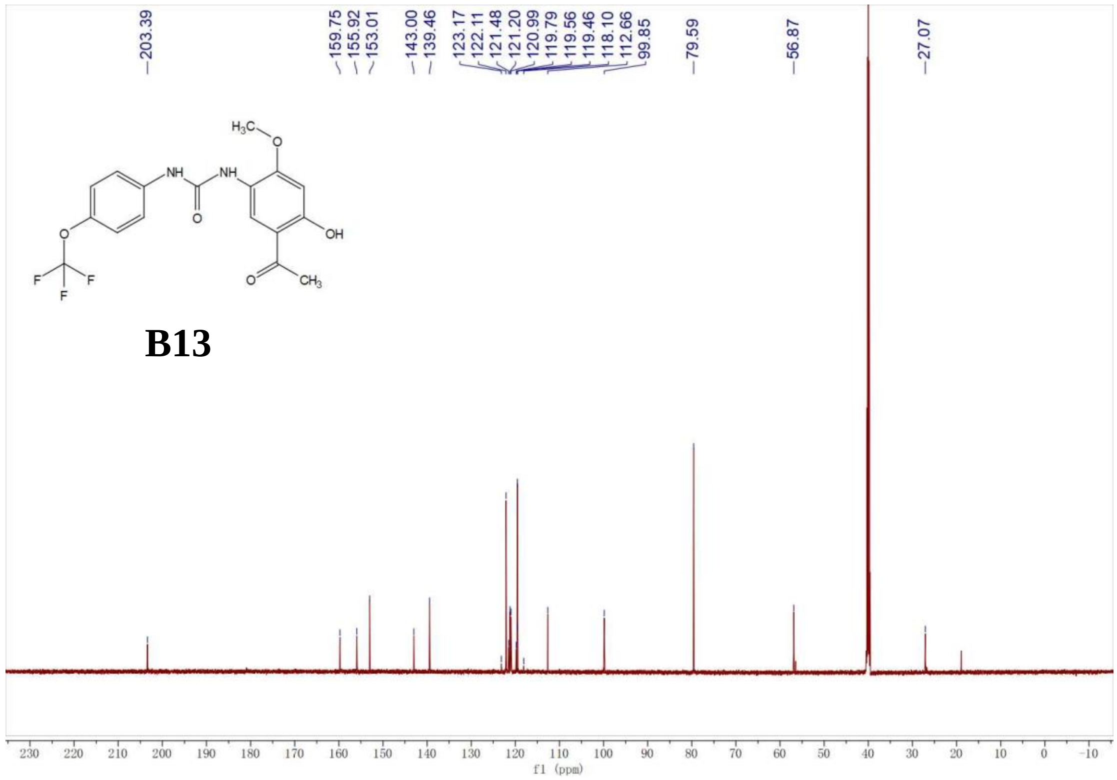

HRMS of compound **B13**

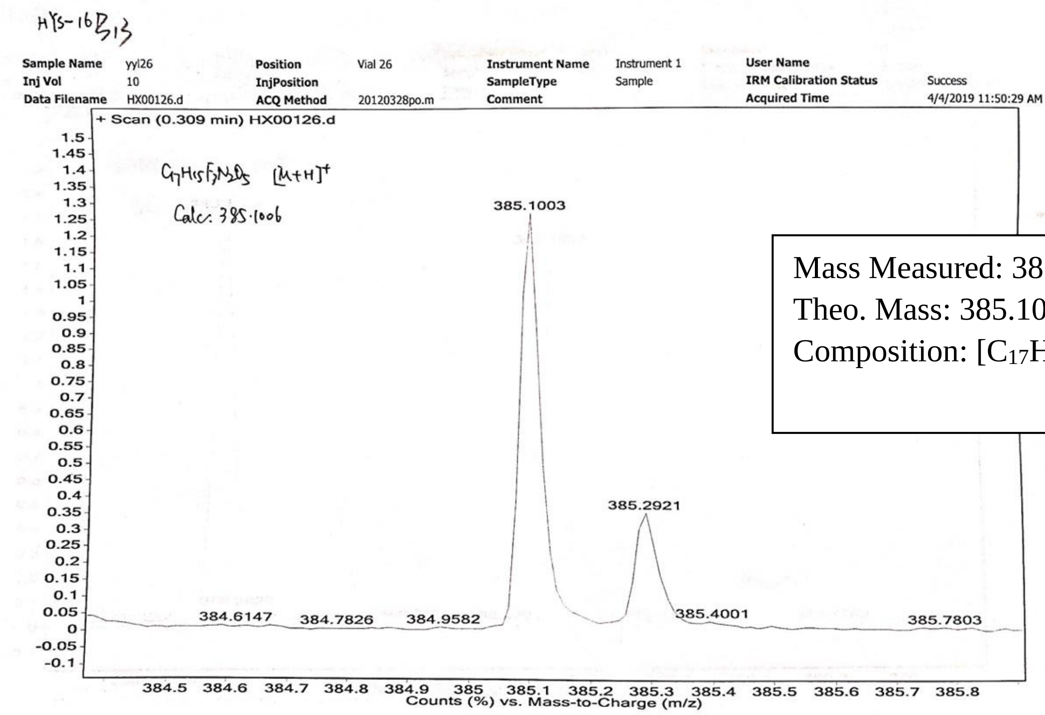

Mass Measured: 385.1006
Theo. Mass: 385.1003
Composition: $[C_{17}H_{15}F_{3}N_{2}O_{5}]^{+}$

^{1}H,^{13}C NMR of compound **C1**

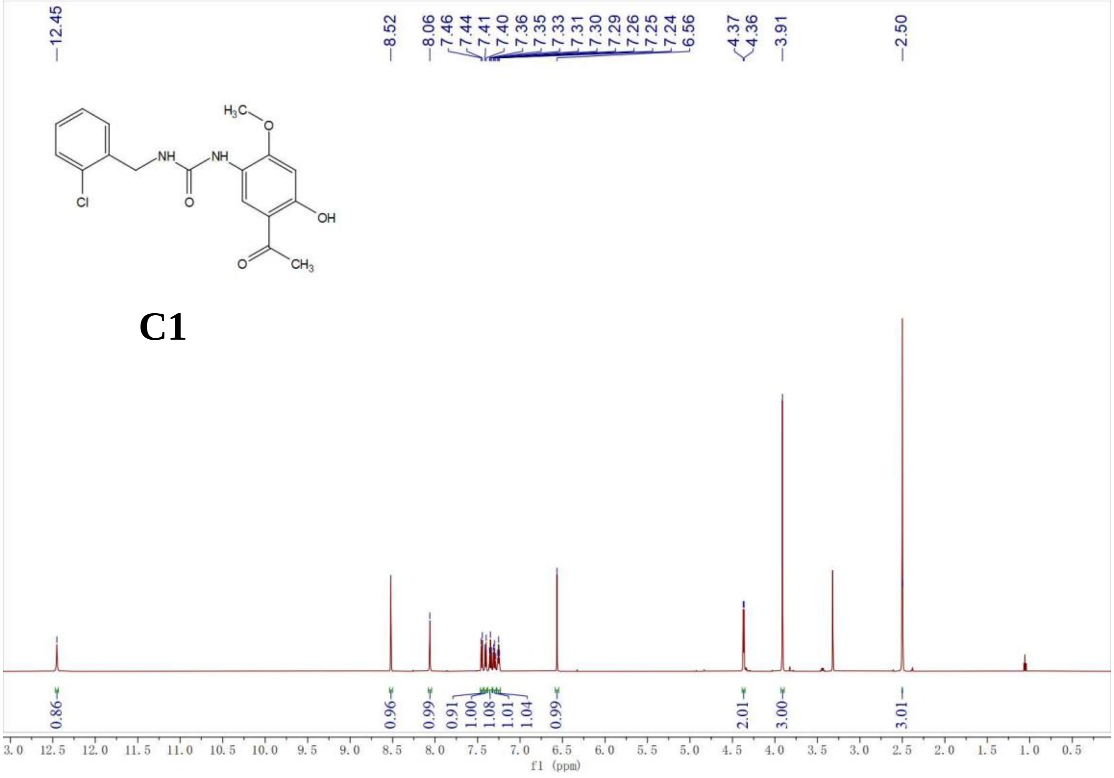

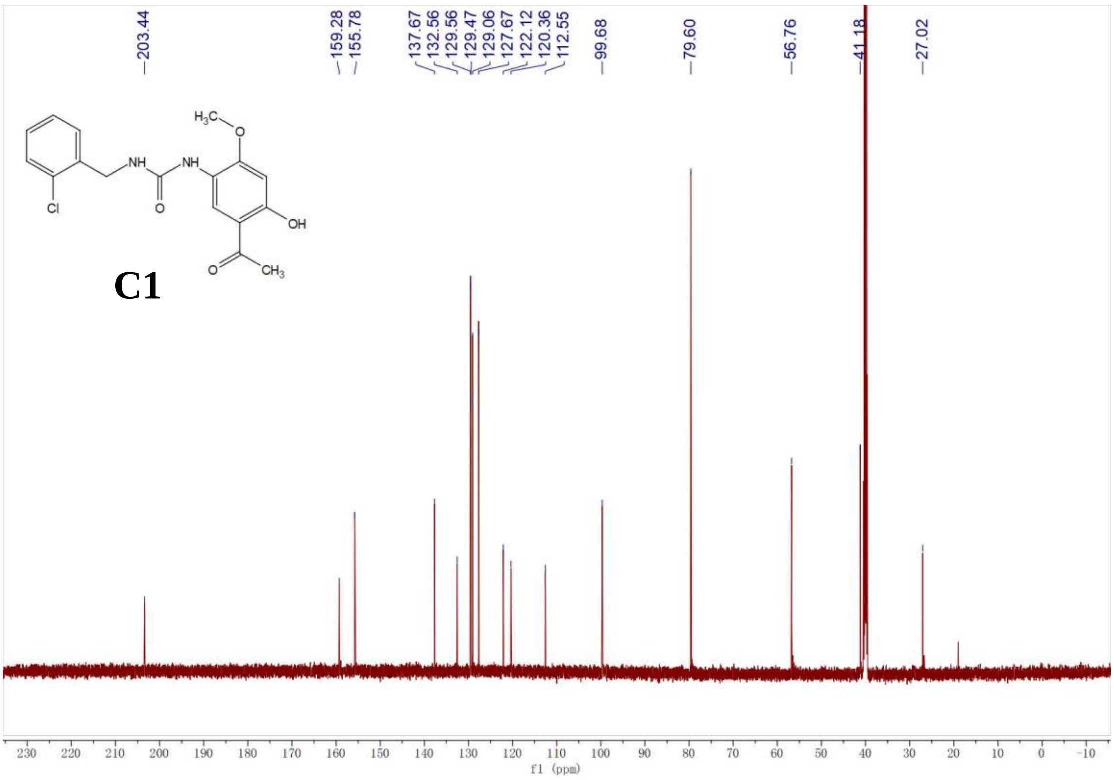

HRMS of compound **C1**

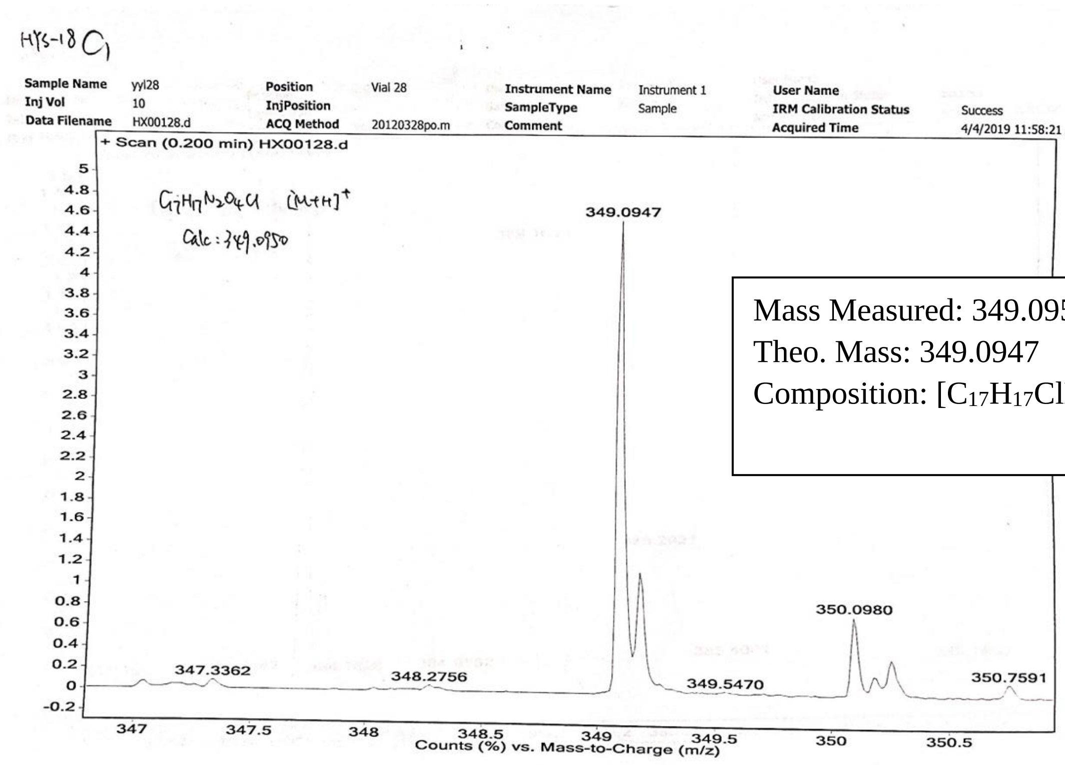

Mass Measured: 349.0950
Theo. Mass: 349.0947
Composition: $[C_{17}H_{17}ClN_2O_4]^+$

^1H,^{13}C NMR of compound **C2**

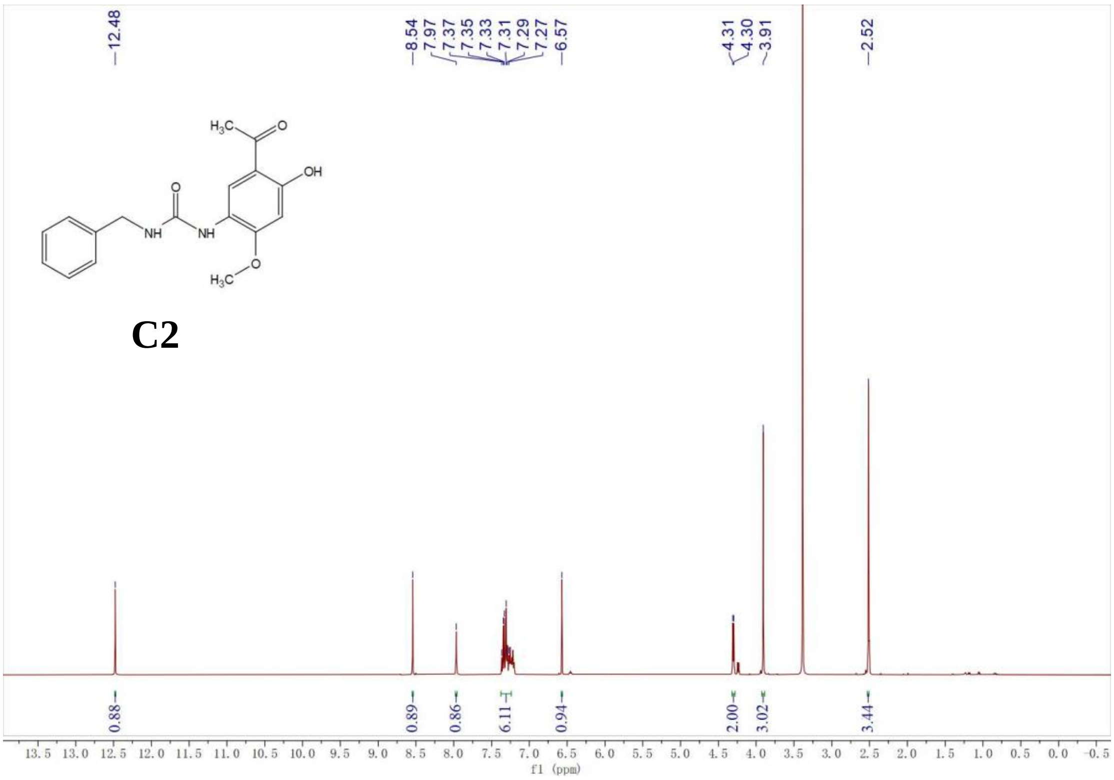

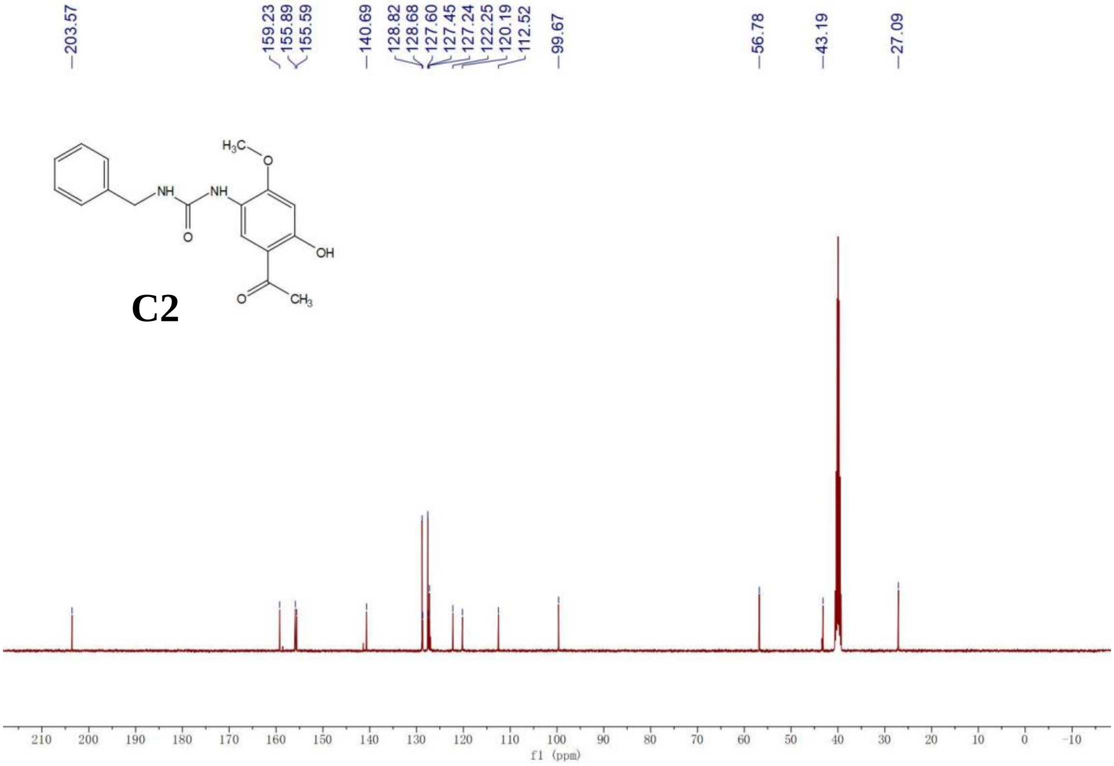

HRMS of compound **C2**

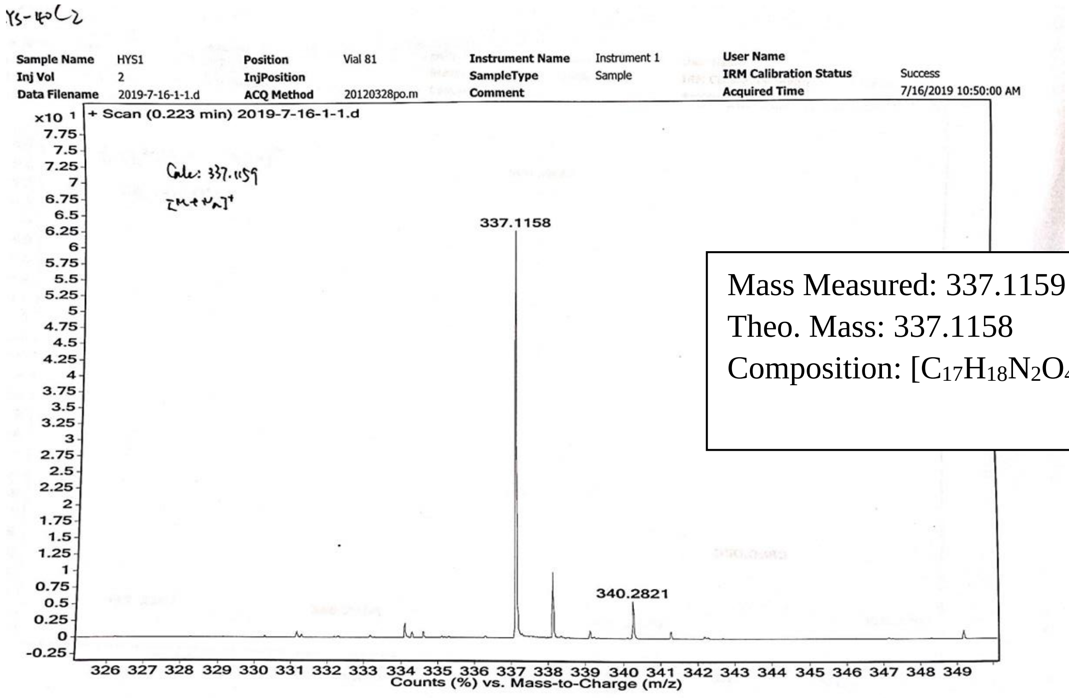

Mass Measured: 337.1159
Theo. Mass: 337.1158
Composition: $[C_{17}H_{18}N_2O_4]^+$

^{1}H,^{13}C NMR of compound **C3**

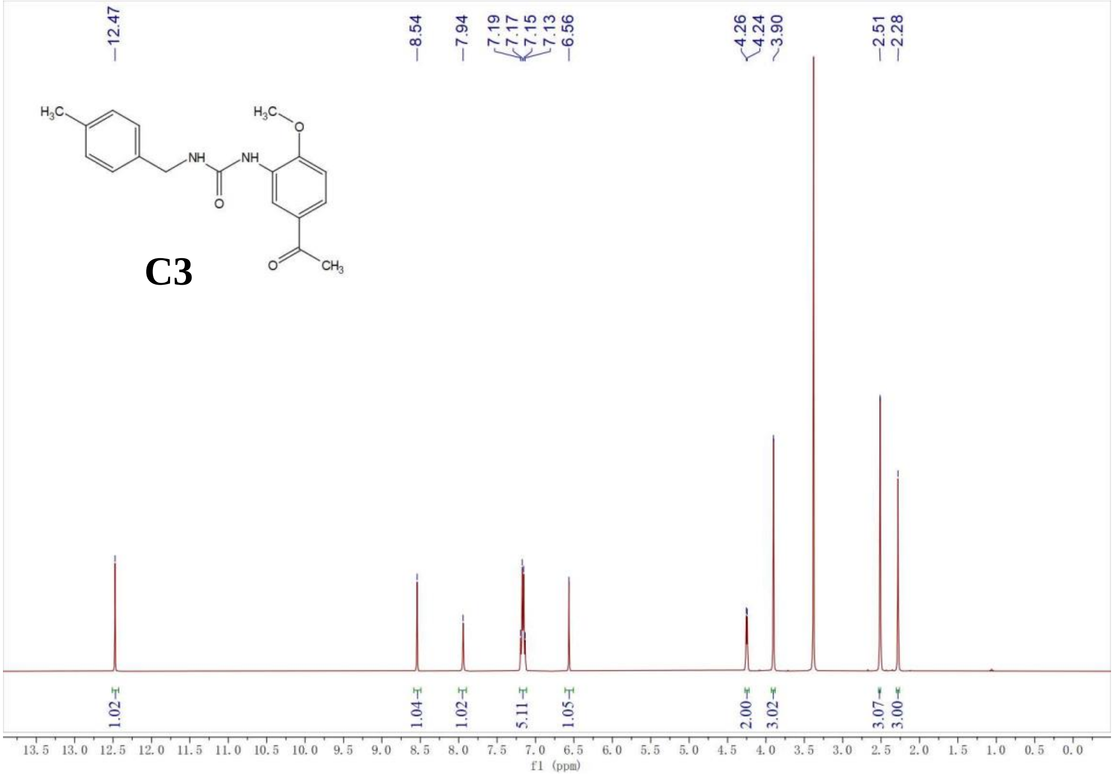

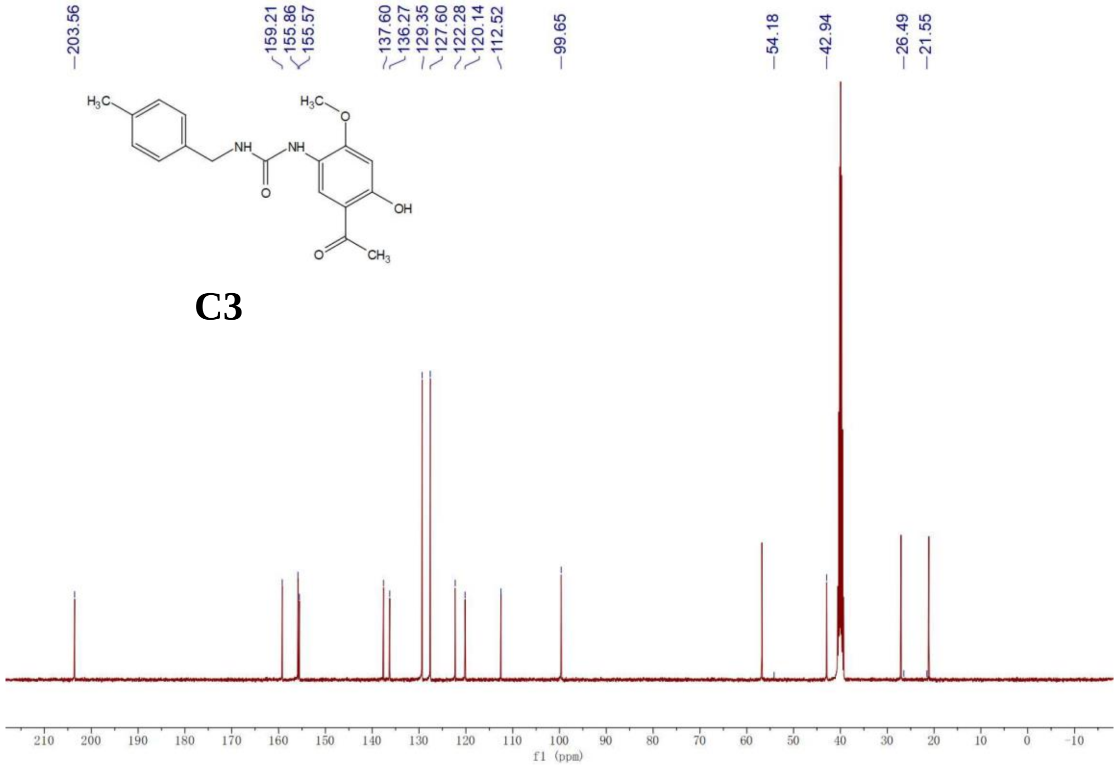

HRMS of compound **C3**

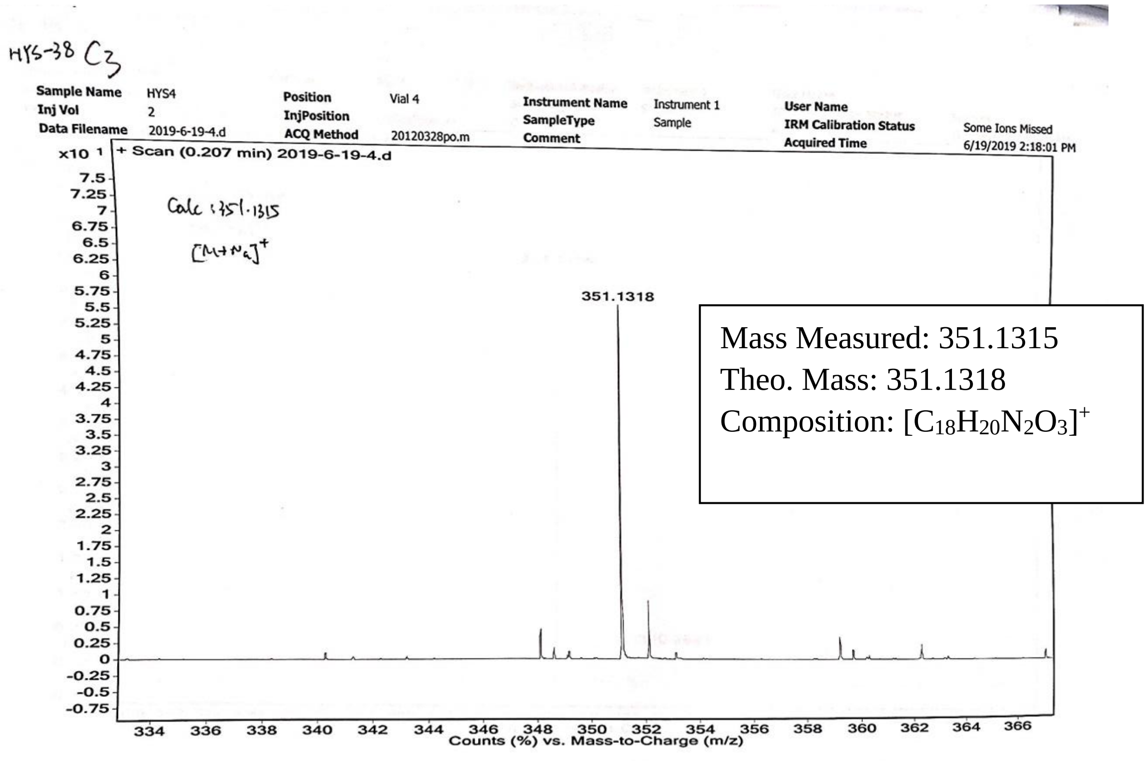

^{1}H,^{13}C NMR of compound **C4**

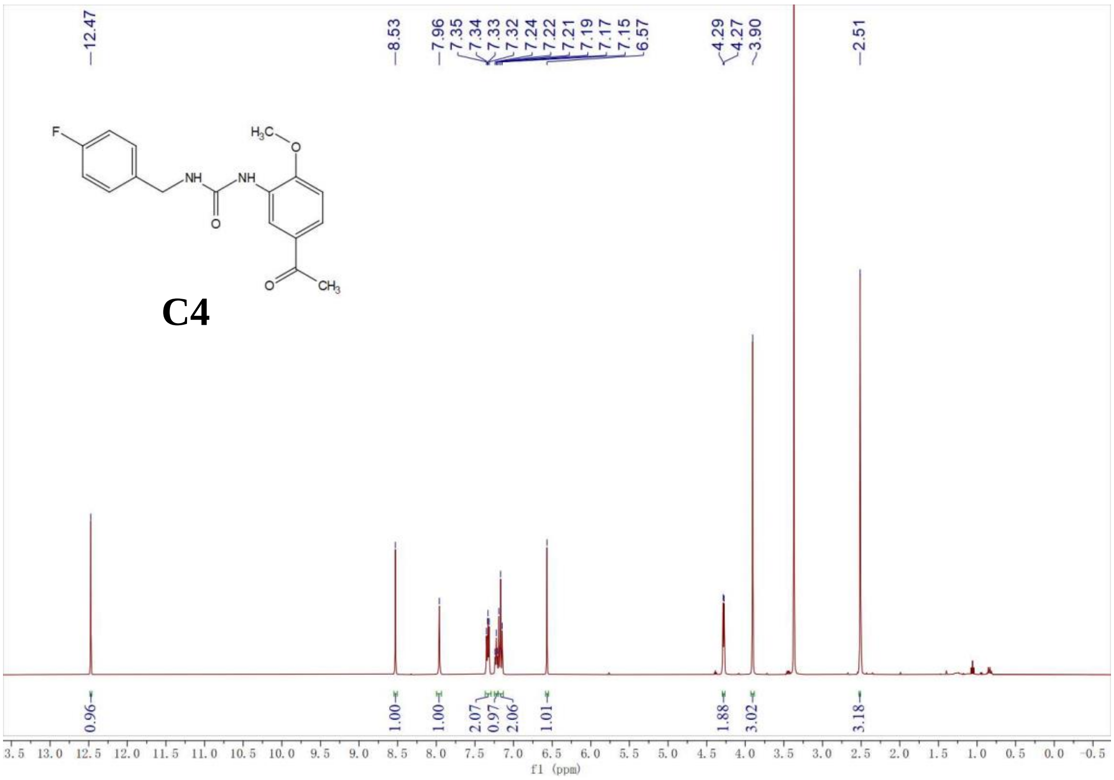

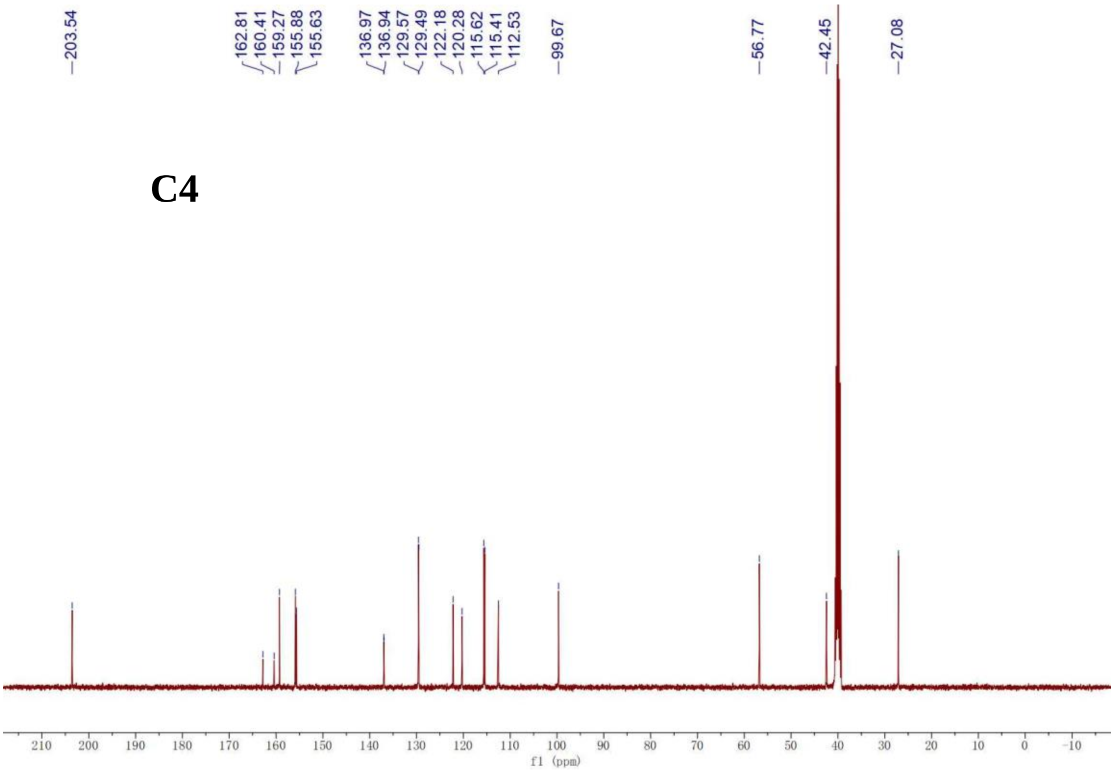

HRMS of compound **C4**

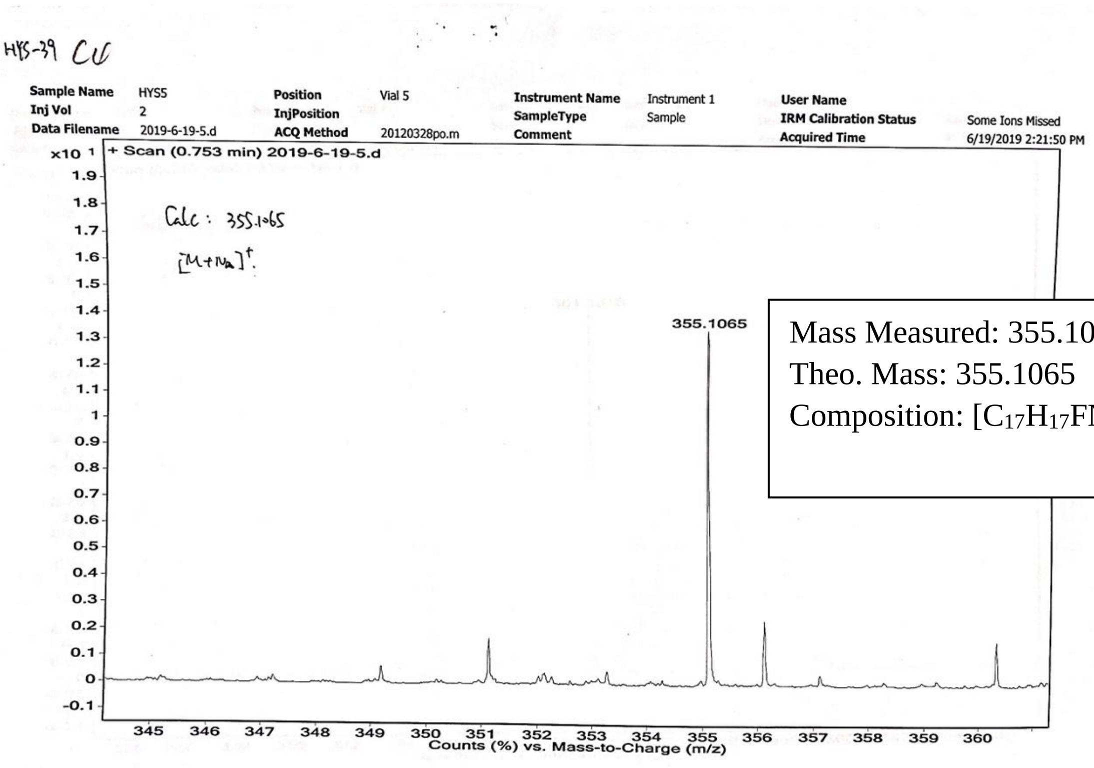

Mass Measured: 355.1065
Theo. Mass: 355.1065
Composition: $[C_{17}H_{17}FN_2O_3]^+$

^1H,^{13}C NMR of compound **C5**

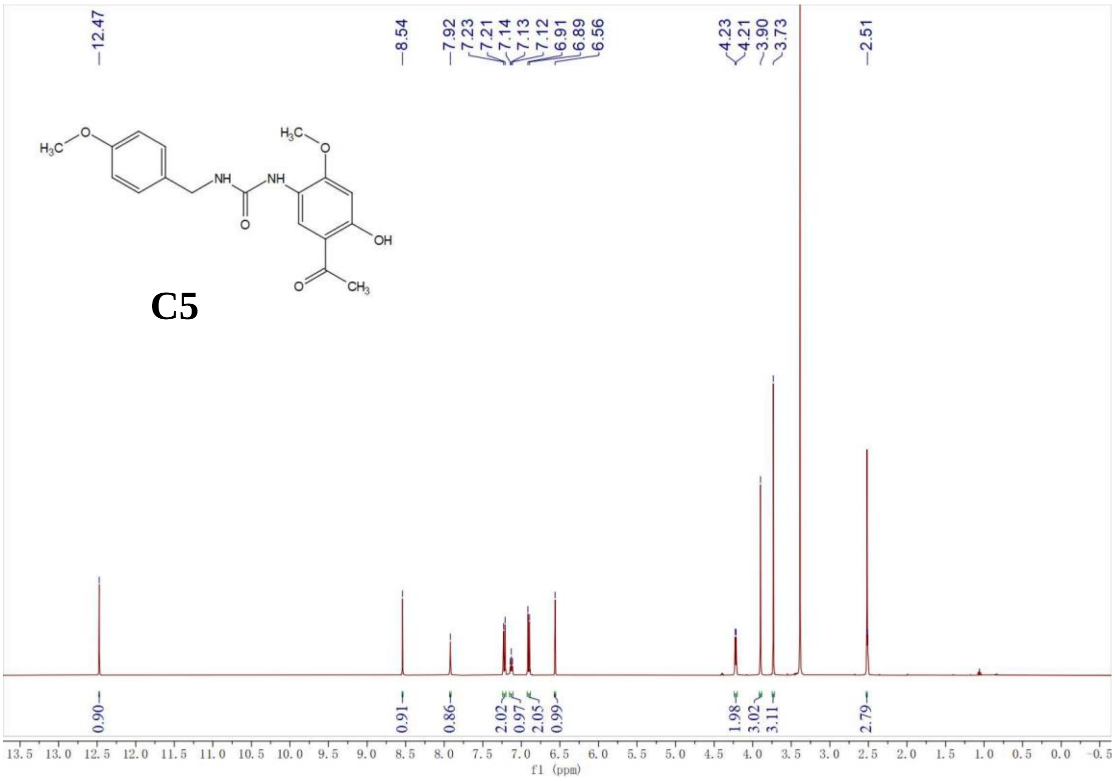

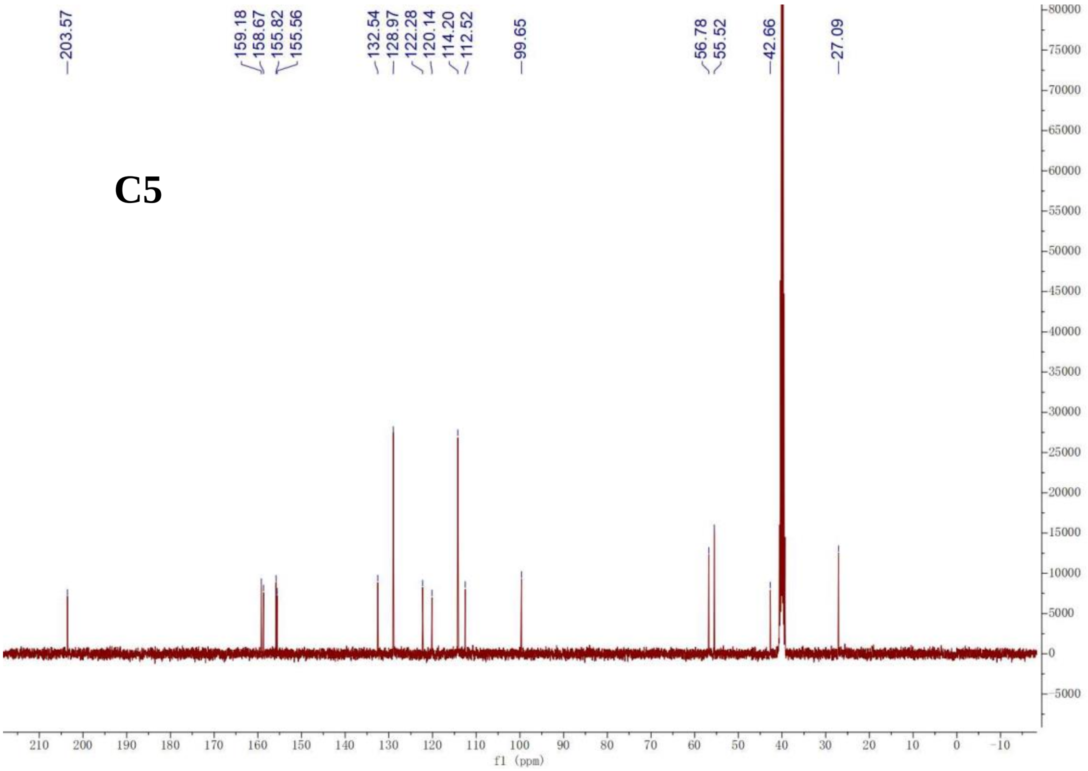

HRMS of compound **C5**

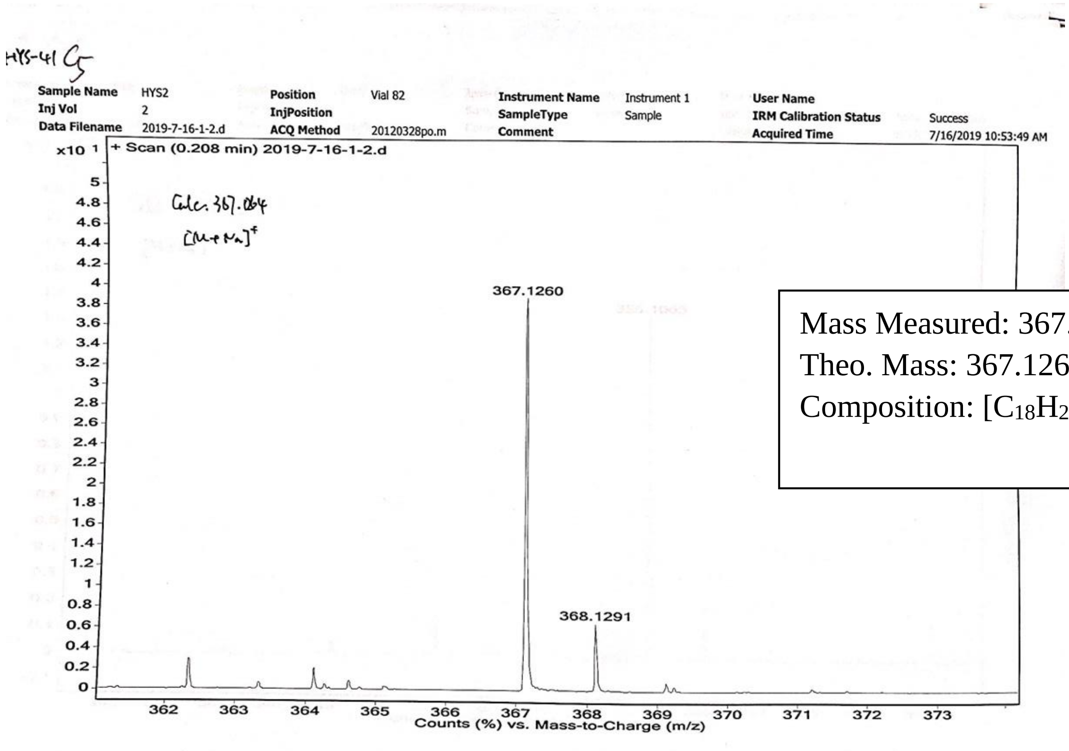

Mass Measured: 367.1264
Theo. Mass: 367.1260
Composition: $[C_{18}H_{20}N_2O_5]^+$

^1H,^{13}C NMR of compound **C6**

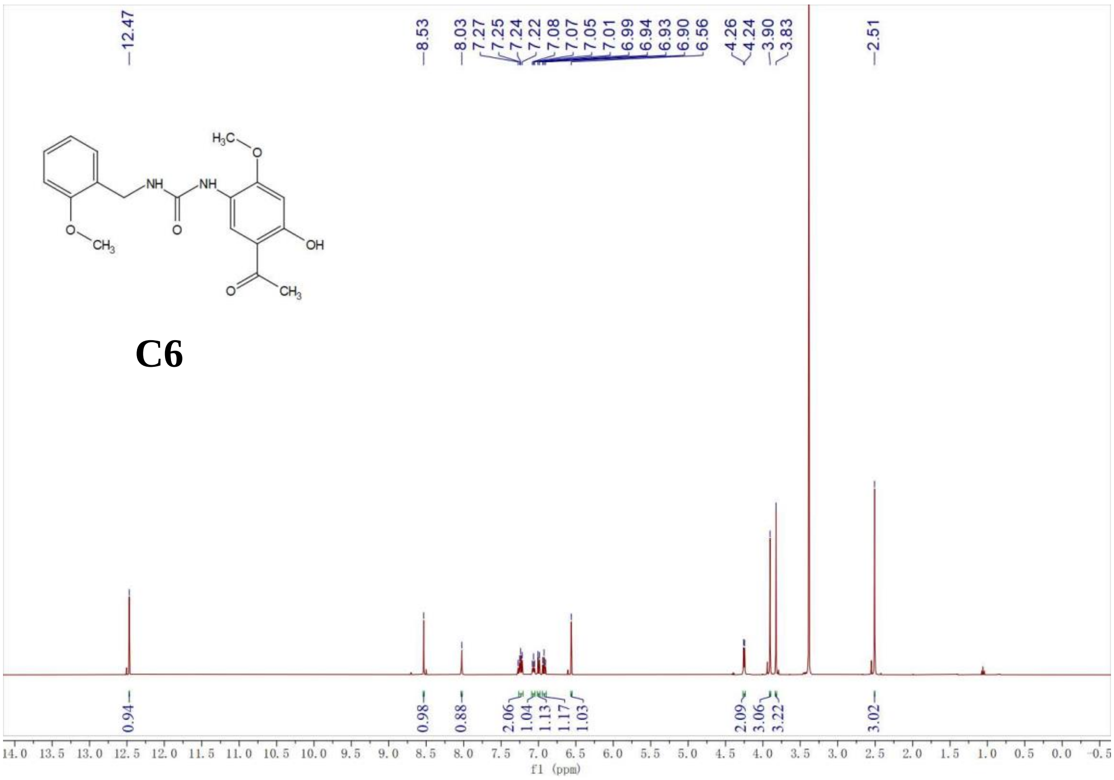

C6

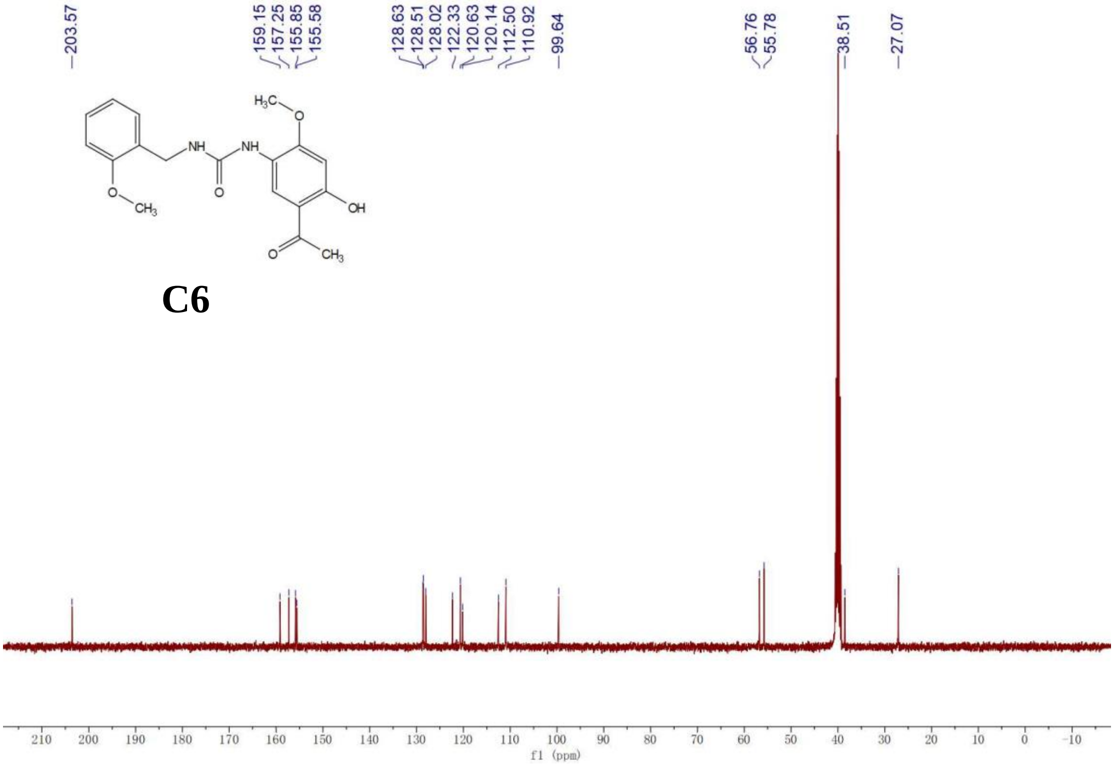

C6

HRMS of compound **C6**

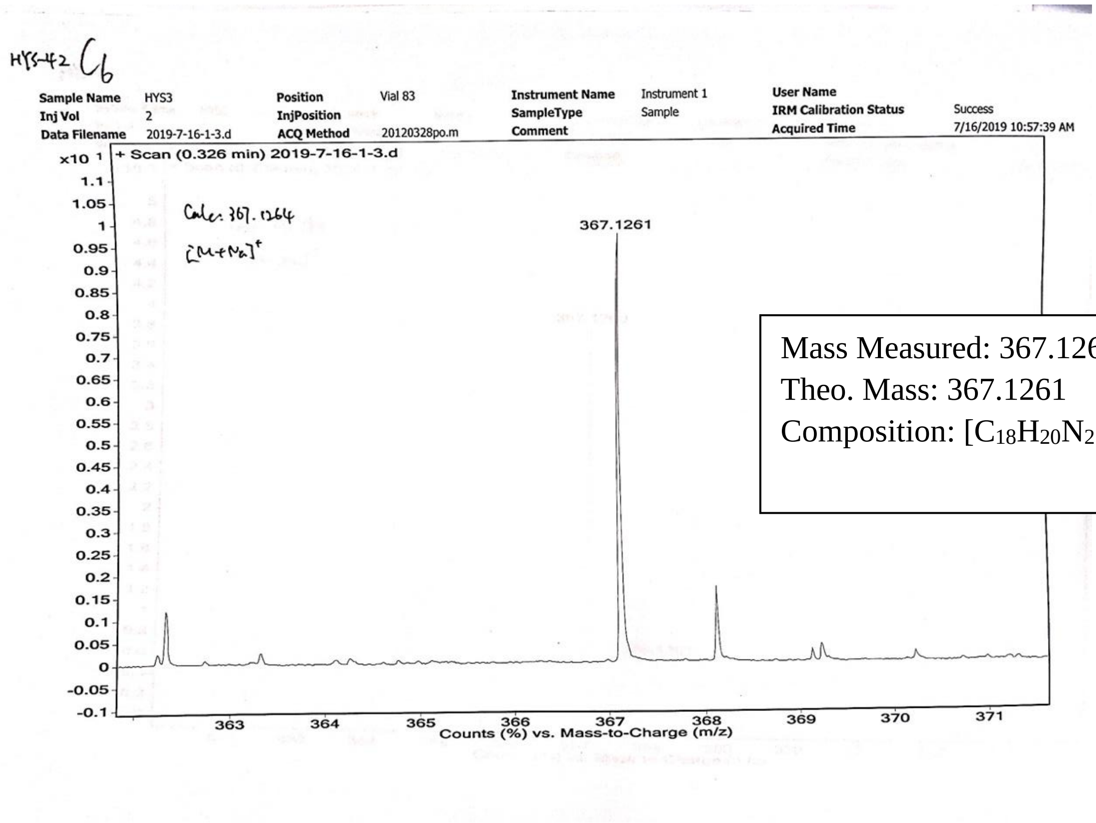

Mass Measured: 367.1264
Theo. Mass: 367.1261
Composition: $[C_{18}H_{20}N_2O_5]^+$

^1H,^{13}C NMR of compound **C7**

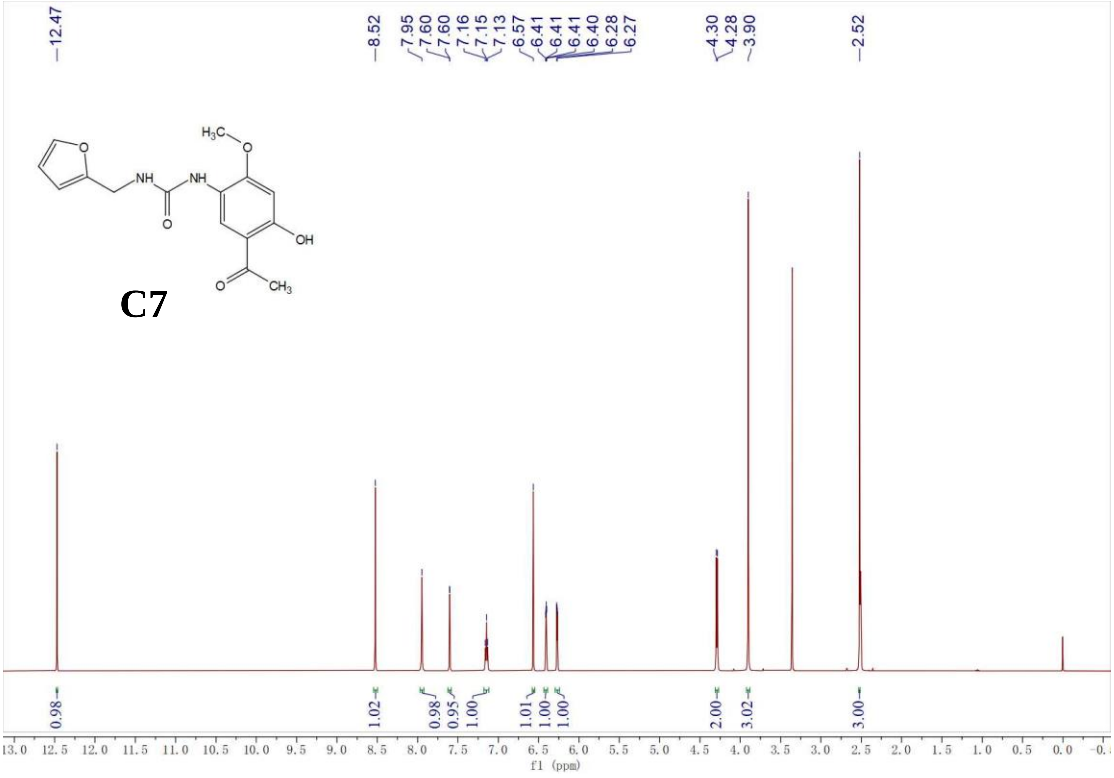

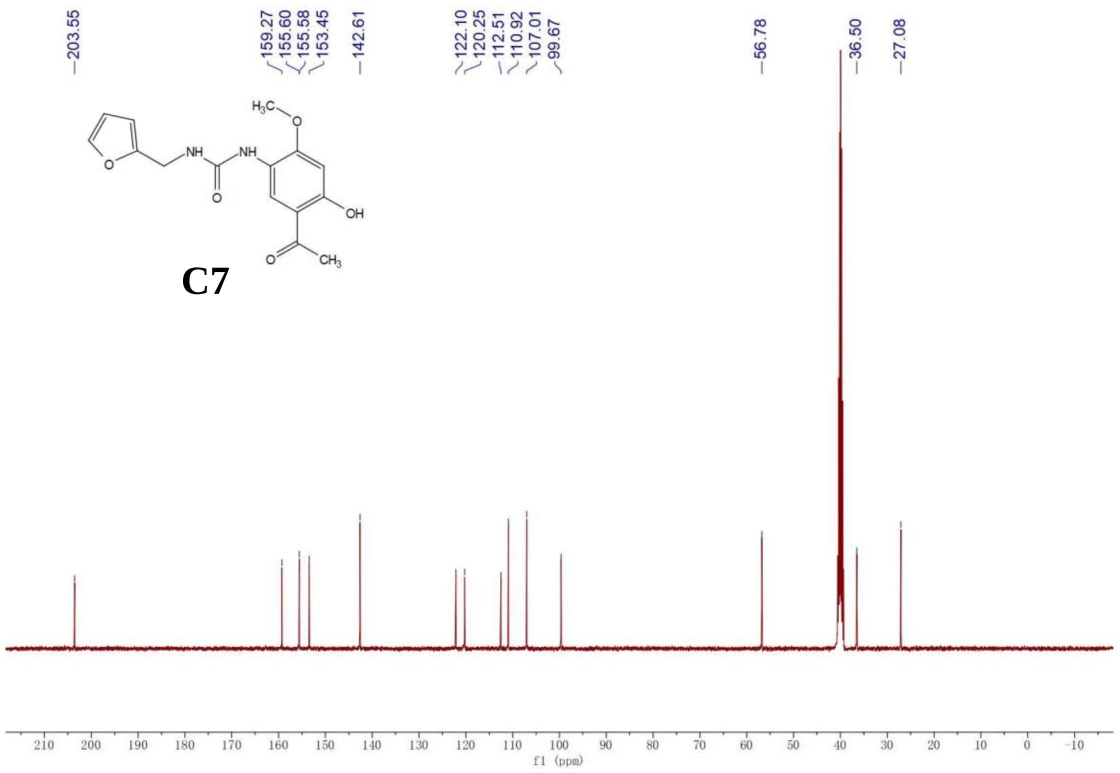

HRMS of compound **C7**

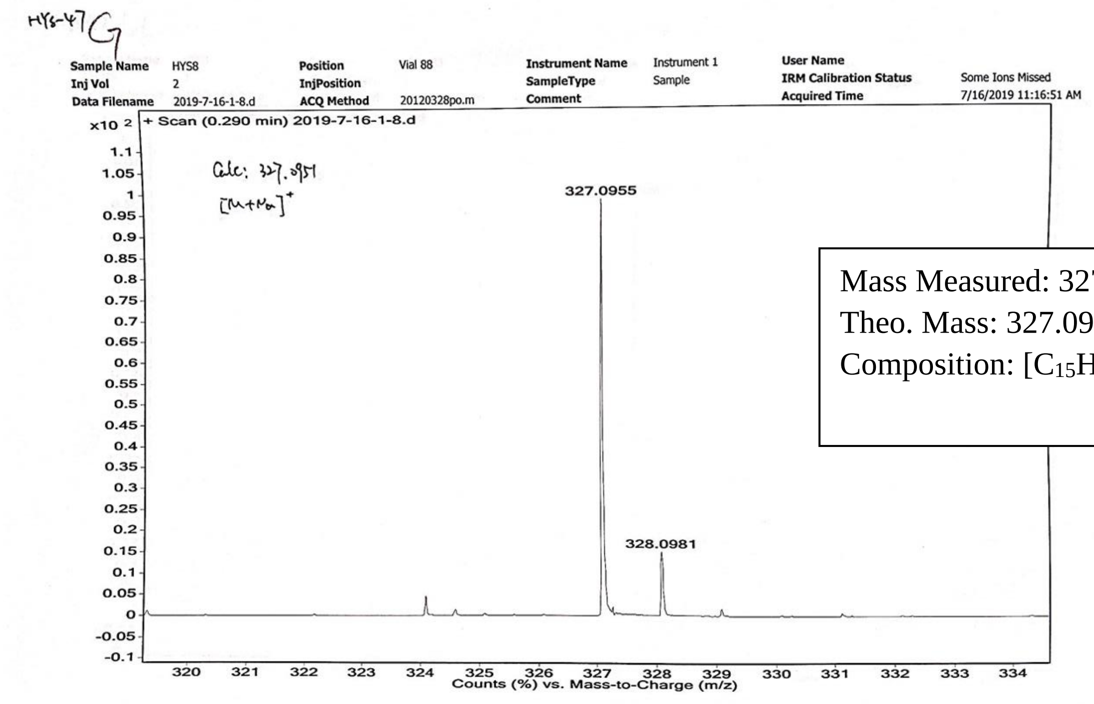

Mass Measured: 327.0951
Theo. Mass: 327.0955
Composition: $[C_{15}H_{16}N_2O_5]^+$

^{1}H,^{13}C NMR of compound **D1**

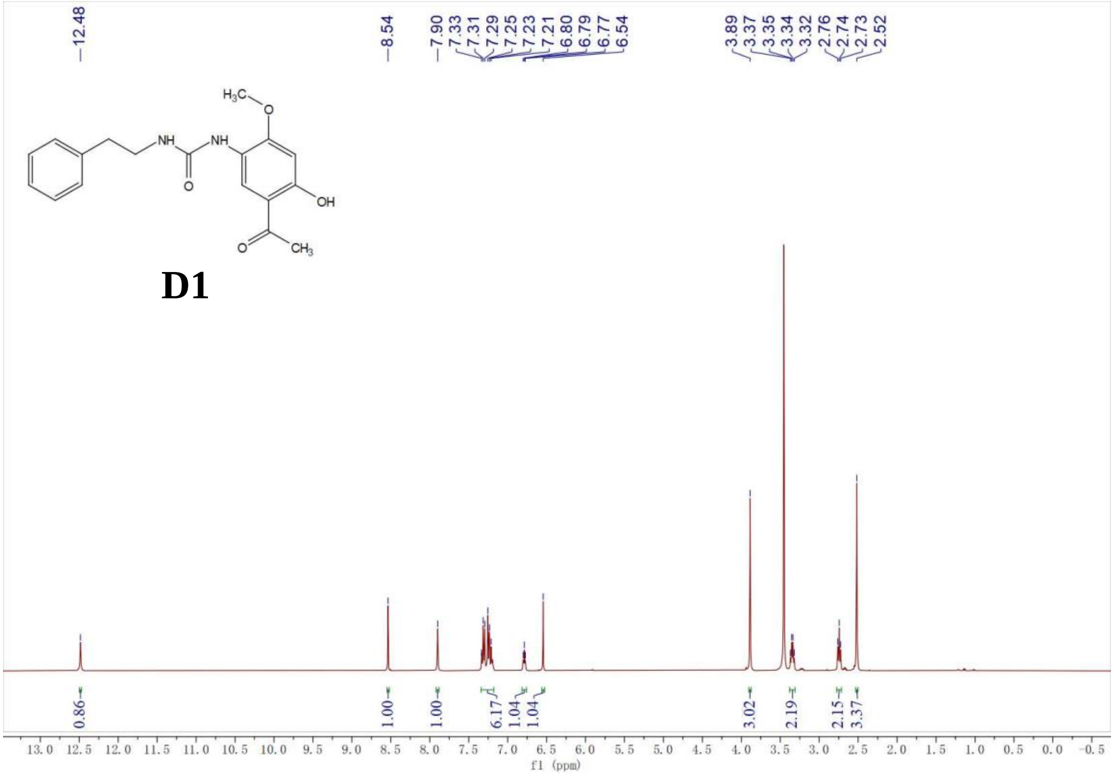

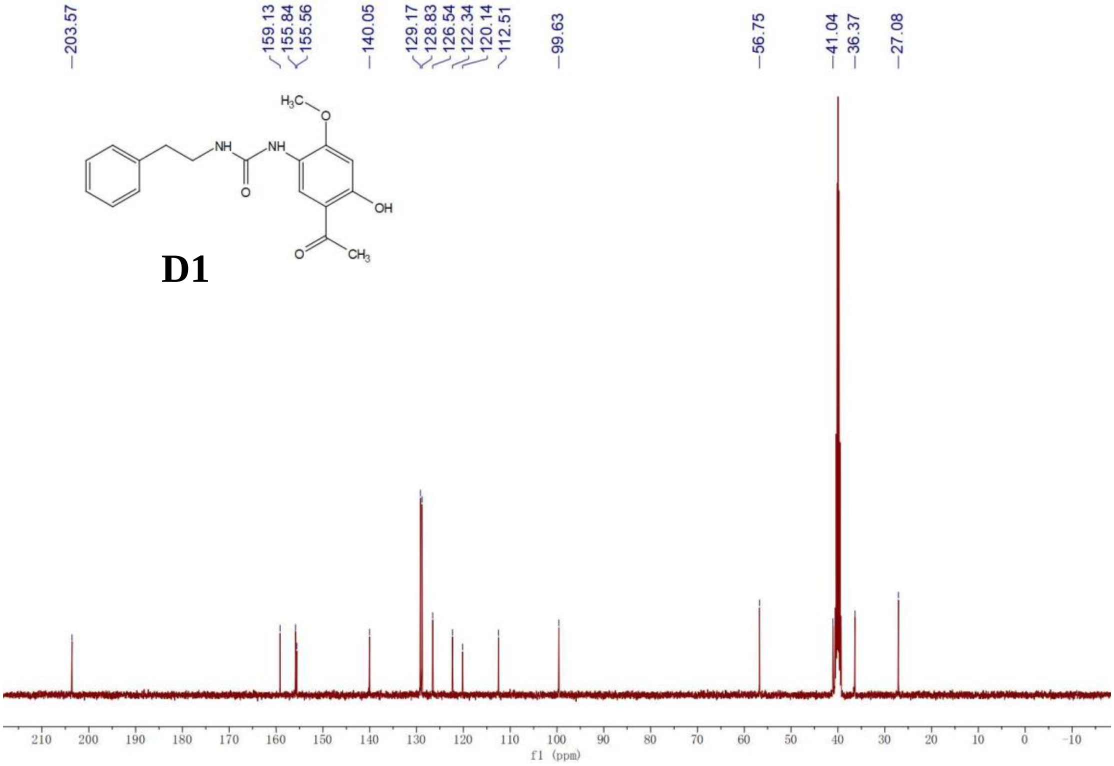

HRMS of compound **D1**

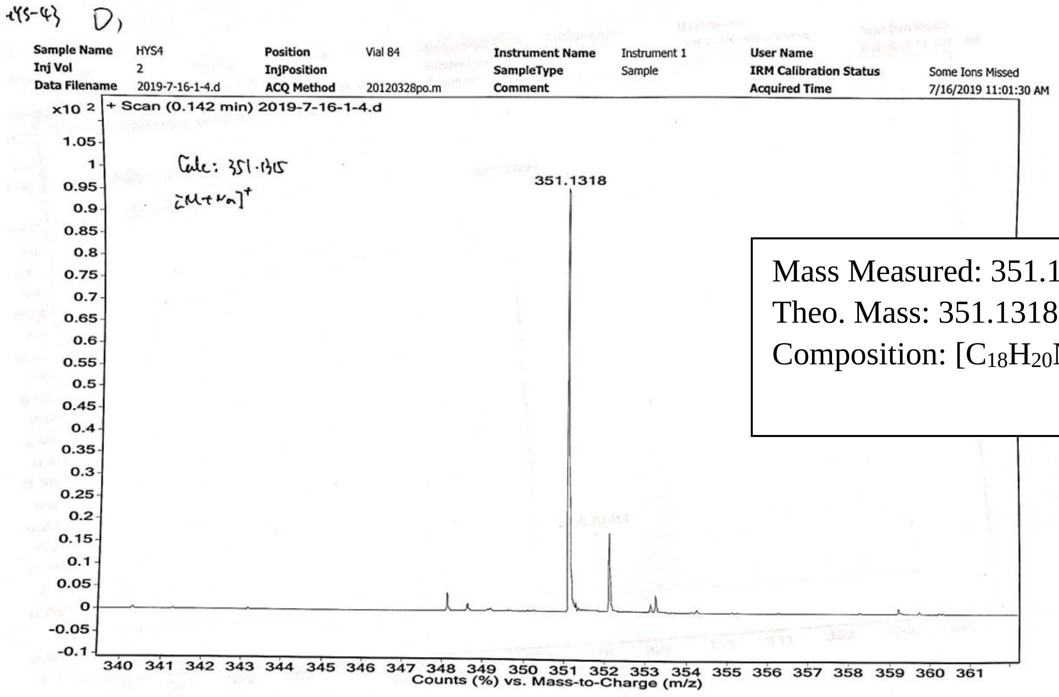

Mass Measured: 351.1315
Theo. Mass: 351.1318
Composition: $[C_{18}H_{20}N_2O_4]^+$

^{1}H,^{13}C NMR of compound **D2**

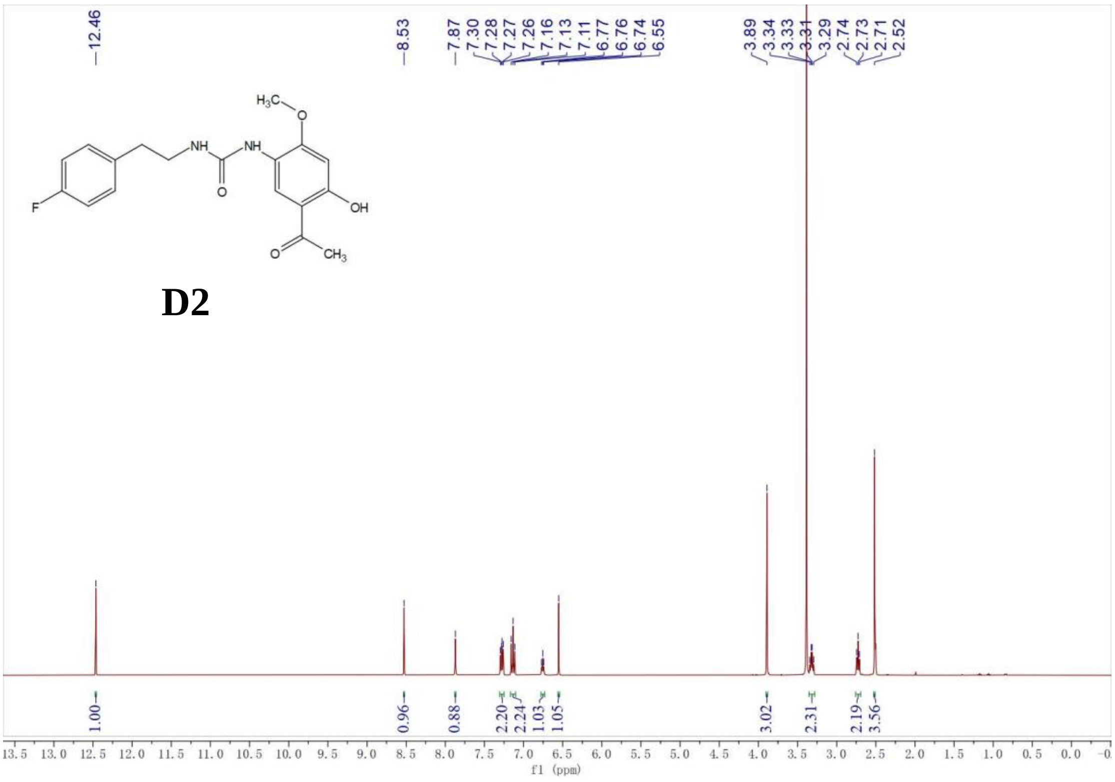

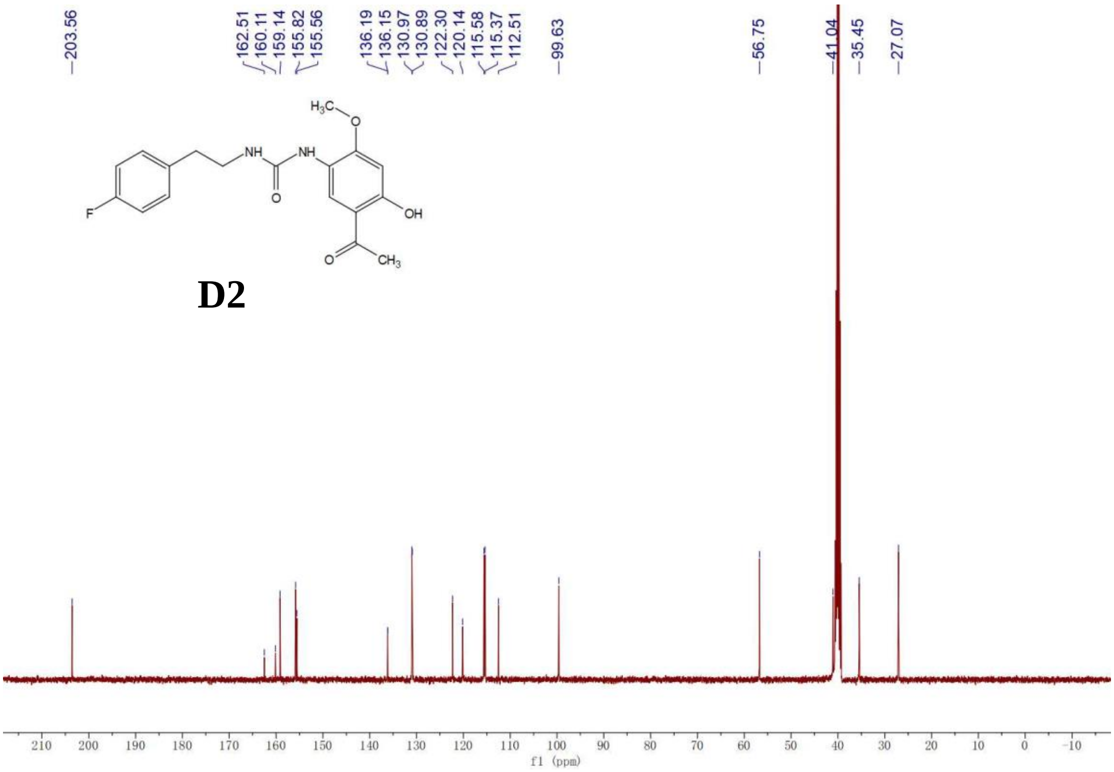

HRMS of compound **D2**

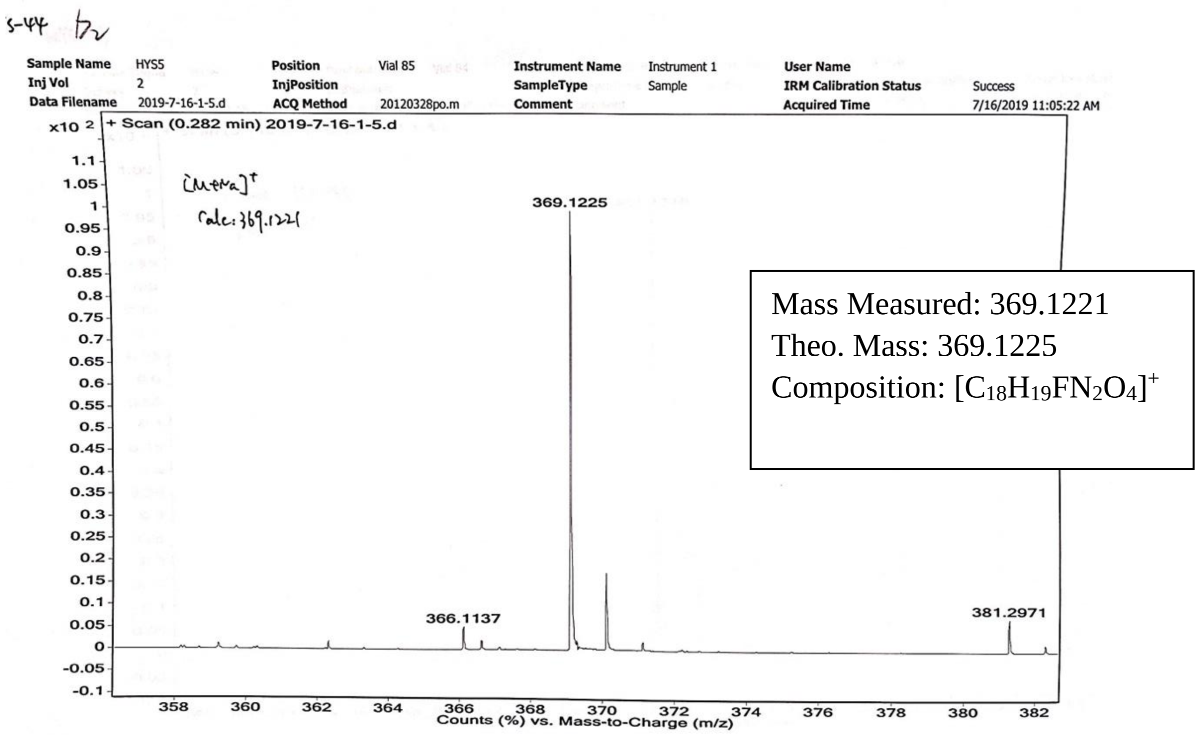

^{1}H,^{13}C NMR of compound **D3**

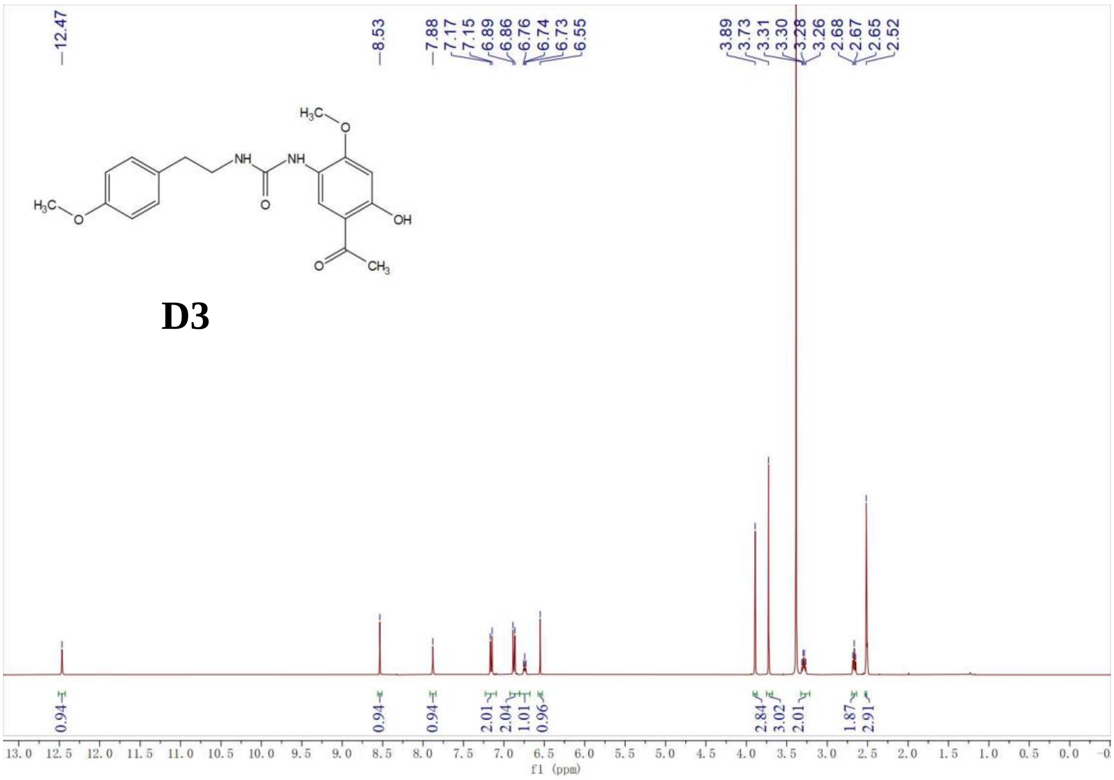

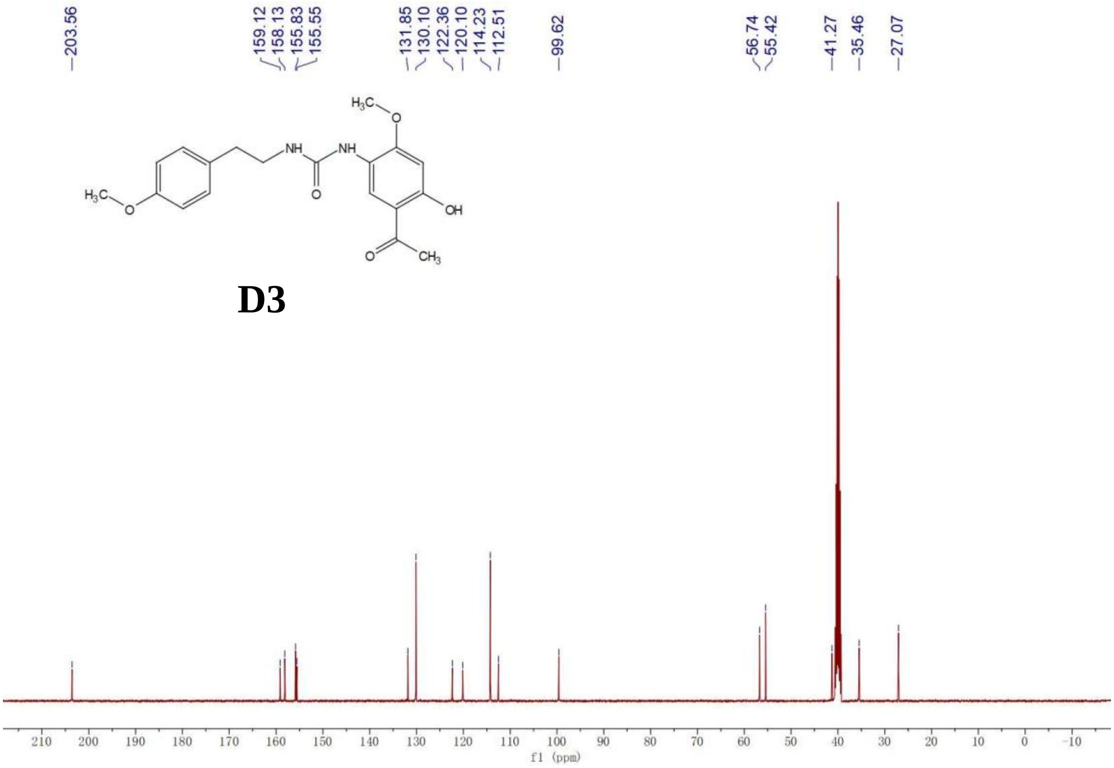

HRMS of compound **D3**

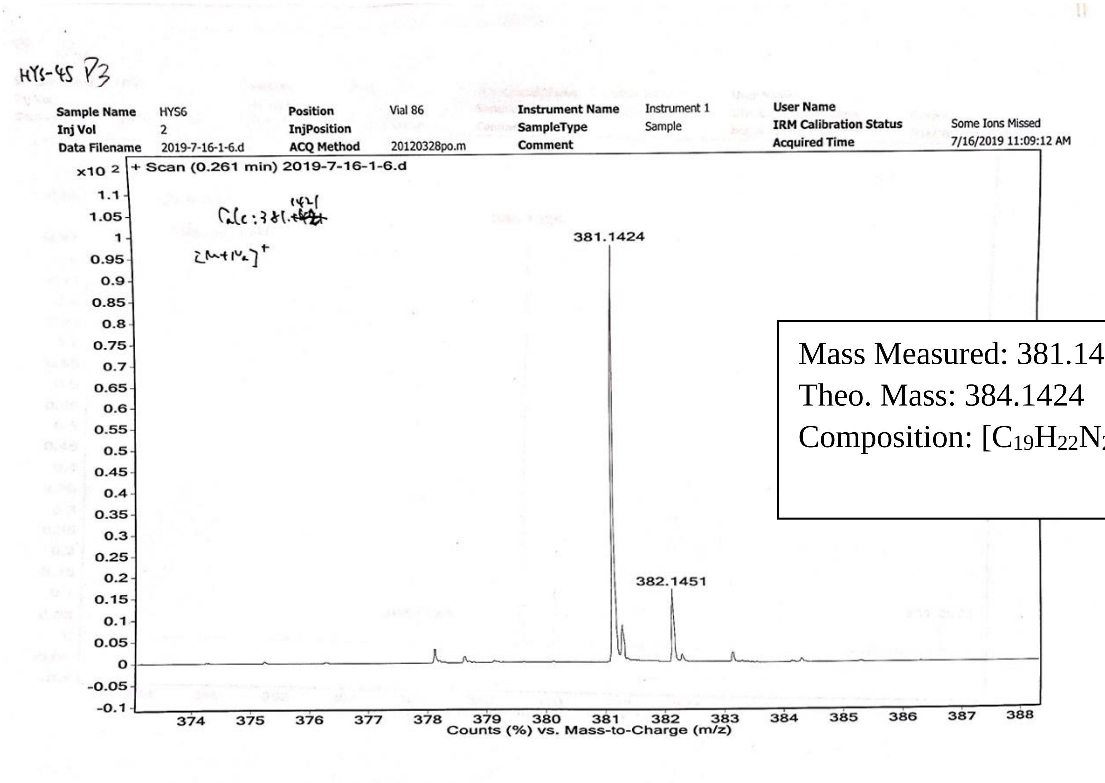

Mass Measured: 381.1421
Theo. Mass: 384.1424
Composition: $[C_{19}H_{22}N_2O_5]^+$

^{1}H,^{13}C NMR of compound **D4**

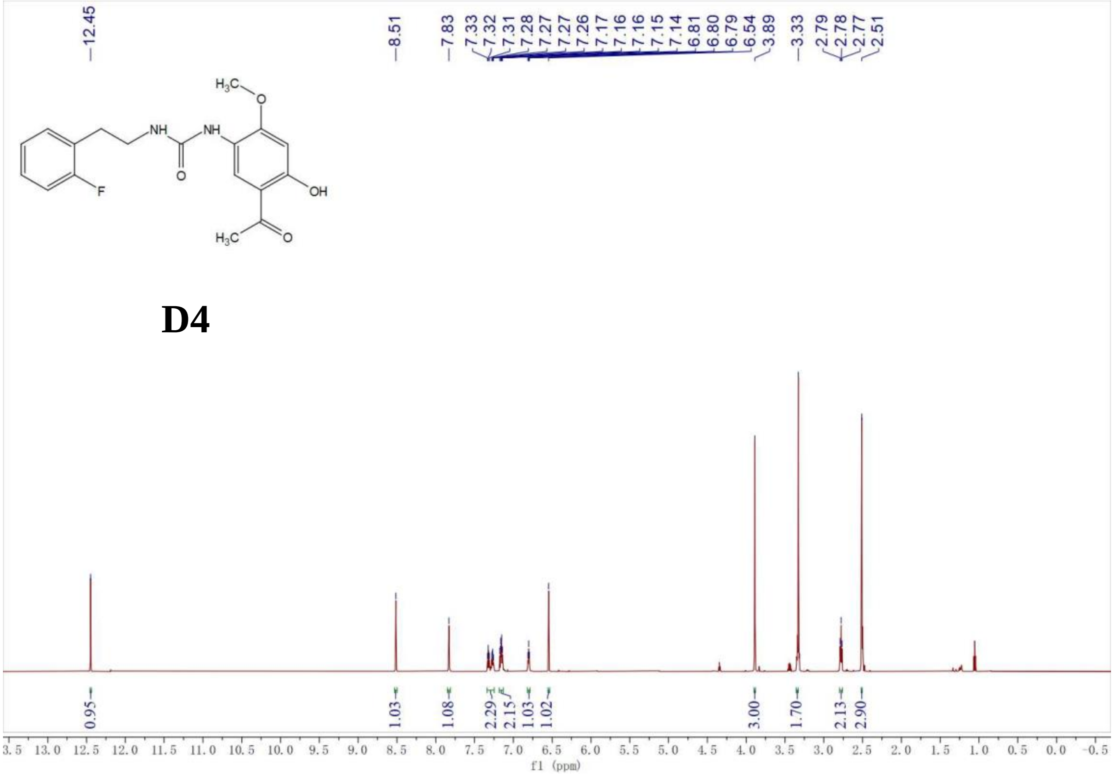

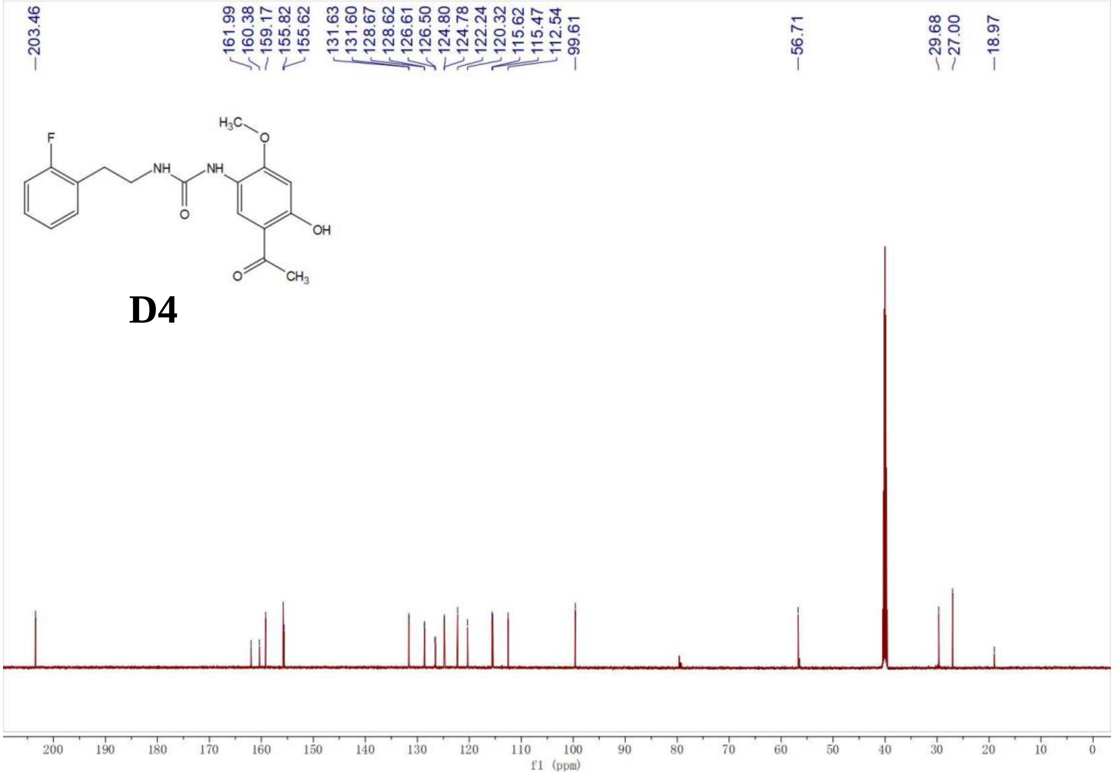

HRMS of compound **D4**

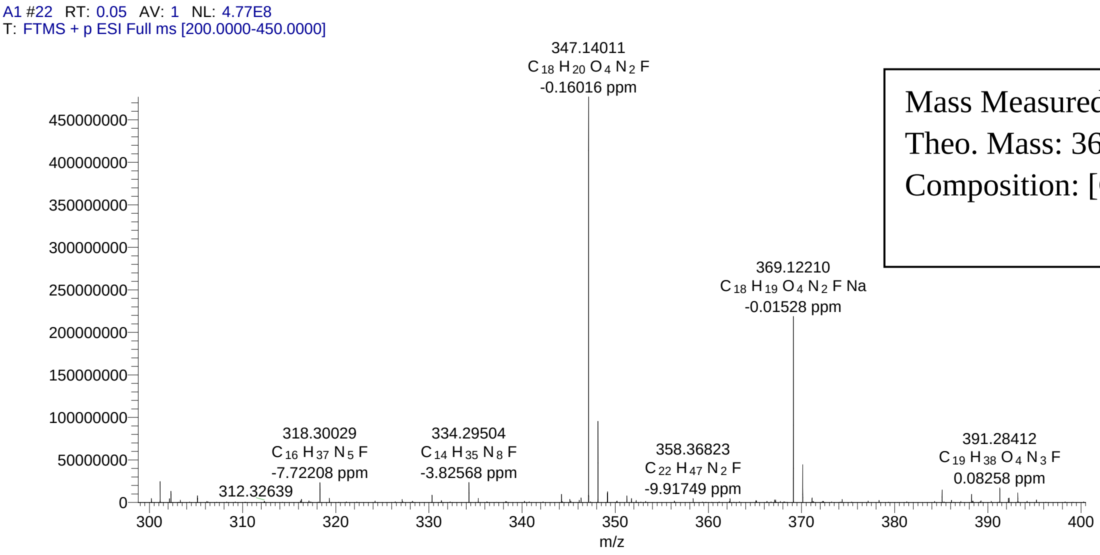

Mass Measured: 369.1221
Theo. Mass: 369.1221
Composition: $[C_{19}H_{22}N_2O_5]^+$

3. Purity data analyzed by HPLC of Compounds

Purity of compound was determined by analytical HPLC. Analysis conditions: Shimadzu LC-20AD/T HPLC machine fitted with an Inertex C_{18} column (4.6 mm×150 mm, 5 μm particle size) with CH_3OH (A) 60% methanol and 40% water solvent mixtures and equipped with a G1314A VWD detector. Flow rate 1.0 mL/min; UV detection at 254 nm.

B1

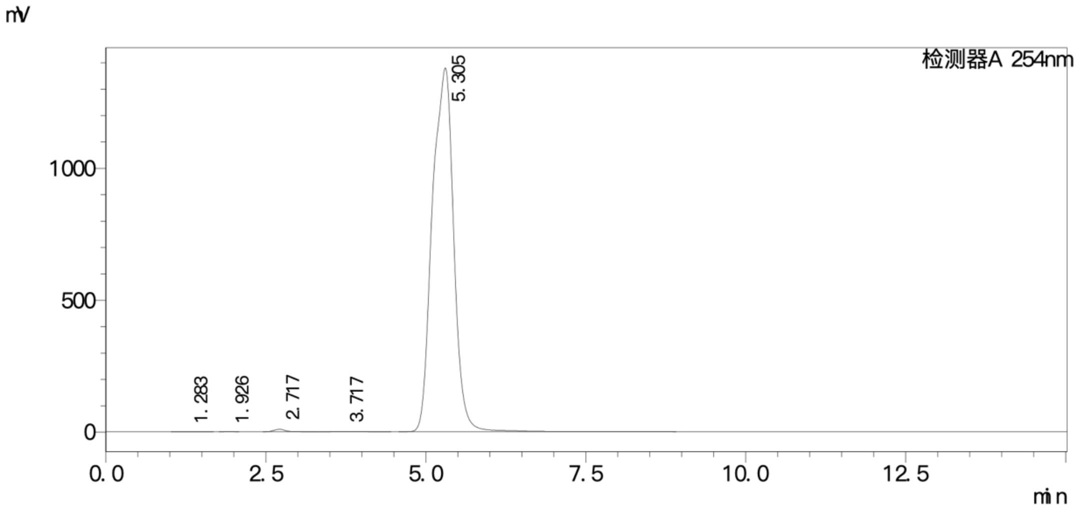

No.	Ret Time (min)	Area (mV*min)	Height (mV)	Rel.Area (%)
1	1.283	2190	133	0.006816559
2	1.926	4328	501	0.013471263
3	2.717	107844	8895	0.335673488
4	3.717	10913	304	0.033967627
5	5.305	32002374	1378619	99.61007106
Total		32127649	1388452	

B4

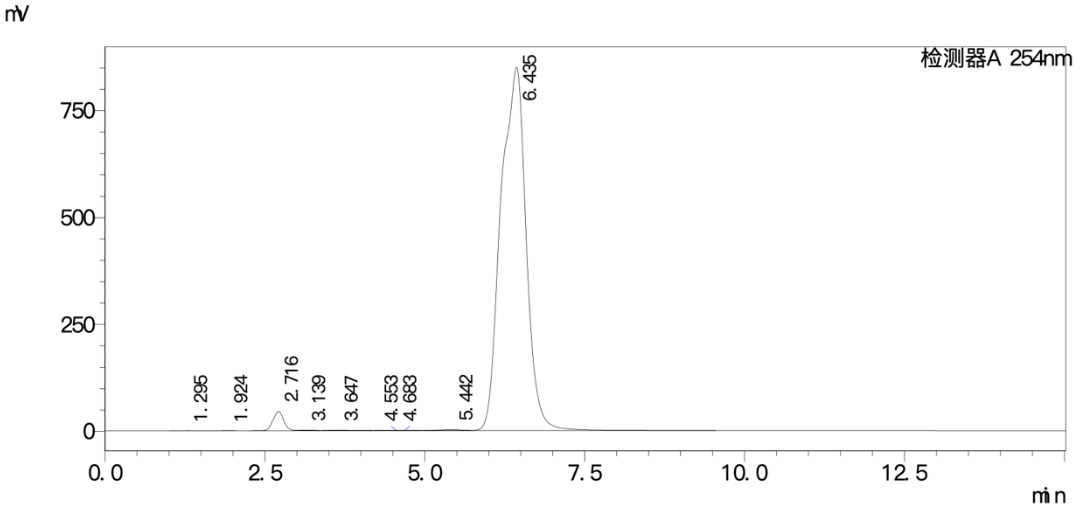

No.	Ret Time (min)	Area (mV*min)	Height (mV)	Rel.Area (%)
1	1.295	2162	126	0.009010637
2	1.924	4568	504	0.0190382
3	2.716	583663	44903	2.432550988
4	3.139	1919	223	0.007997878
5	3.647	12575	610	0.05240923
6	4.553	5898	457	0.024581284
7	4.683	4881	448	0.0203427
8	5.442	42682	1881	0.177887139
9	6.435	23335517	849733	97.25618194
Total		23993865	898884	

B5

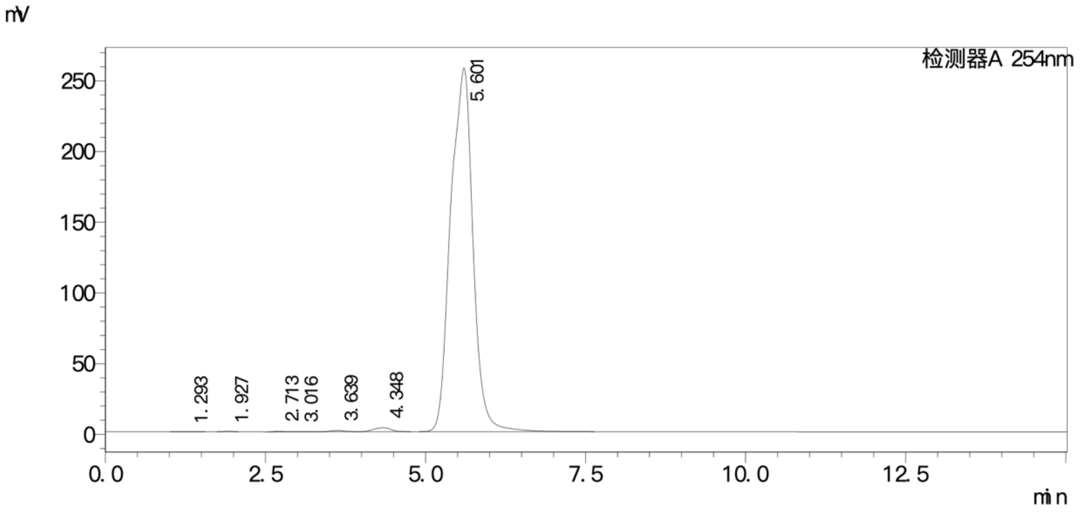

No.	Ret Time (min)	Area (mV*min)	Height (mV)	Rel.Area (%)
1	1.293	1422	91	0.022555039
2	1.927	4245	488	0.067332024
3	2.713	4748	401	0.075310354
4	3.016	1331	90	0.021111643
5	3.639	14325	884	0.227215842
6	4.348	51888	2818	0.823020986
7	5.601	6226619	257205	98.76345411
Total		6304578	261977	

B8

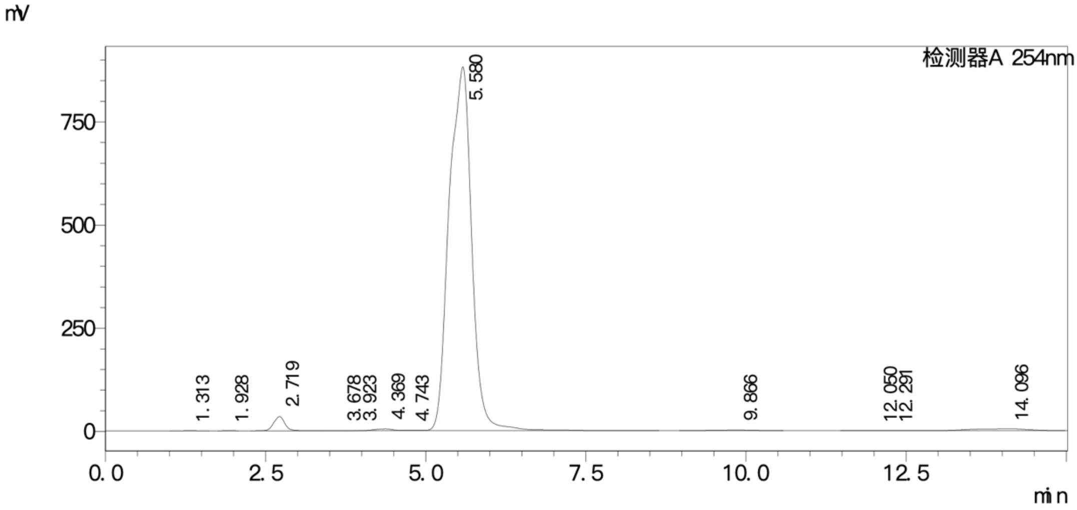

No.	Ret Time (min)	Area (mV*min)	Height (mV)	Rel.Area (%)
1	1.313	3100	266	0.013749206
2	1.928	4732	532	0.020987497
3	2.719	421919	34443	1.871306814
4	3.678	9072	541	0.040236385
5	3.923	8524	563	0.03780588
6	4.369	89181	4568	0.395538037
7	4.743	16540	947	0.073358665
8	5.58	21649396	882005	96.01999968
9	9.866	48858	1152	0.216696352
10	12.05	2668	128	0.011833187
11	12.291	4860	161	0.021555206
12	14.096	287908	5011	1.276937521
Total		22546757	930316	

B9

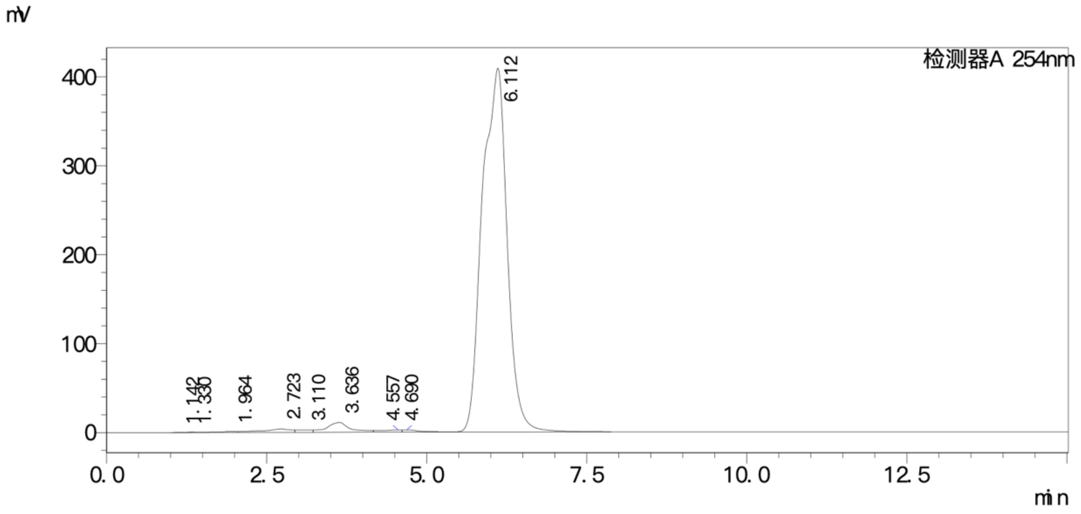

No.	Ret Time (min)	Area (mV*min)	Height (mV)	Rel.Area (%)
1	1.142	1253	148	0.010807661
2	1.33	4310	564	0.037175593
3	1.964	21541	1232	0.185800338
4	2.723	120286	3812	1.037518195
5	3.11	45535	2693	0.39275885
6	3.636	274830	11037	2.370526293
7	4.557	57673	2533	0.497454291
8	4.69	49562	2553	0.427493447
9	6.112	11018638	409270	95.04046533
Total		11593628	433843	

B11

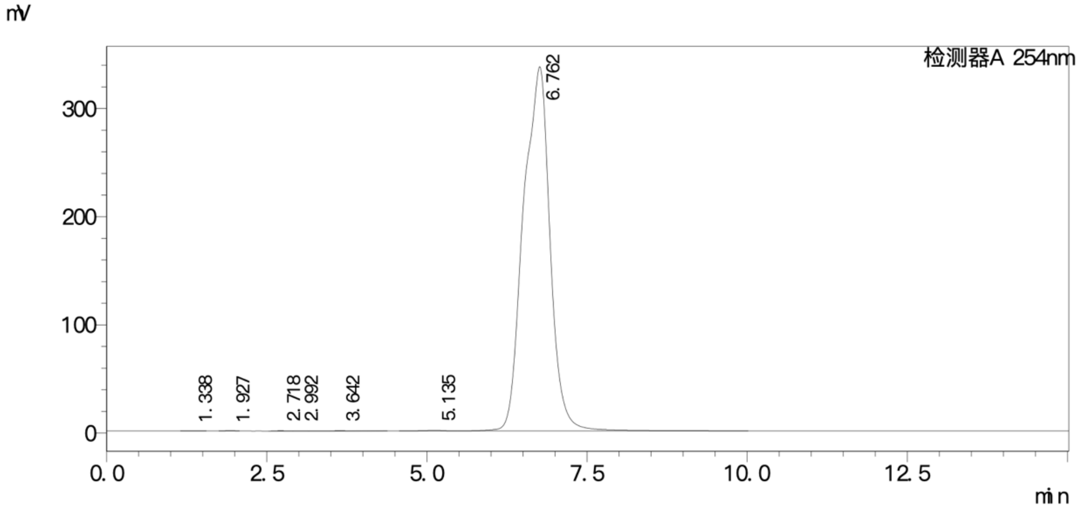

No.	Ret Time (min)	Area (mV*min)	Height (mV)	Rel.Area (%)
1	1.338	1667	159	0.017074974
2	1.927	3948	458	0.040439111
3	2.718	4222	340	0.043245675
4	2.992	1311	97	0.013428489
5	3.642	9346	282	0.095730478
6	5.135	12421	539	0.127227506
7	6.762	9729911	336935	99.66285377
Total		9762826	338811	

B12

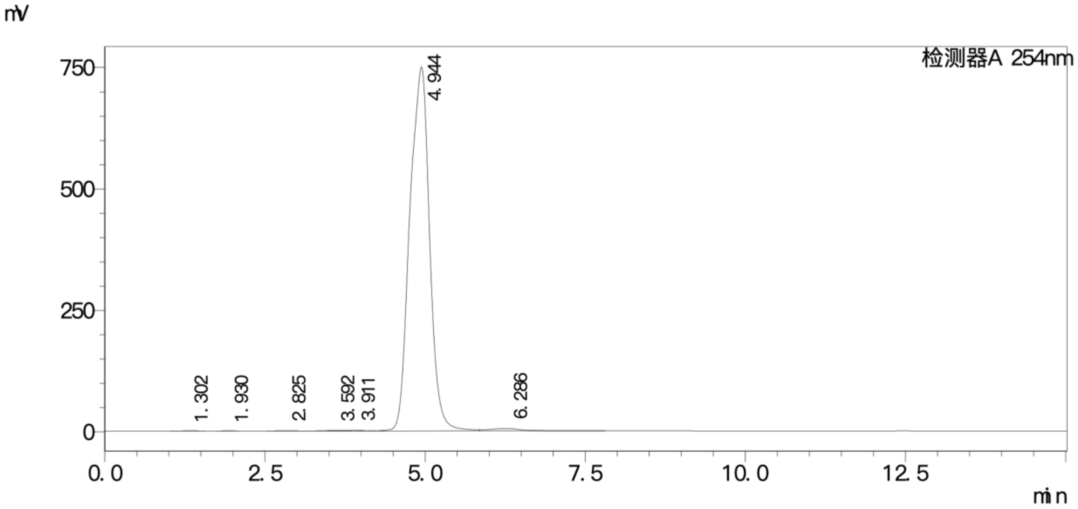

No.	Ret Time (min)	Area (mV*min)	Height (mV)	Rel.Area (%)
1	1.302	2019	132	0.012372756
2	1.93	4566	518	0.027981182
3	2.825	7144	476	0.04377958
4	3.592	15674	972	0.09605279
5	3.911	22646	1279	0.138778327
6	4.944	16090066	749369	98.60250973
7	6.286	175994	4902	1.07851951
Total		16318110	757646	
